# Comparative analysis of *Dendrobium* plastomes and utility of plastomic mutational hotspots

**DOI:** 10.1038/s41598-017-02252-8

**Published:** 2017-05-18

**Authors:** Niu Zhitao, Zhu Shuying, Pan Jiajia, Li Ludan, Sun Jing, Ding Xiaoyu

**Affiliations:** College of Life Sciences, Nanjing Normal University, Nanjing, China

## Abstract

*Dendrobium* is one of the largest genera in Orchidaceae, comprising about 800–1500 species mainly distributed in tropical Asia, Australasia, and Australia. There are 74 species and two varieties of this genus in China. Because of their ornamental and commercial value, *Dendrobium* orchids have been studied at low taxonomic levels. However, structural changes and effective mutational hotspots of *Dendrobium* plastomes have rarely been documented. Here, 30 *Dendrobium* plastomes were compared, comprising 25 newly sequenced in this study and five previously published. Except for their differences in NDH genes, these plastomes shared identical gene content and order. Comparative analyses revealed that the variation in size of *Dendroubium* plastomes was associated with dramatically changed length of InDels. Furthermore, ten loci were identified as the top-ten mutational hotspots, whose sequence variability was almost unchanged with more than 10 plastomes sampled, suggesting that they may be powerful markers for *Dendrobium* species. In addition, primer pairs of 47 polymorphic microsatellites were developed. After assessing the mean BS values of all combinations derived from the top-ten hotspots, we recommend that the combination of five hotspots—*trnT*-*trnL*, *rpl32*-*trnL*, *clpP*-*psbB*, *trnL intron*, and *rps16*-*trnQ*—should be used in the phylogenetic and identification studies of *Dendrobium*.

## Introduction

*Dendrobium*, a genus of the tribe Dendrobieae (Orchidaceae: Epidendroideae), is one of the largest genera in Orchidaceae with approximately 800–1500 species mainly distributed in tropical Asia, Australasia, and Australia^1, 2^. There are 74 species and two varieties of this genus in China^3^, some of which are well known as flowers of Father’s Day in many Asian countries. *Dendrobium* orchids are popular not only for their aesthetic appeal, primarily reflected in their unique flower characteristics, but also for their medicinal value. Owing to their strong health care effects, such as nourishing the kidney, benefiting the stomach, enhancing the body’s immunity, resisting cancer, and prolonging life, many species in this genus have been extensively used as Traditional Chinese Medicine (TCM) for hundreds of years^4^. However, many wild *Dendrobium* species are in extreme danger of extinction (IUCN Redlist of higher plants in China, http://www.zhb.gov.cn/gkml/hbb/bgg/201309/t20130912_260061.htm) due to their low germination rate, slow growing, habitat deterioration, and being over-exploited.

Because of their ornamental and commercial value, *Dendrobium* orchids have attracted intense attention of reseachers, leading to numerous taxonomic studies published, particularly in species identification^3, 5, 6^. However, *Dendrobium* species are notoriously difficult to identify. Traditional methods for identifying *Dendrobium* species are based on their morphological characteristics, while many species have overlapping morphological variations due to environmental factors and pollinator selection pressure^2, 7, 8^. Furthermore, after intensively processed, the shoots of *Dendrobium* species become more difficult to distinguish^9^. Therefore, it is urgent to develop a simple and accurate method for identification of *Dendrobium* species.

Recently, a variety of molecular markers have been developed for the studies of *Dendrobium* in terms of species identification, population genetics, and phylogeny. Microsatellite (SSR) markers have been employed to study population genetics of *Dendrobium* species and to investigate the species relationships^10, 11^. Random amplified polymorphic DNA (RAPD) and amplified fragment length polymorphism (AFLP) markers are also available for *Dendrobium* ^12, 13^. In addition, DNA barcode has been adopted to identify *Dendrobium* species, involving different loci or their combinations, e.g. ITS^14, 15^, ITS2^5^, ITS + *matK* ^6^, and *rbcL* + *matK* ^16^. However, many of these studies resulted in inconsistent conclusions because of using limited number of DNA sequences.

The chloroplast is one of the essential organelles in plant cells, having its own genome called plastome. Plastomes are an ideal resource for selecting mutational hotspots in various lineages because of their maternal mode of inheritance, dense gene content, and slower evolutionary rates relative to those of nuclear and mitochondrial genomes^17, 18^. A number of hotspots, including *rbcL*, *matK*, and *psbA*-*trnH*, have been successfully applied to plant species identification and phylogenetic studies^19–21^. Recently, the comparative plastomic method has been available for mutational hotspot selection, which uses at least two complete plastomes within the study genus to screen for the most informative regions^22, 23^. For instance, the *psbA*-*trnH* and *trnF*-*ndhJ* regions in orchids were demonstrated to be the most useful markers for the phylogenetic analysis of *Oncidium* ^24^; and the noncoding loci *rpl32-trnL*, *trnE-trnT*, *trnH-psbA*, *trnK-rps16*, and *trnT-trnL* were shown to be effective in identifying species of *Cymbidium* ^25^. However, comprehensive plastome-wide investigation has not been conducted on more powerful loci, which, however, are important for low taxonomic level studies of *Dendrobium* species.

In this investigation, we compared 30 plastomes of important *Dendrobium* species that contents great medical worth, including the 25 newly sequenced. Our aims were: (1) to evaluate the evolution of *Dendrobium* plastomes; (2) to identify more powerful mutational hotspots for low taxonomic level studies of *Dendrobium* species on the basis of a wide range of sampling. To achieve these aims, the Maximum likelihood (ML) approach was adopted to evaluate potential hotspot combinations by assessing their mean bootstrap (BS) values.

## Result

### Genome features

The 25 newly sequenced *Dendrobium* plastomes ranged from 150,073 to 152,108 bp in length, with the smallest one belonging to *D*. *parciflorum* while the largest falling into *D*. *fanjingshanense* (Table 1). All plastomes possessed the ancestral angiosperm plastome organization that consisted of a LSC region of 84,273–84,990 bp, a SSC region of 13,821–14,514 bp, and a pair of IR regions of 26,175–26,309 bp each (Table 1, Figure S1a). Similar to other orchid plastomes, *Dendrobium* plastomes were also AT-rich (62.27–62.69%). Except for their differences in the total length and composition of retained NDH genes, all plastomes shared identical complements of coding genes, each containing 30 unique tRNA genes, four unique rRNA genes, and 68 unique protein-coding genes. The sequence of eleven NDH genes of *Dendrobium* species were compared to *Cypripedium formosanum* (NC_026772), which contains full set of functional NDH genes in orchids (Figure S1b). However, like other Epidendroideae species (e.g. *Cymbidium*, *Oncidium*, and *Phalaenopsis*), *Dendrobium* also experienced the loss of plastid NDH genes. Among them, only *ndhB* genes in IR regions were functional with full reading frames, whereas other ten plastid NDH genes were truncated or completely lost.

**Table 1:** Characteristics of the 25 newly sequenced *Dendrobium* plastomes.

Species Name	Plastome length (bp)	LSC region (bp)	SSC region (bp)	IR region (bp)	AT content (%)	Accession	No. vouchers specimen
*Dendrobium aphyllum*	151524	84588	14320	26308	62.40%	LC192953	NZT2015001
*Dendrobium brymerianum*	151830	84855	14377	26299	62.40%	LC192954	NZT2015002
*Dendrobium chrysanthum*	151790	84757	14441	26296	62.44%	LC193514	NZT2015003
*Dendrobium chrysotoxum*	151731	84785	14356	26295	62.37%	LC193517	NZT2015004
*Dendrobium crepidatum*	151717	84811	14383	26262	62.43%	LC193509	NZT2015005
*Dendrobium denneanum*	151565	84657	14344	26282	62.37%	LC192955	NZT2015006
*Dendrobium devonianum*	151945	84966	14435	26272	62.45%	LC192956	NZT2015007
*Dendrobium ellipsophyllum*	152026	84930	14488	26304	62.50%	LC193519	NZT2015008
*Dendrobium exile*	151294	84363	14315	26308	62.32%	LC193522	NZT2015009
*Dendrobium falconeri*	151890	84862	14448	26290	62.51%	LC192957	NZT2015010
*Dendrobium fanjingshanense*	152108	84990	14514	26302	62.49%	LC193523	NZT2015011
*Dendrobium fimbriatum*	151673	84763	14328	26291	62.40%	LC193521	NZT2015012
*Dendrobium gratiosissimum*	151829	84890	14359	26290	62.43%	LC192958	NZT2015013
*Dendrobium henryi*	151850	84878	14366	26303	62.44%	LC193513	NZT2015014
*Dendrobium hercoglossum*	151939	84924	14397	26309	62.44%	LC192959	NZT2015015
*Dendrobium jenkinsii*	151717	84734	14413	26285	62.40%	LC193515	NZT2015016
*Dendrobium lohohense*	151812	84876	14352	26292	62.44%	LC193516	NZT2015017
*Dendrobium parciflorum*	150073	83708	13821	26272	62.33%	LC193512	NZT2015018
*Dendrobium parishii*	151689	84703	14396	26295	62.42%	LC193518	NZT2015019
*Dendrobium primulinum*	150767	84442	13975	26175	62.27%	LC192810	NZT2015020
*Dendrobium salaccense*	151104	84273	14315	26258	62.69%	LC193510	NZT2015021
*Dendrobium spatella*	151829	84794	14419	26308	62.42%	LC193511	NZT2015022
*Dendrobium wardianum*	151788	84835	14359	26297	62.43%	LC192961	NZT2015023
*Dendrobium wilsonii*	152080	84988	14480	26306	62.49%	LC193508	NZT2015024
*Dendrobium xichouense*	152052	84980	14486	26293	62.49%	LC193520	NZT2015025

### InDels coincide with the variation of plastome

Thirty plastomes of *Dendrobium*, including our newly sequenced 25, were complied for comparison. These plastomes experienced different degrees of NDH gene loss, in which the total length of retained NDH genes varied from 3,687–6,336 bp (Table S1). On the other side, the total length of retained NDH genes was uncorrelated with the plastome length (Spearman’s *r* = 0.163, *P* > 0.05). In addition, the changed lengths of LSC, SSC, IRs, and whole plastome were compared between each tested species and *D*. *officinale*. Our analysis indicated that the changed length of LSC, which retains only a few *ndh* residues, was strongly correlated with the changed length of plastome (Spearman’s *r* = 0.908, *P* < 0.01). Meanwhile, the changed lengths of SSC (retaining most of the NDH genes) and IRs (its expansion/contraction having a direct impact on plastome size) were medially correlated with the changed length of *Dendrobium* plastome (Spearman’s *r* = 0.634, 0. 721, *P* < 0.05). These results suggested that the changed length of LSC occupied an important position in the changes of plastome sizes.

**Figure 1:**
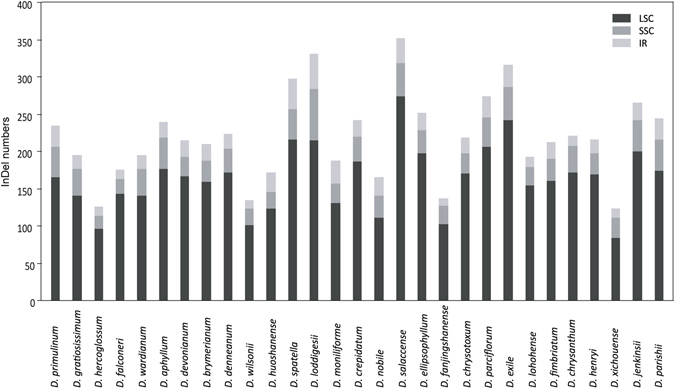
InDel distribution among different *Dendrobium* plastomes. The InDel mutations were determined based on the comparison between plastoms of each tested *Dendrobium* species and *D*. *officinale*. Histograms with different colors indicate the numbers of InDels in LSC, SSC, and IR regions.

InDel mutations in plastome were compared between each tested *Dendrobium* species and *D*. *officinale* (Fig. 1). As a result, a total of 123–352 InDels were identified among these plastomes, with 84–274 in LSC, 18–69 in SSC, and 10–47 in IRs. The InDels located in LSC region accounted for 65–82%; this proportion was significantly greater than those for the InDels situated in SSC and IRs (Mann-Whitney 2-sides, *P* < 0.05), indicating that the locations of InDels in plastome were nonrandom. In order to evaluate the relationship between the variation of *Dendrobium* plastome size and InDel changes, we determined the changed length of InDels based on the differences between insertions and deletions and divided them into two parts: NDH gene-related InDels change and NDH gene-unrelated InDels change (Table S2). The changed length of NDH gene-unrelated InDels was significantly larger than that of NDH gene-related InDels, which was caused by the loss of NDH genes (Mann-Whitney 2-sides, *P* < 0.05). Moreover, the changed length of NDH gene-unrelated InDels was strongly correlated with the variation of plastome size (Spearman’s *r* = 0.867, *P* < 0.01), suggesting that the variation of *Dendrobium* plastome size was largely due to the changed length of InDels.

### Mutational hotspots in *Dendrobium* plastomes

**Figure 2:**
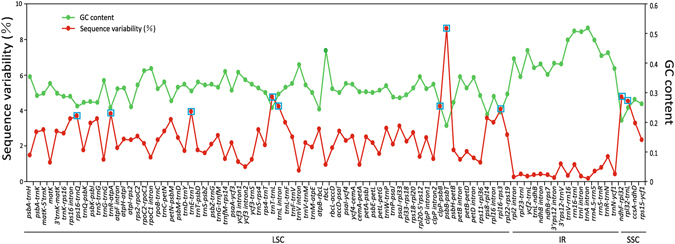
The sequence variability (%) and GC content among the 92 syntenic intergenic and intronic loci from *Dendrobium* plastomes. The red and green lines represent the sequence variability (%) and GC content of each locus, respectively. These syntenic loci are oriented according to their locations in the plastome. The top-ten syntenic intergenic and intronic loci with the highest sequence variability (%) in the tested *Dendrobium* plastomes were indicated with blue box.

We identified 92 syntenic intergenic and intronic loci, each longer than 150 bp. Three of them (*matK*, *rbcL* and *psbA*-*trnH*) had been widely used as DNA barcode owing to their high variability. Sequence variability (SV) was calculated for each of these loci (Fig. 2 and Table S3). It has been reported that plastomic mutational hotspots are accompanied by biased AT compositions. Consistently, our study showed that the SV of a locus was negatively correlated with its GC content (Spearman’s *r* = −0.809, *P* < 0.01).

**Figure 3:**
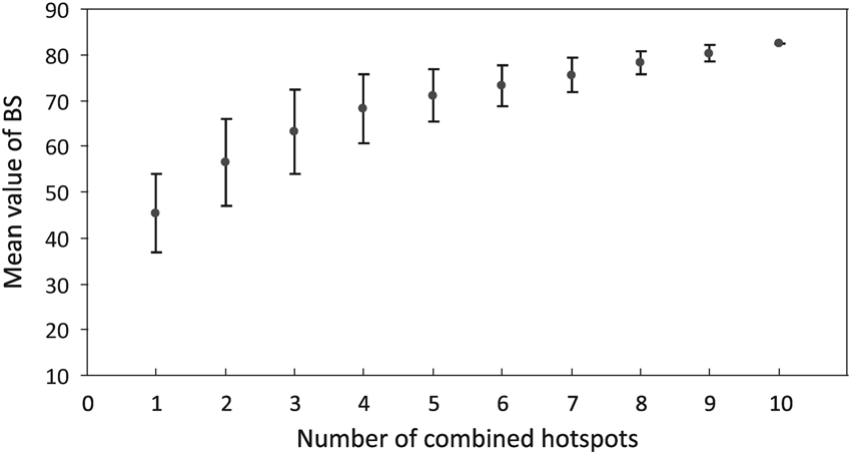
Relation between the number of combined hotspots and the mean value of bootstrap (BS). The mean (±SD) BS values were calculated based on different numbers of combined hotspots. Regression analysis: R^2^ = 0.904, y = 3.64x + 49.38. The plateau of the mean BS value reached 71% when five hotspots were combined.

Figure 3 shows the SV of the top-ten mutational hotspots from the 25 newly sequenced plastomes. All of these hotspots except *trnL* intron were intergenic spacers. To examine whether the SV of these hotspots changes with increasing number of sampled plastomes, we evaluated their SV rankings among six groups that were randomly composed of different numbers of *Dendrobium* plastomes (Table 2). Only five to six of these mutational spots ranked in the top ten hotspots when sampled plastomes were fewer than ten. However, when more than ten plastomes were sampled, these mutational spots consistently ranked in the top ten.These results indicated that the consistency of the SV of these mutational spots rose with increasing number of sampled plastomes. Therefore, the top ten hotspots (Fig. 2) could be powerful markers for phylogenetic and identification studies of *Dendrobium* species.

**Table 2:** SV rankings of the top-ten mutational hotspots in six groups.

	Group A (2 species)	Group B (5 species)	Group C (10 species)	Group D (15 species)	Group E (20 species)	Group F (25 species)
*psbB-psbT*	1	1	1	1	1	1
*ndhF-rpl32*	—	3	3	2	2	2
*trnT-trnL*	3	2	2	3	3	3
*rpl32-trnL*	—	—	6	4	4	4
*clpP-psbB*	—	—	—	6	5	5
*trnL intron*	4	4	4	5	6	6
*rpl16-rps3*	—	—	5	7	7	7
*trnE-trnT*	9	—	7	9	8	8
*trnR-atpA*	6	—	—	—	9	9
*rps16-trnQ*	7	9	9	10	10	10

“—” Means this locus ranked outside of the top-ten mutational hotspots.

Group A: *D*. *primulinum*, *D*. *gratiosissimum*.

Group B: *D*. *primulinum*, *D*. *gratiosissimum*, *D*. *hercoglossum*, *D*. *falconeri*, *D*. *wardianum*.

Group C: *D*. *primulinum*, *D*. *gratiosissimum*, *D*. *hercoglossum*, *D*. *falconeri*, *D*. *wardianum*, *D*. *aphyllum*, *D*. *devonianum*, *D*. *brymerianum*, *D*. *denneanum*, *D*. *wilsonii*.

Group D: *D*. *primulinum*, *D*. *gratiosissimum*, *D*. *hercoglossum*, *D*. *falconeri*, *D*. *wardianum*, *D*. *aphyllum*, *D*. *devonianum*, *D*. *brymerianum*, *D*. *denneanum*, *D*. *wilsonii D*. *spatella*, *D*. *crepidatum*, *D*. *salaccense*, *D*. *ellipsophyllum*, *D*. *fanjingshanense*.

Group E: *D*. *primulinum*, *D*. *gratiosissimum*, *D*. *hercoglossum*, *D*. *falconeri*, *D*. *wardianum*, *D*. *aphyllum*, *D*. *devonianum*, *D*. *brymerianum*, *D*. *denneanum*, *D*. *wilsonii D*. *spatella*, *D*. *crepidatum*, *D*. *salaccense*, *D*. *ellipsophyllum*, *D*. *fanjingshanense*, *D*. *chrysotoxum*, *D*. *parciflorum*, *D*. *exile*, *D*. *lohohense*, *D*. *fimbriatum*.

Group F: *D*. *primulinum*, *D*. *gratiosissimum*, *D*. *hercoglossum*, *D*. *falconeri*, *D*. *wardianum*, *D*. *aphyllum*, *D*. *devonianum*, *D*. *brymerianum*, *D*. *denneanum*, *D*. *wilsonii D*. *spatella*, *D*. *crepidatum*, *D*. *salaccense*, *D*. *ellipsophyllum*, *D*. *fanjingshanense*, *D*. *chrysotoxum*, *D*. *parciflorum*, *D*. *exile*, *D*. *lohohense*, *D*. *fimbriatum*, *D*. *chrysanthum*, *D*. *henryi*, *D*. *xichouense*, *D*. *jenkinsii*, *D*. *parishii*.

### Microsatellites

We totally retrieved 47 polymorphic SSRs, which are present in at least 15 species, from 92 syntenic intergenic and intronic loci (Table 3). These SSRs consisted of two types: mononucleotide SSRs (44 A/T type and one C/G type), ranging from 8 to 16 nucleotide repetitions; and dinucleotide SSRs (TA)_6_. Six of them (*trnT*-*trnL*, *trnL* intron, *trnE*-*trnT*, *trnR*-*atpA*, and *rps16*-*trnQ*) were among the top ten hotspots. The SSRs were mainly distributed in LSC, while only one was located in SSC and three in IRs. This signified that the distribution of SSRs was dependent on their locations in plastomes. Our result also revealed that 37 SSRs were located in intergenic spacer regions and 10 SSRs in introns. Primer pairs were developed for all the SSRs (Table 3), which could be used in the amplification of SSRs in *Dendrobium* species for future studies.

**Table 3:** Polymorphic SSRs identified in the comparative analysis of *Dendrobium* plastomes.

NO.	Primer name	Position	Region	Location	SSR Type	Primer sequence (5′-3′)	Length	Tm
1	Den ptssr1	*trnT*-*trnL*	LSC	spacer	AT × 6	AGAAATTCAATTCCATATTCA	232	51
						CATTGATGTATCCGCAATAT		
2	Den ptssr2	*trnT*-*trnL*	LSC	spacer	AT × 6	CTAAATAGAAATTCAATTCCT	185	53
						CCTTTACCCCTCCTTCCTAA		
3	Den ptssr3	*trnL* intron	LSC	intron	T × (8–14)	TGGATTGAGCCTTGGTATA	231	50
						TCCTTTCTGTCATTTCGATT		
4	Den ptssr4	*trnE*-*trnT*	LSC	spacer	A × (8–10)	AATATGAATCTTACCCACTTCC	180	52
						TGAACCGATGACTTACGCAA		
5	Den ptssr5	*trnR*-*atpA*	LSC	spacer	A × 9	TTGGACGCATTTATTTCTAC	250	52
						CGAAGAAGCTGAAACCCTT		
6	Den ptssr6	*rps16*-*trnQ*	LSC	spacer	A × (8–13)	AAAGTCTCGTGTAAGGTAT	225	53
						ATGTTGGATACACTGAATA		
7	Den ptssr7	*rps16* intron	LSC	intron	T × (8–11)	CTCTTTCTATCATCCTTCCAT	225	51
						CCCACTATAAACTTAGTAACTAT		
8	Den ptssr8	*petN*-*psbM*	LSC	spacer	A × (8–10)	TTCACTTGTAGTATGGGGAAG	232	50
						GAGGATTAAATAGAAGAATCT		
9	Den ptssr9	*trnL*-*trnF*	LSC	spacer	T × (8–15)	TTCCTCGCTCTTTATTTATCC	209	54
						CAATAACGGAGATTCCTTGAA		
10	Den ptssr10	*rps8*-*rpl14*	LSC	spacer	T × (8–11)	TAGTTATTGGTGTCTCCTCAT	160	51
						TATCTGAAATAGATCCGATTA		
11	Den ptssr11	*psbK*-*psbI*	LSC	spacer	A × (8–10)	AAGGAAATCTCGATTCAATTC	241	53
						AAAGGAAAGGTCAGAACAAAA		
12	Den ptssr12	*psbK*-*psbI*	LSC	spacer	A × (8–10)	CTTTAATCAGCTAATCAACTT	225	50
						CTATTTGATATGAAGCTCTAA		
13	Den ptssr13	*psbK*-*psbI*	LSC	spacer	A × (8–10)	AGATATGGATATGGCAAGAAA	217	52
						TACAAATCTCCAAGATAAGAT		
14	Den ptssr14	*ccsA*-*ndhD*	SSC	spacer	T × (8–11)	AAATCGTCTGATACGCAATGC	174	56
						TTGACTTTCATATTTTCACGA		
15	Den ptssr15	*rps16* intron	LSC	intron	T × (8–11)	AACTCAAGTTGGGTAGTTTTG	224	51
						TAAGGATCACCGAAGTAATGT		
16	Den ptssr16	*psbA*-*trnK*	LSC	spacer	T × (8–14)	CTATGCCAATGTCAACCAATC	246	55
						CTTTCTTTAATCTTCCTCCAA		
17	Den ptssr17	*matK*-*5*′*trnK*	LSC	intron	A × (8–10)	AATCACTCTTTTGACTTTGGAA	214	54
						AATTTGAATGATTACCCGTAC		
18	Den ptssr18	*matK*-*5*′*trnK*	LSC	intron	A × (8–10)	CTTACTCGAATTGGAGCCATA	216	55
						CCGCGACTGATCCTGAAAGGT		
19	Den ptssr19	*atpB*-*rbcL*	LSC	spacer	T × (8–14)	ATAGCAAGTTGATCGGTTAAT	224	51
						CTAGATGTGAAAAGAGGCATA		
20	Den ptssr20	*atpB*-*rbcL*	LSC	spacer	T × (8–14)	TTCTATCTTTATCTTTACTTTCG	262	50
						GAGTATGAAGAATAATGAATATGA		
21	Den ptssr21	*atpB*-*rbcL*	LSC	spacer	T × (8–14)	CTATCTTTATCTTTACTTTCG	255	50
						GAAGAATAATGAATATGATAGA		
22	Den ptssr22	*trnC*-*petN*	LSC	spacer	A × (8–12)	ATCCTGTTGATCGAACTTGAC	216	54
						CAATTCAGAATAGCCCAAACC		
23	Den ptssr23	*trnC*-*petN*	LSC	spacer	A × (8–12)	ACTGATTTGTATCCAGACTCA	218	50
						TCTTACTTACGGCTCTTTATG		
24	Den ptssr24	*trnC*-*petN*	LSC	spacer	A × (8–12)	ACTAGAGGCTCTGAGTGCTGC	235	55
						TCATAGTGGAATGAATGGTGC		
25	Den ptssr25	*rps18*-*rpl20*	LSC	spacer	A × 8	AAACTCCAATAGGAAATCAAG	213	52
						ACAAGAATGATTGAAACAGGA		
26	Den ptssr26	*petA*-*psbJ*	LSC	spacer	T × (8–14)	AATAAAGTTGGTAAAAGTGCC	189	51
						TCCTTTGTATTTGTATGCTTC		
27	Den ptssr27	*ycf4*-*cemA*	LSC	spacer	T × (8–14)	AGGAAGAAAAGAAGAGGAAATC	217	54
						CCTATAACTCTAACAAGAACAA		
28	Den ptssr28	*rps2*-*rpoC2*	LSC	spacer	T × (9–13)	CCATTTATTAGTACCATGACCA	207	55
						CTAATACCTAAAGCATTAGTTA		
29	Den ptssr29	*trnF*-*trnV*	LSC	spacer	A × (8–9)	ATTGAGACGGATCGGGATAGA	229	56
						GCAAAATGATAAGAATCGGAG		
30	Den ptssr30	*atpI*-*rps2*	LSC	spacer	A × (8–10)	ATTATTTTGATTCAACCATCTC	189	51
						GATTGTTACTCTTTTGGTTTG		
31	Den ptssr31	*psbZ*-*trnG*	LSC	spacer	A × (8–10)	CCGATCCAAATAATCCTTCTA	264	54
						TTTTCTTCGTTCCTGATACGT		
32	Den ptssr32	*psaA*-*ycf3*	LSC	spacer	A × (8–11)	ATGAGATACCGTAGAAAATGT	240	51
						CTGCTGAGTATTGGAAACAAG		
33	Den ptssr33	*psbE*-*petL*	LSC	spacer	T × (8–13)	GCTCCACAAATTCTTGTATGT	203	53
						AATTTCCTTTCGGTAATGATC		
34	Den ptssr34	*psbE*-*petL*	LSC	spacer	T × (8–13)	ATTAGTGGCTTCATCATAGTAAT	244	50
						CAAAGTGAAATAGTGTATTAGCAT		
35	Den ptssr35	*psbE*-*petL*	LSC	spacer	T × (8–13)	ACTTTGAAATTAGAAACTGAAGCTA	232	54
						ACAACAGTTGCATCACGAATA		
36	Den ptssr36	*psbE*-*petL*	LSC	spacer	T × (8–13)	TTCTTTTGAATCGAGTTGGTCC	207	56
						TTTCAATCCAGATACGACGGT		
37	Den ptssr37	*trnF*-*ndhJ*	LSC	spacer	A × (8–9)	TTCATTGAGACGGATCGGGATA	231	55
						CAAAATGATAAGAATCGGAGTT		
38	Den ptssr38	*trnD*-*trnY*	LSC	spacer	T × (8–10)	TTTCAGAAGAGCATTCTATTT	249	50
						CTCCATGAAGAAGATCTAAAG		
39	Den ptssr39	*trnS*-*psbZ*	LSC	spacer	A × (8–10)	GCTATCAACCACTCAGCCATC	247	55
						TCCTCCAAACTACCAACAAAT		
40	Den ptssr40	*rpoC1* intron	LSC	intron	T × (8–15)	CTACTCTTTACTCAAGTTCCCAA	202	55
						AAATCCTTTACGAGTCCCACA		
41	Den ptssr41	*petB* intron	LSC	intron	A × (8–11)	AACCTTTGAGTTTAGCTTTGG	185	53
						TACAATCTCAAGTTGGCTCAT		
42	Den ptssr42	*clpP* intron2	LSC	intron	A × (8–11)	GTTTGTGACGCTGAAATTGAC	200	55
						TACTATGCCTTCGCTGTATCG		
43	Den ptssr43	*clpP* intron2	LSC	intron	A × (8–11)	TCAAATTGGGAATAACTCTTC	228	51
						AATTACCAAACGTCTAGCATT		
44	Den ptssr44	*ycf3* intron1	LSC	intron	A × (8–11)	ATAGATGTAACCTTTTGCTCA	241	50
						AGGCATTTACCTATTACAGAG		
45	Den ptssr45	*3*′*rps12*-*trnV*	IR	spacer	T × (9–16)	CTTTGCCCCTCATTCTTCGAG	236	56
						ATGGGTCAGATTCTACAGGATCAAC		
46	Den ptssr46	*3*′*rps12*-*trnV*	IR	spacer	T × (9–16)	AGTAGTTAATGGTGGGGTTAC	248	52
						GCTCTATTCGAGACTGGTAGG		
47	Den ptssr47	*trnI* intron	IR	intron	G × (8–10)	TTCTCCTCAGGAGGATAGATG	223	53
						TCTGTGAAGATGCTGTGTTAG		

### Multi-hotspot combination anaylsis

**Figure 4:**
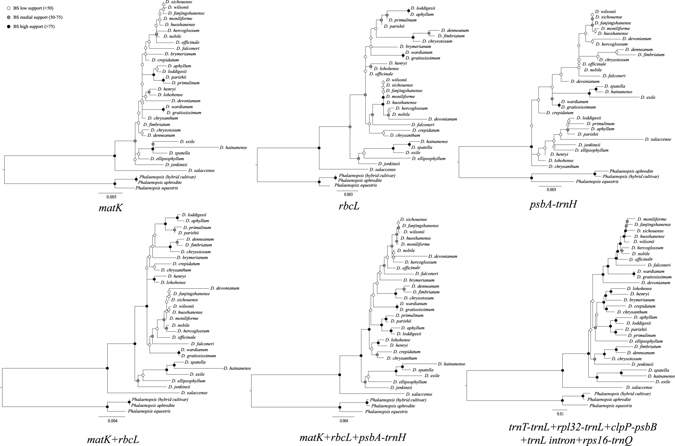
Maximum likelihood trees based on different plastid DNA data.

The top ten hotspots were retrieved from 30 *Dendrobium* plastomes, constituting 1,023 combinations (Table S4). To determine the optimal number of hotspots used for phylogenetic and identification studies of *Dendrobium*, we calculated the mean BS value of each ML tree based on these combinations. Correlation analyses indicated that the mean BS value was positively correlated to the SV and sequence length of hotspot combinations (Spearman’s *r* = 0.505, 0.6, *P* < 0.01). The mean BS values of ML trees climbed with increasing number of hotspots in a combination. On the other side, the variance among combinations declined with increasing number of hotspots in a combination; the greatest variations existed among the three combinations comprising one, two, and three hotspots (Fig. 3). The plateau of mean BS value reached 71% when five hotspots were combined, then rising slightly with further increasing number of hotspots in a combination. The top ten combinations that yielded highest BS values are shown in Table S5, of which only the fourth combination consisted of five hotspots (*trnT*-*trnL*, *rpl32*-*trnL*, *clpP*-*psbB*, *trnL* intron, and *rps16*-*trnQ*). Additionally, we also performed the phylogenetic analyses based on *rbcL*, *matK*, *psbA*-*trnH* and their combinations. Our results showed that the phylogenetic relationships based on the combination of *trnT*-*trnL*, *rpl32*-*trnL*, *clpP*-*psbB*, *trnL* intron, and *rps16*-*trnQ* had a better resolution than other plastid DNA data (Fig. 4).

## Discussion

In orchids, plastid NDH genes experienced independent loss^26, 27^. The dramatic NDH gene loss/retention has facilitated comparative plastome studies of orchid species^24, 26–29^. Recently, Kim *et al*.^27^ proposed that the loss of NDH genes led to the expansion/contraction of IRs^27^. It has been documented that independent loss/retention of NDH genes and expansion/contraction of IRs are largely responsible for the variation of plastomes in different orchid genera^26, 27^ and many other species, such as gnetophytes^30–32^, pines^30, 33^, slender naiad^34^, and saguaro^35^. However, our study demonstrated that neither NDH genes loss/retention nor the expansion/contraction of IRs accounted for the most important role in the variation of *Dendrobium* plastome sizes (Table S1).

Comparative analyses have shown that InDels commonly occur in plastomes. It is known that InDels are very useful for resolving phylogenetic relationship and can serve as biomarkers. For example, in the Pinaceae plastomes, nine InDels are able to resolve the phylogenetic relationships among different Pinaceae subfamilies^36^. In an investigation of the *Fagopyrum* plastomes, a number of InDel markers were identified and demonstrated to be effective in distinguishing raw or processed buckwheat products^37^. On the other side, few studies have given attention to the relationship between InDels and the variation of plastome size. In the present study, a strong correlation existed between the changed lengths of InDels and plastomes (Fig. 1, Table S2), demonstrating that the variation in size of *Dendroubium* plastomes was due to dramatical changes in lengths of InDels. However, the changed lengths of InDels is not secure to measure the phylogenetic relationship between the *Dendrobium* plastomes. In addition, according to Ahmed *et al*.^38^, the distribution of InDel events is dependent on their locations in plastomes^38^, e.g., associated with low GC content, high rate of nucleotide substitutions, or high frequency of SSRs; in line with this, the current research also revealed a nonrandom distribution of InDels (Fig. 1). Nevertheless, the dynamic distribution of the InDels in *Dendrobium* plastomes is worthy of further investigations.

Recently, the taxonomic study of *Dendrobium* has become a global concern of biological systematics and been regarded as one of the enormous challenges in Orchidaceae. Numerous studies have focused on searching the most appropriate DNA loci for low taxonomic level studies of *Dendrobium* species^5, 6, 16^. However, due to limited loci or taxes sampled, some conclusions made in these studies are inconsistent. The mutational hotspots from plastid genome are the most commonly used tool for low-level phylogeographic and phylogenetic studies of plants^38–40^. Although in many *Dendrobium* studies, they are supplemented with nrDNA ITS sequences, it is still difficult to obtain sufficient informative sites. Their unavailability makes it very challenging to resolve the phylogenetic relationships among several unplaced species (i.e., *D*. *capillipes*, *D*. *trigonopus*) and closely related species^3, 41^, and to identify these species. Moreover, the most variable loci in one lineage may not be phylogenetically informative in other lineages. For instance, the loci *matK*, *rbcL* ^19^, and *psbA*-*trnH* ^20, 21^ are highly variable in angiosperms and have been proposed for DNA barcoding, yet they only showed moderate sequence divergences among all syntenic loci of *Dendrobium* plastomes tested in this study (Fig. 2, Table S3). Therefore, it is necessary to make a cautious evaluation of specific genetic markers for *Dendrobium* species.

By comparing 92 syntenic intergenic and intronic loci from 25 *Dendrobium* plastomes, our analyses revealed that the top ten hotspots listed in Fig. 2 were the fastest evolving loci, which may be used for the phylogenetic study and identification of *Dendrobium* species. Among these top ten loci, four (*psbB*-*psbT*, *rpl16*-*rps3*, *trnR*-*atpA*, and *trnL* intron) are reported as mutational hotspots for the first time in this study. The other six loci have been documented in previous studies, of which four (*ndhF*-*rpl32*, *rpl32*-*trnL*, *rps16*-*trnQ*, and *trnE*-*trnT*) are located in three most variable plastome regions—*ccsA* to *ndhF*, *matK* to *3*′ *trnG*, and *rpoB* to *psbD* ^42^—and the rest two (*trnT*-*trnL* and *clpB*-*psbB*) also have been considered hotspots for orchid species within *Cymbidium* and *Phalaenopsis* genera^25, 42^. This finding is in good agreement with the view proposed by Shaw *et al*.^42^ that although the top mutational hotspots are diversified in different lineages, some highly variable loci might remain unchanged in all angiosperm lineages.

Regarding 17 plastid introns, all except *trnL*-*UAA* intron belong to self-splicing group II introns^31^; none of them ranked in the top-ten mutational hotspots in our study. Compared to intergenic spacers, the group II introns had lower evolving rates, which could be explained by that their mutations may be constrained by their function in maintaining their secondary structural features, which are important for a proper splicing^43–45^. However, eight of these introns contain polymorphic SSRs (Table 3). Considering the functional importance of their secondary structural features, we surmise that the polymorphic SSRs might play a role in maintaining the secondary structures of group II introns.

The subject—“which hotspot and how many hotspots should be used”—has been debated for a long time^46–49^. Multiple solutions have been put forth in terms of the hotspot region and the number of hotspots in the combination, but no clear consensus result has yet emerged. For example, in *Dendrobium*, Singh *et al*.^50^ found that the DNA barcode based on three loci, *matK*, *rpoB*, and *rpoC1* could indentify the maximum number of *Dendrobium* species^50^; Xu *et al*.^6^ recommended utilizing the combination of ITS + *matK* as a core DNA barcode^6^; and ITS, *rbcL*, *matK*, *trnH*-*psbA*, and *trnL intron*/*trnL-trnF* were used to resolve the phylogenetic relationship of *Dendrobium* in the studies of Xiang *et al*.^3, 41^. More recently, Shaw *et al*.^42^ concluded that at least four and up to eight of the most variable hotspots will likely access the majority of the low-level discriminating power of the plastome depending on the lineage of interest^42^. Based on the results of the current research, we recommend that the combination of five hotspots—*trnT*-*trnL*, *rpl32*-*trnL*, *clpP*-*psbB*, *trnL intron*, and *rps16*-*trnQ*—should be used in *Dendrobium* studies due to three reasons. Firstly, the phylogenetic tree based on this combination showed a strong discriminating ability (nearly all nodes BS value >75%) for *Dendrobium* species (Fig. 4). Secondly, five hotspots are necessary to capture the species resolution power of *Dendrobium* plastome. Empirical data analyses have revealed that greatly increasing the number of hotspots will not improve species-level discrimination because of a “performance plateau”^49^. This “performance plateau” was also observed in our study, as manifested by that the mean BS value only slightly increased with more than five hotspots combined. Thirdly, the combination of *trnT*-*trnL*, *rpl32*-*trnL*, *clpP*-*psbB*, *trnL intron*, and *rps16*-*trnQ* contains the lowest number of hotspots while ranking among the top ten combinations that yielded highest BS values (Table S5); hence, it is cost effective to apply this hotspot combination to the phylogenetic and identification studies of *Dendrobium*.

## Methods

### Plant materials and DNA extraction

Two grams of fresh leaves were harvested from an individual plant of each tested *Dendrobium* species (Table 1) grown in the greenhouse of Nanjing Normal University. Total genomic DNA was isolated from the leaves using the DNeasy Plant Mini Kit (Qiagen, Germany) according to the manufacturer’s instructions. The DNA quality was examined by using a NanoDrop 8000 Spectrophotometer (Thermo Scientific, Wilmington, DE). DNA samples with concentration >300 ng/μl, A260/A280 = 1.8–2.0, and A260/A230 >1.7 were used for sequencing.

### Plastome sequencing, assembly, and annotation

The total DNA of each tested *Dendrobium* species was sequenced with an Illumina Hiseq4000 sequencer at 1 Gene, Hangzhou (Hangzhou, China). Approximately 8.75 Gb of 150 bp pair-end reads was yielded for each species; the raw reads were trimmed under the threshold with an error probability <0.05 and then *de*-*novo* assembled on CLC Genomics Workbench 6.0.1 (CLC Bio, Aarhus, Denmark). Contigs >30× sequencing depths were collected for reference-based assembly. The plastome of *D*. *officinale* (NC_024019) served as a reference sequence. The four junctions between LSC/SSC and IRs were confirmed by PCR amplification using specific primers. Plastome annotation was performed using DOGMA^51^ and tRNAscan-SE 1.21^52^. The exact boundaries of annotated genes were confirmed by aligning them with the corresponding orthologs from other *Dendrobium* species.

### Identification of InDels

Sequences of large single copy (LSC), small single copy (SSC), inverted repeat (IR) regions, and retained NDH gene residues from each tested *Dendrobium* plastome were aligned with reference sequences from the plastome of *D*. *officinale* according to the MAFFT program^53^. InDel events and lengths were counted and determined with DnaSP v5^54^.

### Estimates of sequence variability

To assess sequence variability (SV) among plastomes of *Dendrobium* species, firstly, we retrieved the sequences of intergenic and intronic loci from 25 newly sequenced plastomes. The loci that are flanked by the same genes/exons were identified as syntenic, while the loci smaller than 150 bp were discarded. Secondly, we complied 325 pairs of the 25 *Dendrobium* plastomes, and aligned the sequences of the syntenic loci for each pair by using MUSCLE^55^ with the “Refining” option implemented in Mega 5.2^56^. The gaps located at the 5′- and 3′-ends of alignments were excluded. DnaSP v5 was employed to count the numbers of pairwise mutations and InDel events. SV was calculated according to the method of Shaw *et al*.^42^: SV = (The number of nucleotide mutations + the number of InDel events)/(the number of conserved sites + the number of nucleotide mutations + the number of InDel events) × 100%. Finally, we calculated the average SV of each syntenic locus.

### Counts of SSR elements

SSR (simple sequence repeat) elements located in the syntenic loci were detected using GMATo according to the criteria that the “Mini-length” for mono-nucleotide and multi-nucleotide SSRs were set to be 8 and 5 units, respectively^57^.

### Phylogenetic analysis

The sequences of top ten hotspots (*psbB*-*psbT*, *ndhF*-*rpl32*, *trnT*-*trnL*, *rpl32*-*trnL*, *clpB*-*psbB*, *trnL* intron, *rpl16*-*rps3*, *trnE*-*trnT*, *trnR*-*atpA*, and *rps16*-*trnQ*) were retrieved from plastomes of 30 *Dendrobium* species. Sequence alignments of these loci were separately performed using MUSCLE, and then concatenated into 1023 combinations using SequenceMatrix 1.8^58^ (Table S4). ML trees were constructed using RAxML 8.0.2^59^, with *Phalaenopsis aphrodite* (NC_007499), *Phalaenopsis equestris* (NC_017609), and *Phalaenopsis* (*hybrid cultivar*) (NC_025593) designated as outgroups. For the maximum likelihood tree analysis, a GTRGAMMA model was employed, and supporting values of tree nodes were estimated from 1,000 bootstrap replicates.

### Statistical analyses

Statistical analyses were performed by using SPSS Statistics 20.0.

## Electronic supplementary material

Supplementary information
