# Supplementary material for: Comparative analysis of Dendrobium plastomes and utility of plastomic mutational hotspots
Source: Sci Rep. 2017 May 18;7:2073. doi: 10.1038/s41598-017-02252-8 (PMC5437043; doi:10.1038/s41598-017-02252-8)
Supplement: Supplementary file 1 — Supplementary information [file 41598_2017_2252_MOESM1_ESM.pdf]

Comparative analysis of *Dendrobium* plastomes and utility of plastomic mutational hotspots

Niu Zhitao, Zhu Shuying, Pan Jiajia, Li Ludan, Sun Jing, Ding Xiaoyu*

College of Life Sciences, Nanjing Normal University, Nanjing, China
*Correspondence (dingxynj@263.net)

Figure S1 Plastomes of *Dendrobium* species and independent loss of their NDH genes. (a) The plastome of *Dendrobium* species. Only the plastome of *D. devonianum* is shown; the genes outside and inside the circle are transcribed clockwise and counterclockwise, respectively. (b) The independent loss of NDH genes. The sequence of eleven NDH genes of *Dendrobium* species were compared to *Cypripedium formosanum, Nicotiana tabacum and Arabidopsis thaliana*. The white box indictaed the gene with full reading frame, the yellow box indicated the gene were pseudogenized with frame-shift or short InDels, the blue box indicated the gene were truncated with significant InDels, the red box indicated the gene were completely lost.

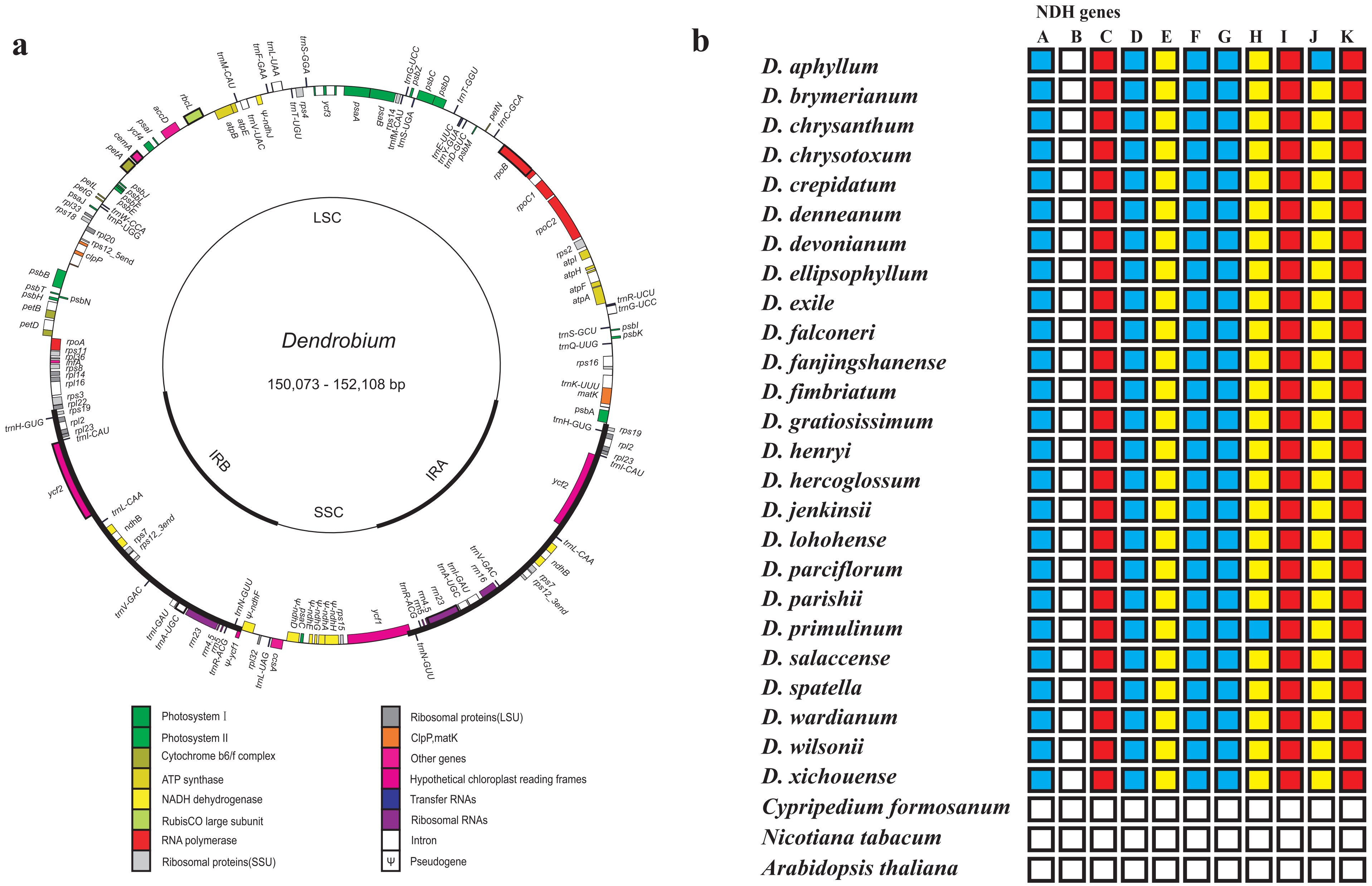

Table S1 Retained NDH gene length (bp) among *Dendrobium* species

Species Name	*ndhA*	*ndhB*	*ndhC*	*ndhD*	*ndhE*	*ndhF*	*ndhG*	*ndhH*	*ndhI*	*ndhJ*	*ndhK*	total bp of all ndh genes
D. aphyllum	552	1533	0	985	282	1029	213	1203	0	382	0	6179
D. brymerianum	552	1533	0	1039	282	1062	213	1179	0	450	0	6310
D. chrysanthum	552	1533	0	1038	282	1062	213	1179	0	450	0	6309
D. chrysotoxum	552	1533	0	1038	282	1065	213	1179	0	450	0	6312
D. crepidatum	552	1533	0	1038	282	1062	213	1179	0	450	0	6309
D. denneanum	552	1533	0	1053	282	1062	213	1179	0	450	0	6324
D. devonianum	552	1533	0	1038	282	1062	213	1179	0	450	0	6309
D. ellipsophyllum	552	1533	0	1038	282	1062	213	1179	0	450	0	6309
D. exile	553	1533	0	950	282	1028	210	1180	0	433	0	6169
D. falconeri	552	1533	0	1038	282	1062	213	1179	0	450	0	6309
D. fanjingshanense	552	1533	0	1041	282	1061	213	1185	0	450	0	6317
D. fimbriatum	552	1533	0	1065	282	1062	213	1179	0	450	0	6336
D. gratiosissimum	560	1533	0	1029	279	1071	204	1174	0	450	0	6300
D. henryi	552	1533	0	1038	282	1062	213	1179	0	450	0	6309
D. hercoglossum	552	1533	0	1040	282	1039	213	1176	0	450	0	6285
D. huoshanense	0	1533	0	915	303	291	204	0	0	450	0	3696
D. jenkinsii	552	1533	0	1050	282	1068	210	1179	0	449	0	6323
D. loddigesii	1086	1533	0	1489	306	0	0	1173	487	0	0	6074
D. lohohense	553	1533	0	950	282	1028	210	1180	0	433	0	6169
D. moniliforme	0	1533	0	919	296	291	204	0	0	444	0	3687
D. nobile	597	1533	0	1033	316	0	198	1182	0	452	0	5311
D. officinale	552	1533	0	1046	285	1010	198	1183	0	432	0	6239
D. parciflorum	444	1533	0	1046	270	72	220	1081	0	433	0	5099
D. parishii	552	1533	0	1031	282	1071	213	1177	0	452	0	6311

D. primulinum	554	1533	0	1031	282	1069	197	927	0	432	0		6025
D. salaccense	552	1533	0	1038	282	1062	210	1179	0	444	0		6300
D. spatella	552	1536	0	1099	282	1017	219	1158	0	427	0		6290
D. wardianum	560	1533	0	1029	279	1071	204	1174	0	450	0		6300
D. wilsonii	558	1533	0	1040	282	1038	213	1179	0	450	0		6293
D. xichouense	552	1533	0	941	279	1056	204	1179	0	450	0		6194

Table S2 InDel and plastomes changed lengths (bp) among *Dendrobium* species

Species	Whole plastome changed lengths	NDH gene-related InDel change	NDH gene-unrelated InDel change
D. aphyllum	697	60	667
D. brymerianum	391	71	323
D. chrysanthum	431	70	359
D. chrysotoxum	490	73	414
D. crepidatum	504	70	397
D. denneanum	656	85	555
D. devonianum	276	70	180
D. ellipsophyllum	195	70	143
D. exile	927	70	887
D. falconeri	331	70	253
D. fanjingshanense	113	78	47
D. fimbriatum	548	97	444
D. gratiosissimum	392	61	323
D. henryi	371	70	316
D. hercoglossum	282	46	269
D. huoshanense	967	900	160
D. jenkinsii	504	84	407
D. loddigesii	2510	857	4374
D. lohohense	409	70	333
D. moniliforme	3557	2552	690
D. nobile	1594	928	374
D. parciflorum	2148	1140	982
D. parishii	532	72	457
D. primulinum	1454	214	1117
D. salaccense	1117	61	1016
D. spatella	392	51	371
D. wardianum	392	61	323
D. wilsonii	141	54	111
D. xichouense	169	45	119

"NDH gene-related InDel change": the difference between the insertions and deletions in NDH genes

"NDH gene-unrelated InDel change": the difference between the insertions and deletions in plastomes except for NDH genes

Table S3 Sequence variability (%) among 92 syntenic intergenic and intronic loci

Locus	Location	GC content (%)	Sequence variability (%)	Stv.
3'rps12 intron	IR	39.29	0.192918193	0.15523
3'rps12-trnV	IR	38.95	0.995425932	0.567666
ndhB intron	IR	38.91	0.392145228	0.300282
ndhB-rps7	IR	35.56	0.381934466	0.465517
rpl2 intron	IR	40.67	0.227263221	0.222534
rpl22-rps19	IR	28.88	2.623700544	1.638504
rpl23-trnI	IR	34.76	0.402439024	0.447509
rrn16-trnI	IR	50.00	0.943521595	1.111164
rrn4.5-rrn5	IR	46.92	0.565143521	0.871193
rrn5-trnR	IR	41.63	0.773098177	0.69796
trnA intron	IR	51.13	0.179512149	0.171729
trnI intron	IR	49.63	0.305501098	0.159809
trnL-ndhB	IR	37.63	0.381149205	0.294999
trnN-ycf1	IR	37.46	0.418619248	0.420074
trnR-trnN	IR	42.70	1.390598508	0.692618
trnV-rrn16	IR	46.93	0.32748538	0.357287
ycf2-trnL	IR	43.50	0.278443797	0.224071
3'trnK-matK	LSC	29.29	2.826239233	1.29109
accD-psaI	LSC	29.49	2.837525758	1.247444
atpB-rbcL	LSC	24.08	2.952553991	1.079744
atpF intron	LSC	30.85	1.890558502	0.769145
atpH-atpI	LSC	31.10	2.395060702	1.15313
atpI-rps2	LSC	24.74	2.354222219	1.095582
cemA-petA	LSC	29.70	0.94017094	0.718242
clpP intron1	LSC	30.81	2.462010686	1.139715
clpP intron2	LSC	32.16	1.266410847	0.641879
clpP-psbB	LSC	25.35	4.223453084	1.729745
matK-5'trnK	LSC	29.24	2.904770558	1.19552
ndhJ-trnV	LSC	34.92	2.632497899	1.295169
petA-psbJ	LSC	29.76	2.514591227	1.212084
petB intron	LSC	34.64	1.215270438	0.609665
petB-petD	LSC	30.98	1.691274971	0.809018
petD intron	LSC	34.46	1.296685185	0.554268
petL-petG	LSC	30.32	1.569148936	0.911899
petN-psbM	LSC	26.75	3.4785656	1.127212
psaA-ycf3	LSC	30.31	2.165682481	0.927632
psaI-ycf4	LSC	32.40	2.306143167	1.066414
psaJ-rpl33	LSC	27.71	3.128463252	1.210501
psbA-trnK	LSC	28.46	2.778466796	1.725679
psbB-psbT	LSC	18.42	8.614013158	2.950269
psbE-petL	LSC	29.57	2.178478564	0.738383
psbH-petB	LSC	26.29	1.755329552	1.181128

psbK-psbI	LSC	26.41	3.278836606	1.593692
psbM-trnD	LSC	31.15	2.464240105	1.444846
psbZ-trnG	LSC	32.18	2.072914352	1.177721
rbcL-accD	LSC	30.79	1.896220234	0.855359
rpl16 intron	LSC	28.30	3.300143048	1.101229
rpl16-rps3	LSC	22.99	4.049097356	1.525526
rpl20-5'rps12	LSC	34.76	1.392737904	0.524599
rpl33-rps18	LSC	28.68	2.248967404	1.34457
rpoB-trnC	LSC	30.79	2.340778226	1.181908
rpoC1 intron	LSC	37.55	1.336046171	0.555207
rpoC2-rpoC1	LSC	36.80	2.1289257	1.516751
rps11-rpl36	LSC	28.44	1.047312494	0.841387
rps16 intron	LSC	28.30	3.544680768	1.276199
rps16-trnQ	LSC	24.93	3.669888068	1.153971
rps18-rpl20	LSC	30.89	2.749217069	1.110407
rps2-rpoC2	LSC	32.02	2.555522365	1.067519
rps4-trnT	LSC	29.60	2.063555926	0.958531
rps8-rpl14	LSC	22.17	3.580317962	1.464192
trnC-petN	LSC	33.03	2.84393452	1.268326
trnD-trnY	LSC	32.17	1.711271335	0.816197
trnE-trnT	LSC	29.89	3.943767042	1.478844
trnfM-rps14	LSC	36.42	1.274020397	1.02528
trnF-ndhJ	LSC	33.16	2.036123154	1.159421
trnF-trnV	LSC	32.52	2.508478579	1.082953
trnG-trnfM	LSC	31.32	2.586139111	1.708625
trnG-trnG	LSC	33.58	1.248701053	0.736716
trnK-rps16	LSC	28.24	2.705142186	1.425158
trnL intron	LSC	28.91	4.201183819	1.314412
trnL-trnF	LSC	31.31	3.32391715	3.020102
trnM-atpE	LSC	29.53	1.919108358	1.233468
trnP-psaJ	LSC	28.04	2.081251225	1.233525
trnQ-psbK	LSC	26.22	1.753560163	0.850836
trnR-atpA	LSC	24.55	3.791823284	1.448905
trnS-psbZ	LSC	31.91	1.591271424	0.705885
trnS-rps4	LSC	30.98	2.919682134	1.868186
trnS-trnG	LSC	26.30	3.522375199	1.357228
trnT-psbD	LSC	32.97	1.746115359	0.774162
trnT-trnL	LSC	24.44	4.737204506	1.491538
trnV intron	LSC	38.67	0.629344151	0.46327
trnV-trnM	LSC	32.08	2.161525227	1.305705
trnW-trnP	LSC	31.63	2.981308263	1.733582
ycf3 intron1	LSC	36.28	1.114261545	0.3979
ycf3 intron2	LSC	33.77	0.810722519	0.410755
ycf3-trnS	LSC	32.37	1.23087239	0.744996

ycf4-cemA	LSC	32.21	2.543172824	1.262342
ccsA-ndhD	SSC	27.31	3.267236439	2.101529
ndhF-rpl32	SSC	20.25	4.757562882	2.146326
rpl32-trnL	SSC	24.41	4.490669637	2.019691
rps15-ycf1	SSC	25.63	2.349051205	1.081176

Table S4 Combinations based on top-ten mutational hotspots and their bootstrap (BS) values

No.	Hotspot combination		Length	SV	BS
1	*psbB-psbT*		679	8.61	50.13
2	*ndhF-rpl32*		396	4.76	43.90
3	*trnT-trnL*		839	4.74	43.84
4	*rpl32-trnL*		870	4.49	54.06
5	*clpP-psbB*		853	4.22	42.32
6	*trnL intron*		973	4.20	56.19
7	*rpl16-rps3*		205	4.05	37.35
8	*trnE-trnT*		936	3.94	34.29
9	*trnR-atpA*		231	3.79	34.52
10	*rps16-trnQ*		1204	3.67	57.06
11	*psbB-psbT*	*ndhF-rpl32*	1075	112.61	34.16
12	*psbB-psbT*	*trnT-trnL*	1518	104.38	52.42
13	*psbB-psbT*	*rpl32-trnL*	1549	105.88	65.48
14	*psbB-psbT*	*clpP-psbB*	1532	95.69	52.94
15	*psbB-psbT*	*trnL intron*	1652	93.98	63.77
16	*psbB-psbT*	*rpl16-rps3*	884	98.66	58.39
17	*psbB-psbT*	*trnE-trnT*	1615	104.52	43.55
18	*psbB-psbT*	*trnR-atpA*	910	99.22	50.29
19	*psbB-psbT*	*rps16-trnQ*	1883	103.94	62.23
20	*ndhF-rpl32*	*trnT-trnL*	1235	92.29	62.16
21	*ndhF-rpl32*	*rpl32-trnL*	1266	93.79	54.55
22	*ndhF-rpl32*	*clpP-psbB*	1249	83.60	61.35
23	*ndhF-rpl32*	*trnL intron*	1369	81.89	60.52
24	*ndhF-rpl32*	*rpl16-rps3*	601	86.57	55.97
25	*ndhF-rpl32*	*trnE-trnT*	1332	92.43	50.65

26	*ndhF-rpl32*	*trnR-atpA*	627	87.13	46.13
27	*ndhF-rpl32*	*rps16-trnQ*	1600	91.85	59.06
28	*trnT-trnL*	*rpl32-trnL*	1709	85.56	66.19
29	*trnT-trnL*	*clpP-psbB*	1692	75.36	74.42
30	*trnT-trnL*	*trnL intron*	1812	73.65	64.16
31	*trnT-trnL*	*rpl16-rps3*	1044	78.33	57.81
32	*trnT-trnL*	*trnE-trnT*	1775	84.19	61.06
33	*trnT-trnL*	*trnR-atpA*	1070	78.90	60.45
34	*trnT-trnL*	*rps16-trnQ*	2043	83.61	73.42
35	*rpl32-trnL*	*clpP-psbB*	1723	76.86	61.23
36	*rpl32-trnL*	*trnL intron*	1843	75.16	51.13
37	*rpl32-trnL*	*rpl16-rps3*	1075	79.84	44.19
38	*rpl32-trnL*	*trnE-trnT*	1806	85.70	47.68
39	*rpl32-trnL*	*trnR-atpA*	1101	80.40	65.71
40	*rpl32-trnL*	*rps16-trnQ*	2074	85.12	62.06
41	*clpP-psbB*	*trnL intron*	1826	64.96	59.84
42	*clpP-psbB*	*rpl16-rps3*	1058	69.64	60.42
43	*clpP-psbB*	*trnE-trnT*	1789	75.50	68.94
44	*clpP-psbB*	*trnR-atpA*	1084	70.20	46.61
45	*clpP-psbB*	*rps16-trnQ*	2057	74.92	40.19
46	*trnL intron*	*rpl16-rps3*	1178	67.93	42.81
47	*trnL intron*	*trnE-trnT*	1909	73.79	63.48
48	*trnL intron*	*trnR-atpA*	1204	68.50	38.97
49	*trnL intron*	*rps16-trnQ*	2177	73.21	55.26
50	*rpl16-rps3*	*trnE-trnT*	1141	78.47	63.06
51	*rpl16-rps3*	*trnR-atpA*	436	73.18	72.35
52	*rpl16-rps3*	*rps16-trnQ*	1409	77.89	58.39

53	*trnE-trnT*	*trnR-atpA*		1167	79.04	41.77
54	*trnE-trnT*	*rps16-trnQ*		2140	83.75	56.00
55	*trnR-atpA*	*rps16-trnQ*		1435	78.46	49.32
56	*psbB-psbT*	*ndhF-rpl32*	*trnT-trnL*	1914	154.64	66.13
57	*psbB-psbT*	*ndhF-rpl32*	*rpl32-trnL*	1945	156.15	66.61
58	*psbB-psbT*	*ndhF-rpl32*	*clpP-psbB*	1928	145.95	64.90
59	*psbB-psbT*	*ndhF-rpl32*	*trnL intron*	2048	144.24	78.48
60	*psbB-psbT*	*ndhF-rpl32*	*rpl16-rps3*	1280	148.92	44.87
61	*psbB-psbT*	*ndhF-rpl32*	*trnE-trnT*	2011	154.78	56.19
62	*psbB-psbT*	*ndhF-rpl32*	*trnR-atpA*	1306	149.49	68.13
63	*psbB-psbT*	*ndhF-rpl32*	*rps16-trnQ*	2279	154.20	44.45
64	*psbB-psbT*	*trnT-trnL*	*rpl32-trnL*	2388	147.91	63.03
65	*psbB-psbT*	*trnT-trnL*	*clpP-psbB*	2371	137.71	66.81
66	*psbB-psbT*	*trnT-trnL*	*trnL intron*	2491	136.00	71.77
67	*psbB-psbT*	*trnT-trnL*	*rpl16-rps3*	1723	140.69	77.65
68	*psbB-psbT*	*trnT-trnL*	*trnE-trnT*	2454	146.54	69.23
69	*psbB-psbT*	*trnT-trnL*	*trnR-atpA*	1749	141.25	57.94
70	*psbB-psbT*	*trnT-trnL*	*rps16-trnQ*	2722	145.96	67.42
71	*psbB-psbT*	*rpl32-trnL*	*clpP-psbB*	2402	139.22	74.00
72	*psbB-psbT*	*rpl32-trnL*	*trnL intron*	2522	137.51	63.00
73	*psbB-psbT*	*rpl32-trnL*	*rpl16-rps3*	1754	142.19	60.45
74	*psbB-psbT*	*rpl32-trnL*	*trnE-trnT*	2485	148.05	56.29
75	*psbB-psbT*	*rpl32-trnL*	*trnR-atpA*	1780	142.76	56.26
76	*psbB-psbT*	*rpl32-trnL*	*rps16-trnQ*	2753	147.47	65.94
77	*psbB-psbT*	*clpP-psbB*	*trnL intron*	2505	127.31	64.90
78	*psbB-psbT*	*clpP-psbB*	*rpl16-rps3*	1737	132.00	66.65
79	*psbB-psbT*	*clpP-psbB*	*trnE-trnT*	2468	137.85	61.84

80	*psbB-psbT*	*clpP-psbB*	*trnR-atpA*	1763	132.56	69.03
81	*psbB-psbT*	*clpP-psbB*	*rps16-trnQ*	2736	137.27	54.84
82	*psbB-psbT*	*trnL intron*	*rpl16-rps3*	1857	130.29	58.55
83	*psbB-psbT*	*trnL intron*	*trnE-trnT*	2588	136.14	61.39
84	*psbB-psbT*	*trnL intron*	*trnR-atpA*	1883	130.85	50.90
85	*psbB-psbT*	*trnL intron*	*rps16-trnQ*	2856	135.56	61.81
86	*psbB-psbT*	*rpl16-rps3*	*trnE-trnT*	1820	140.83	60.03
87	*psbB-psbT*	*rpl16-rps3*	*trnR-atpA*	1115	135.53	72.06
88	*psbB-psbT*	*rpl16-rps3*	*rps16-trnQ*	2088	140.25	72.84
89	*psbB-psbT*	*trnE-trnT*	*trnR-atpA*	1846	141.39	62.87
90	*psbB-psbT*	*trnE-trnT*	*rps16-trnQ*	2819	146.10	70.65
91	*psbB-psbT*	*trnR-atpA*	*rps16-trnQ*	2114	140.81	75.77
92	*ndhF-rpl32*	*trnT-trnL*	*rpl32-trnL*	2105	135.82	73.32
93	*ndhF-rpl32*	*trnT-trnL*	*clpP-psbB*	2088	125.62	70.23
94	*ndhF-rpl32*	*trnT-trnL*	*trnL intron*	2208	123.91	74.45
95	*ndhF-rpl32*	*trnT-trnL*	*rpl16-rps3*	1440	128.60	77.65
96	*ndhF-rpl32*	*trnT-trnL*	*trnE-trnT*	2171	134.45	57.71
97	*ndhF-rpl32*	*trnT-trnL*	*trnR-atpA*	1466	129.16	64.26
98	*ndhF-rpl32*	*trnT-trnL*	*rps16-trnQ*	2439	133.87	74.32
99	*ndhF-rpl32*	*rpl32-trnL*	*clpP-psbB*	2119	127.13	57.19
100	*ndhF-rpl32*	*rpl32-trnL*	*trnL intron*	2239	125.42	67.32
101	*ndhF-rpl32*	*rpl32-trnL*	*rpl16-rps3*	1471	130.10	76.58
102	*ndhF-rpl32*	*rpl32-trnL*	*trnE-trnT*	2202	135.96	65.03
103	*ndhF-rpl32*	*rpl32-trnL*	*trnR-atpA*	1497	130.66	65.06
104	*ndhF-rpl32*	*rpl32-trnL*	*rps16-trnQ*	2470	135.38	63.13
105	*ndhF-rpl32*	*clpP-psbB*	*trnL intron*	2222	115.22	74.10
106	*ndhF-rpl32*	*clpP-psbB*	*rpl16-rps3*	1454	119.90	49.52

107	*ndhF-rpl32*	*clpP-psbB*	*trnE-trnT*	2185	125.76	59.32
108	*ndhF-rpl32*	*clpP-psbB*	*trnR-atpA*	1480	120.47	68.90
109	*ndhF-rpl32*	*clpP-psbB*	*rps16-trnQ*	2453	125.18	51.68
110	*ndhF-rpl32*	*trnL intron*	*rpl16-rps3*	1574	118.20	63.45
111	*ndhF-rpl32*	*trnL intron*	*trnE-trnT*	2305	124.05	67.77
112	*ndhF-rpl32*	*trnL intron*	*trnR-atpA*	1600	118.76	61.03
113	*ndhF-rpl32*	*trnL intron*	*rps16-trnQ*	2573	123.47	62.74
114	*ndhF-rpl32*	*rpl16-rps3*	*trnE-trnT*	1537	128.74	71.03
115	*ndhF-rpl32*	*rpl16-rps3*	*trnR-atpA*	832	123.44	55.39
116	*ndhF-rpl32*	*rpl16-rps3*	*rps16-trnQ*	1805	128.16	66.68
117	*ndhF-rpl32*	*trnE-trnT*	*trnR-atpA*	1563	129.30	73.94
118	*ndhF-rpl32*	*trnE-trnT*	*rps16-trnQ*	2536	134.01	42.84
119	*ndhF-rpl32*	*trnR-atpA*	*rps16-trnQ*	1831	128.72	56.48
120	*trnT-trnL*	*rpl32-trnL*	*clpP-psbB*	2562	118.89	66.65
121	*trnT-trnL*	*rpl32-trnL*	*trnL intron*	2682	117.18	56.71
122	*trnT-trnL*	*rpl32-trnL*	*rpl16-rps3*	1914	121.87	64.45
123	*trnT-trnL*	*rpl32-trnL*	*trnE-trnT*	2645	127.72	54.32
124	*trnT-trnL*	*rpl32-trnL*	*trnR-atpA*	1940	122.43	43.84
125	*trnT-trnL*	*rpl32-trnL*	*rps16-trnQ*	2913	127.14	72.97
126	*trnT-trnL*	*clpP-psbB*	*trnL intron*	2665	106.98	43.45
127	*trnT-trnL*	*clpP-psbB*	*rpl16-rps3*	1897	111.67	50.61
128	*trnT-trnL*	*clpP-psbB*	*trnE-trnT*	2628	117.52	38.35
129	*trnT-trnL*	*clpP-psbB*	*trnR-atpA*	1923	112.23	57.74
130	*trnT-trnL*	*clpP-psbB*	*rps16-trnQ*	2896	116.94	70.55
131	*trnT-trnL*	*trnL intron*	*rpl16-rps3*	2017	109.96	63.97
132	*trnT-trnL*	*trnL intron*	*trnE-trnT*	2748	115.81	75.39
133	*trnT-trnL*	*trnL intron*	*trnR-atpA*	2043	110.52	62.74

134	*trnT-trnL*	*trnL intron*	*rps16-trnQ*	3016	115.23	63.65
135	*trnT-trnL*	*rpl16-rps3*	*trnE-trnT*	1980	120.50	62.65
136	*trnT-trnL*	*rpl16-rps3*	*trnR-atpA*	1275	115.21	70.10
137	*trnT-trnL*	*rpl16-rps3*	*rps16-trnQ*	2248	119.92	54.06
138	*trnT-trnL*	*trnE-trnT*	*trnR-atpA*	2006	121.06	77.26
139	*trnT-trnL*	*trnR-atpA*	*rps16-trnQ*	2274	120.48	45.48
140	*trnT-trnL*	*trnR-atpA*	*rps16-trnQ*	2274	120.48	53.77
141	*rpl32-trnL*	*clpP-psbB*	*trnL intron*	2696	108.49	61.19
142	*rpl32-trnL*	*clpP-psbB*	*rpl16-rps3*	1928	113.17	65.00
143	*rpl32-trnL*	*clpP-psbB*	*trnE-trnT*	2659	119.03	76.06
144	*rpl32-trnL*	*clpP-psbB*	*trnR-atpA*	1954	113.74	50.10
145	*rpl32-trnL*	*clpP-psbB*	*rps16-trnQ*	2927	118.45	55.61
146	*rpl32-trnL*	*trnL intron*	*rpl16-rps3*	2048	111.47	52.39
147	*rpl32-trnL*	*trnL intron*	*trnE-trnT*	2779	117.32	51.77
148	*rpl32-trnL*	*trnL intron*	*trnR-atpA*	2074	112.03	73.35
149	*rpl32-trnL*	*trnL intron*	*rps16-trnQ*	3047	116.74	57.45
150	*rpl32-trnL*	*rpl16-rps3*	*trnE-trnT*	2011	122.01	75.74
151	*rpl32-trnL*	*rpl16-rps3*	*trnR-atpA*	1306	116.71	79.29
152	*rpl32-trnL*	*rpl16-rps3*	*rps16-trnQ*	2279	121.43	54.23
153	*rpl32-trnL*	*trnE-trnT*	*trnR-atpA*	2037	122.57	61.35
154	*rpl32-trnL*	*trnE-trnT*	*rps16-trnQ*	3010	127.28	48.68
155	*rpl32-trnL*	*trnR-atpA*	*rps16-trnQ*	2305	121.99	44.87
156	*clpP-psbB*	*trnL intron*	*rpl16-rps3*	2031	101.27	57.97
157	*clpP-psbB*	*trnL intron*	*trnE-trnT*	2762	107.12	61.19
158	*clpP-psbB*	*trnL intron*	*trnR-atpA*	2057	101.83	73.23
159	*clpP-psbB*	*trnL intron*	*rps16-trnQ*	3030	106.54	58.90
160	*clpP-psbB*	*rpl16-rps3*	*trnE-trnT*	1994	111.81	63.61

161	*clpP-psbB*	*rpl16-rps3*	*trnR-atpA*		1289	106.51	62.16
162	*clpP-psbB*	*rpl16-rps3*	*rps16-trnQ*		2262	111.23	54.74
163	*clpP-psbB*	*trnE-trnT*	*trnR-atpA*		2020	112.37	60.61
164	*clpP-psbB*	*trnE-trnT*	*rps16-trnQ*		2993	117.08	63.87
165	*clpP-psbB*	*trnR-atpA*	*rps16-trnQ*		2288	111.79	73.87
166	*trnL intron*	*rpl16-rps3*	*trnE-trnT*		2114	110.10	74.19
167	*trnL intron*	*rpl16-rps3*	*trnR-atpA*		1409	104.81	73.10
168	*trnL intron*	*rpl16-rps3*	*rps16-trnQ*		2382	109.52	63.74
169	*trnL intron*	*trnE-trnT*	*trnR-atpA*		2140	110.66	72.42
170	*trnL intron*	*trnE-trnT*	*rps16-trnQ*		3113	115.37	78.23
171	*trnL intron*	*trnR-atpA*	*rps16-trnQ*		2408	110.08	66.61
172	*rpl16-rps3*	*trnE-trnT*	*trnR-atpA*		1372	115.35	64.00
173	*rpl16-rps3*	*trnE-trnT*	*rps16-trnQ*		2345	120.06	52.58
174	*rpl16-rps3*	*trnR-atpA*	*rps16-trnQ*		1640	114.77	61.87
175	*trnE-trnT*	*trnR-atpA*	*rps16-trnQ*		2371	120.62	69.52
176	*psbB-psbT*	*ndhF-rpl32*	*trnT-trnL*	*rpl32-trnL*	2784	198.17	69.10
177	*psbB-psbT*	*ndhF-rpl32*	*trnT-trnL*	*clpP-psbB*	2767	187.97	63.48
178	*psbB-psbT*	*ndhF-rpl32*	*trnT-trnL*	*trnL intron*	2887	186.26	75.58
179	*psbB-psbT*	*ndhF-rpl32*	*trnT-trnL*	*rpl16-rps3*	2119	190.95	64.48
180	*psbB-psbT*	*ndhF-rpl32*	*trnT-trnL*	*trnE-trnT*	2850	196.80	63.52
181	*psbB-psbT*	*ndhF-rpl32*	*trnT-trnL*	*trnR-atpA*	2145	191.51	69.03
182	*psbB-psbT*	*ndhF-rpl32*	*trnT-trnL*	*rps16-trnQ*	3118	196.22	66.42
183	*psbB-psbT*	*ndhF-rpl32*	*rpl32-trnL*	*clpP-psbB*	2798	189.48	59.23
184	*psbB-psbT*	*ndhF-rpl32*	*rpl32-trnL*	*trnL intron*	2918	187.77	75.29
185	*psbB-psbT*	*ndhF-rpl32*	*rpl32-trnL*	*rpl16-rps3*	2150	192.46	58.39
186	*psbB-psbT*	*ndhF-rpl32*	*rpl32-trnL*	*trnE-trnT*	2881	198.31	62.06
187	*psbB-psbT*	*ndhF-rpl32*	*rpl32-trnL*	*trnR-atpA*	2176	193.02	59.87

188	*psbB-psbT*	*ndhF-rpl32*	*rpl32-trnL*	*rps16-trnQ*	3149	197.73	72.10
189	*psbB-psbT*	*ndhF-rpl32*	*clpP-psbB*	*trnL intron*	2901	177.57	77.81
190	*psbB-psbT*	*ndhF-rpl32*	*clpP-psbB*	*rpl16-rps3*	2133	182.26	53.68
191	*psbB-psbT*	*ndhF-rpl32*	*clpP-psbB*	*trnE-trnT*	2864	188.11	60.06
192	*psbB-psbT*	*ndhF-rpl32*	*clpP-psbB*	*trnR-atpA*	2159	182.82	48.77
193	*psbB-psbT*	*ndhF-rpl32*	*clpP-psbB*	*rps16-trnQ*	3132	187.53	62.87
194	*psbB-psbT*	*ndhF-rpl32*	*trnL intron*	*rpl16-rps3*	2253	180.55	78.35
195	*psbB-psbT*	*ndhF-rpl32*	*trnL intron*	*trnE-trnT*	2984	186.41	67.61
196	*psbB-psbT*	*ndhF-rpl32*	*trnL intron*	*trnR-atpA*	2279	181.11	77.19
197	*psbB-psbT*	*ndhF-rpl32*	*trnL intron*	*rps16-trnQ*	3252	185.82	76.26
198	*psbB-psbT*	*ndhF-rpl32*	*rpl16-rps3*	*trnE-trnT*	2216	191.09	54.35
199	*psbB-psbT*	*ndhF-rpl32*	*rpl16-rps3*	*trnR-atpA*	1511	185.80	47.13
200	*psbB-psbT*	*ndhF-rpl32*	*rpl16-rps3*	*rps16-trnQ*	2484	190.51	59.90
201	*psbB-psbT*	*ndhF-rpl32*	*trnE-trnT*	*trnR-atpA*	2242	191.65	56.16
202	*psbB-psbT*	*ndhF-rpl32*	*trnE-trnT*	*rps16-trnQ*	3215	196.36	66.32
203	*psbB-psbT*	*ndhF-rpl32*	*trnR-atpA*	*rps16-trnQ*	2510	191.07	55.77
204	*psbB-psbT*	*trnT-trnL*	*rpl32-trnL*	*clpP-psbB*	3241	181.24	68.58
205	*psbB-psbT*	*trnT-trnL*	*rpl32-trnL*	*trnL intron*	3361	179.53	76.61
206	*psbB-psbT*	*trnT-trnL*	*rpl32-trnL*	*rpl16-rps3*	2593	184.22	71.39
207	*psbB-psbT*	*trnT-trnL*	*rpl32-trnL*	*trnE-trnT*	3324	190.07	70.23
208	*psbB-psbT*	*trnT-trnL*	*rpl32-trnL*	*trnR-atpA*	2619	184.78	70.42
209	*psbB-psbT*	*trnT-trnL*	*rpl32-trnL*	*rps16-trnQ*	3592	189.49	70.52
210	*psbB-psbT*	*trnT-trnL*	*clpP-psbB*	*trnL intron*	3344	169.34	73.16
211	*psbB-psbT*	*trnT-trnL*	*clpP-psbB*	*rpl16-rps3*	2576	174.02	63.81
212	*psbB-psbT*	*trnT-trnL*	*clpP-psbB*	*trnE-trnT*	3307	179.88	63.87
213	*psbB-psbT*	*trnT-trnL*	*clpP-psbB*	*trnR-atpA*	2602	174.58	64.84
214	*psbB-psbT*	*trnT-trnL*	*clpP-psbB*	*rps16-trnQ*	3575	179.29	66.74

215	*psbB-psbT*	*trnT-trnL*	*trnL intron*	*rpl16-rps3*	2696	172.31	72.26
216	*psbB-psbT*	*trnT-trnL*	*trnL intron*	*trnE-trnT*	3427	178.17	75.03
217	*psbB-psbT*	*trnT-trnL*	*trnL intron*	*trnR-atpA*	2722	172.87	76.84
218	*psbB-psbT*	*trnT-trnL*	*trnL intron*	*rps16-trnQ*	3695	177.59	75.65
219	*psbB-psbT*	*trnT-trnL*	*rpl16-rps3*	*trnE-trnT*	2659	182.85	67.26
220	*psbB-psbT*	*trnT-trnL*	*rpl16-rps3*	*trnR-atpA*	1954	177.56	62.97
221	*psbB-psbT*	*trnT-trnL*	*rpl16-rps3*	*rps16-trnQ*	2927	182.27	65.55
222	*psbB-psbT*	*trnT-trnL*	*trnE-trnT*	*trnR-atpA*	2685	183.41	62.87
223	*psbB-psbT*	*trnT-trnL*	*trnE-trnT*	*rps16-trnQ*	3658	188.13	69.77
224	*psbB-psbT*	*trnT-trnL*	*trnR-atpA*	*rps16-trnQ*	2953	182.83	67.39
225	*psbB-psbT*	*rpl32-trnL*	*clpP-psbB*	*trnL intron*	3375	170.84	74.61
226	*psbB-psbT*	*rpl32-trnL*	*clpP-psbB*	*rpl16-rps3*	2607	175.53	55.90
227	*psbB-psbT*	*rpl32-trnL*	*clpP-psbB*	*trnE-trnT*	3338	181.38	60.06
228	*psbB-psbT*	*rpl32-trnL*	*clpP-psbB*	*trnR-atpA*	2633	176.09	52.81
229	*psbB-psbT*	*rpl32-trnL*	*clpP-psbB*	*rps16-trnQ*	3606	180.80	67.39
230	*psbB-psbT*	*rpl32-trnL*	*trnL intron*	*rpl16-rps3*	2727	173.82	76.58
231	*psbB-psbT*	*rpl32-trnL*	*trnL intron*	*trnE-trnT*	3458	179.67	71.13
232	*psbB-psbT*	*rpl32-trnL*	*trnL intron*	*trnR-atpA*	2753	174.38	78.26
233	*psbB-psbT*	*rpl32-trnL*	*trnL intron*	*rps16-trnQ*	3726	179.09	74.81
234	*psbB-psbT*	*rpl32-trnL*	*rpl16-rps3*	*trnE-trnT*	2690	184.36	65.13
235	*psbB-psbT*	*rpl32-trnL*	*rpl16-rps3*	*trnR-atpA*	1985	179.07	50.00
236	*psbB-psbT*	*rpl32-trnL*	*rpl16-rps3*	*rps16-trnQ*	2958	183.78	65.87
237	*psbB-psbT*	*rpl32-trnL*	*trnE-trnT*	*trnR-atpA*	2716	184.92	56.03
238	*psbB-psbT*	*rpl32-trnL*	*trnE-trnT*	*rps16-trnQ*	3689	189.63	64.81
239	*psbB-psbT*	*rpl32-trnL*	*trnR-atpA*	*rps16-trnQ*	2984	184.34	64.00
240	*psbB-psbT*	*clpP-psbB*	*trnL intron*	*rpl16-rps3*	2710	163.62	75.84
241	*psbB-psbT*	*clpP-psbB*	*trnL intron*	*trnE-trnT*	3441	169.48	65.35

242	*psbB-psbT*	*clpP-psbB*	*trnL intron*	*trnR-atpA*	2736	164.18	78.23
243	*psbB-psbT*	*clpP-psbB*	*trnL intron*	*rps16-trnQ*	3709	168.90	77.45
244	*psbB-psbT*	*clpP-psbB*	*rpl16-rps3*	*trnE-trnT*	2673	174.16	56.84
245	*psbB-psbT*	*clpP-psbB*	*rpl16-rps3*	*trnR-atpA*	1968	168.87	55.94
246	*psbB-psbT*	*clpP-psbB*	*rpl16-rps3*	*rps16-trnQ*	2941	173.58	56.39
247	*psbB-psbT*	*clpP-psbB*	*trnE-trnT*	*trnR-atpA*	2699	174.72	59.26
248	*psbB-psbT*	*clpP-psbB*	*trnE-trnT*	*rps16-trnQ*	3672	179.44	63.65
249	*psbB-psbT*	*clpP-psbB*	*trnR-atpA*	*rps16-trnQ*	2967	174.14	54.84
250	*psbB-psbT*	*trnL intron*	*rpl16-rps3*	*trnE-trnT*	2793	172.45	64.55
251	*psbB-psbT*	*trnL intron*	*rpl16-rps3*	*trnR-atpA*	2088	167.16	76.65
252	*psbB-psbT*	*trnL intron*	*rpl16-rps3*	*rps16-trnQ*	3061	171.87	77.06
253	*psbB-psbT*	*trnL intron*	*trnE-trnT*	*trnR-atpA*	2819	173.01	67.97
254	*psbB-psbT*	*trnL intron*	*trnE-trnT*	*rps16-trnQ*	3792	177.73	69.90
255	*psbB-psbT*	*trnL intron*	*trnR-atpA*	*rps16-trnQ*	3087	172.43	81.29
256	*psbB-psbT*	*rpl16-rps3*	*trnE-trnT*	*trnR-atpA*	2051	177.70	53.39
257	*psbB-psbT*	*rpl16-rps3*	*trnE-trnT*	*rps16-trnQ*	3024	182.41	60.06
258	*psbB-psbT*	*rpl16-rps3*	*trnR-atpA*	*rps16-trnQ*	2319	177.12	54.87
259	*psbB-psbT*	*trnE-trnT*	*trnR-atpA*	*rps16-trnQ*	3050	182.97	60.32
260	*ndhF-rpl32*	*trnT-trnL*	*rpl32-trnL*	*clpP-psbB*	2958	169.15	80.81
261	*ndhF-rpl32*	*trnT-trnL*	*rpl32-trnL*	*trnL intron*	3078	167.44	78.39
262	*ndhF-rpl32*	*trnT-trnL*	*rpl32-trnL*	*rpl16-rps3*	2310	172.13	73.68
263	*ndhF-rpl32*	*trnT-trnL*	*rpl32-trnL*	*trnE-trnT*	3041	177.98	65.52
264	*ndhF-rpl32*	*trnT-trnL*	*rpl32-trnL*	*trnR-atpA*	2336	172.69	79.00
265	*ndhF-rpl32*	*trnT-trnL*	*rpl32-trnL*	*rps16-trnQ*	3309	177.40	78.32
266	*ndhF-rpl32*	*trnT-trnL*	*clpP-psbB*	*trnL intron*	3061	157.24	68.68
267	*ndhF-rpl32*	*trnT-trnL*	*clpP-psbB*	*rpl16-rps3*	2293	161.93	63.77
268	*ndhF-rpl32*	*trnT-trnL*	*clpP-psbB*	*trnE-trnT*	3024	167.78	58.45

269	*ndhF-rpl32*	*trnT-trnL*	*clpP-psbB*	*trnR-atpA*	2319	162.49	65.87
270	*ndhF-rpl32*	*trnT-trnL*	*clpP-psbB*	*rps16-trnQ*	3292	167.20	67.06
271	*ndhF-rpl32*	*trnT-trnL*	*trnL intron*	*rpl16-rps3*	2413	160.22	64.48
272	*ndhF-rpl32*	*trnT-trnL*	*trnL intron*	*trnE-trnT*	3144	166.08	63.55
273	*ndhF-rpl32*	*trnT-trnL*	*trnL intron*	*trnR-atpA*	2439	160.78	67.52
274	*ndhF-rpl32*	*trnT-trnL*	*trnL intron*	*rps16-trnQ*	3412	165.50	71.61
275	*ndhF-rpl32*	*trnT-trnL*	*rpl16-rps3*	*trnE-trnT*	2376	170.76	57.35
276	*ndhF-rpl32*	*trnT-trnL*	*rpl16-rps3*	*trnR-atpA*	1671	165.47	67.68
277	*ndhF-rpl32*	*trnT-trnL*	*rpl16-rps3*	*rps16-trnQ*	2644	170.18	65.77
278	*ndhF-rpl32*	*trnT-trnL*	*trnE-trnT*	*trnR-atpA*	2402	171.32	55.61
279	*ndhF-rpl32*	*trnT-trnL*	*trnE-trnT*	*rps16-trnQ*	3375	176.04	64.23
280	*ndhF-rpl32*	*trnT-trnL*	*trnR-atpA*	*rps16-trnQ*	2670	170.74	67.45
281	*ndhF-rpl32*	*rpl32-trnL*	*clpP-psbB*	*trnL intron*	3092	158.75	77.68
282	*ndhF-rpl32*	*rpl32-trnL*	*clpP-psbB*	*rpl16-rps3*	2324	163.44	78.06
283	*ndhF-rpl32*	*rpl32-trnL*	*clpP-psbB*	*trnE-trnT*	3055	169.29	68.42
284	*ndhF-rpl32*	*rpl32-trnL*	*clpP-psbB*	*trnR-atpA*	2350	164.00	75.35
285	*ndhF-rpl32*	*rpl32-trnL*	*clpP-psbB*	*rps16-trnQ*	3323	168.71	80.03
286	*ndhF-rpl32*	*rpl32-trnL*	*trnL intron*	*rpl16-rps3*	2444	161.73	75.81
287	*ndhF-rpl32*	*rpl32-trnL*	*trnL intron*	*trnE-trnT*	3175	167.58	76.42
288	*ndhF-rpl32*	*rpl32-trnL*	*trnL intron*	*trnR-atpA*	2470	162.29	76.48
289	*ndhF-rpl32*	*rpl32-trnL*	*trnL intron*	*rps16-trnQ*	3443	167.00	81.81
290	*ndhF-rpl32*	*rpl32-trnL*	*rpl16-rps3*	*trnE-trnT*	2407	172.27	62.32
291	*ndhF-rpl32*	*rpl32-trnL*	*rpl16-rps3*	*trnR-atpA*	1702	166.97	73.84
292	*ndhF-rpl32*	*rpl32-trnL*	*rpl16-rps3*	*rps16-trnQ*	2675	171.69	74.48
293	*ndhF-rpl32*	*rpl32-trnL*	*trnE-trnT*	*trnR-atpA*	2433	172.83	66.06
294	*ndhF-rpl32*	*rpl32-trnL*	*trnE-trnT*	*rps16-trnQ*	3406	177.54	75.42
295	*ndhF-rpl32*	*rpl32-trnL*	*trnR-atpA*	*rps16-trnQ*	2701	172.25	79.13

296	*ndhF-rpl32*	*clpP-psbB*	*trnL intron*	*rpl16-rps3*	2427	151.53	72.26
297	*ndhF-rpl32*	*clpP-psbB*	*trnL intron*	*trnE-trnT*	3158	157.39	68.68
298	*ndhF-rpl32*	*clpP-psbB*	*trnL intron*	*trnR-atpA*	2453	152.09	71.81
299	*ndhF-rpl32*	*clpP-psbB*	*trnL intron*	*rps16-trnQ*	3426	156.80	74.90
300	*ndhF-rpl32*	*clpP-psbB*	*rpl16-rps3*	*trnE-trnT*	2390	162.07	55.77
301	*ndhF-rpl32*	*clpP-psbB*	*rpl16-rps3*	*trnR-atpA*	1685	156.78	69.61
302	*ndhF-rpl32*	*clpP-psbB*	*rpl16-rps3*	*rps16-trnQ*	2658	161.49	73.71
303	*ndhF-rpl32*	*clpP-psbB*	*trnE-trnT*	*trnR-atpA*	2416	162.63	54.42
304	*ndhF-rpl32*	*clpP-psbB*	*trnE-trnT*	*rps16-trnQ*	3389	167.34	68.87
305	*ndhF-rpl32*	*clpP-psbB*	*trnR-atpA*	*rps16-trnQ*	2684	162.05	70.84
306	*ndhF-rpl32*	*trnL intron*	*rpl16-rps3*	*trnE-trnT*	2510	160.36	68.84
307	*ndhF-rpl32*	*trnL intron*	*rpl16-rps3*	*trnR-atpA*	1805	155.07	71.65
308	*ndhF-rpl32*	*trnL intron*	*rpl16-rps3*	*rps16-trnQ*	2778	159.78	77.35
309	*ndhF-rpl32*	*trnL intron*	*trnE-trnT*	*trnR-atpA*	2536	160.92	67.58
310	*ndhF-rpl32*	*trnL intron*	*trnE-trnT*	*rps16-trnQ*	3509	165.64	72.39
311	*ndhF-rpl32*	*trnL intron*	*trnR-atpA*	*rps16-trnQ*	2804	160.34	78.03
312	*ndhF-rpl32*	*rpl16-rps3*	*trnE-trnT*	*trnR-atpA*	1768	165.61	50.68
313	*ndhF-rpl32*	*rpl16-rps3*	*trnE-trnT*	*rps16-trnQ*	2741	170.32	64.71
314	*ndhF-rpl32*	*rpl16-rps3*	*trnR-atpA*	*rps16-trnQ*	2036	165.03	70.29
315	*ndhF-rpl32*	*trnE-trnT*	*trnR-atpA*	*rps16-trnQ*	2767	170.88	62.77
316	*trnT-trnL*	*rpl32-trnL*	*clpP-psbB*	*trnL intron*	3535	150.51	78.10
317	*trnT-trnL*	*rpl32-trnL*	*clpP-psbB*	*rpl16-rps3*	2767	155.20	77.13
318	*trnT-trnL*	*rpl32-trnL*	*clpP-psbB*	*trnE-trnT*	3498	161.05	63.68
319	*trnT-trnL*	*rpl32-trnL*	*clpP-psbB*	*trnR-atpA*	2793	155.76	77.87
320	*trnT-trnL*	*rpl32-trnL*	*clpP-psbB*	*rps16-trnQ*	3766	160.47	78.42
321	*trnT-trnL*	*rpl32-trnL*	*trnL intron*	*rpl16-rps3*	2887	153.49	74.45
322	*trnT-trnL*	*rpl32-trnL*	*trnL intron*	*trnE-trnT*	3618	159.35	68.10

323	*trnT-trnL*	*rpl32-trnL*	*trnL intron*	*trnR-atpA*	2913	154.05	78.45
324	*trnT-trnL*	*rpl32-trnL*	*trnL intron*	*rps16-trnQ*	3886	158.76	75.94
325	*trnT-trnL*	*rpl32-trnL*	*rpl16-rps3*	*trnE-trnT*	2850	164.03	61.90
326	*trnT-trnL*	*rpl32-trnL*	*rpl16-rps3*	*trnR-atpA*	2145	158.74	70.35
327	*trnT-trnL*	*rpl32-trnL*	*rpl16-rps3*	*rps16-trnQ*	3118	163.45	75.90
328	*trnT-trnL*	*rpl32-trnL*	*trnE-trnT*	*trnR-atpA*	2876	164.59	60.52
329	*trnT-trnL*	*rpl32-trnL*	*trnE-trnT*	*rps16-trnQ*	3849	169.30	71.39
330	*trnT-trnL*	*rpl32-trnL*	*trnR-atpA*	*rps16-trnQ*	3144	164.01	77.23
331	*trnT-trnL*	*clpP-psbB*	*trnL intron*	*rpl16-rps3*	2870	143.29	67.61
332	*trnT-trnL*	*clpP-psbB*	*trnL intron*	*trnE-trnT*	3601	149.15	68.23
333	*trnT-trnL*	*clpP-psbB*	*trnL intron*	*trnR-atpA*	2896	143.85	61.94
334	*trnT-trnL*	*clpP-psbB*	*trnL intron*	*rps16-trnQ*	3869	148.57	71.39
335	*trnT-trnL*	*clpP-psbB*	*rpl16-rps3*	*trnE-trnT*	2833	153.83	59.35
336	*trnT-trnL*	*clpP-psbB*	*rpl16-rps3*	*trnR-atpA*	2128	148.54	61.94
337	*trnT-trnL*	*clpP-psbB*	*rpl16-rps3*	*rps16-trnQ*	3101	153.25	66.26
338	*trnT-trnL*	*clpP-psbB*	*trnE-trnT*	*trnR-atpA*	2859	154.39	58.58
339	*trnT-trnL*	*clpP-psbB*	*trnE-trnT*	*rps16-trnQ*	3832	159.11	66.81
340	*trnT-trnL*	*clpP-psbB*	*trnR-atpA*	*rps16-trnQ*	3127	153.81	67.23
341	*trnT-trnL*	*trnL intron*	*rpl16-rps3*	*trnE-trnT*	2953	152.12	65.39
342	*trnT-trnL*	*trnL intron*	*rpl16-rps3*	*trnR-atpA*	2248	146.83	69.19
343	*trnT-trnL*	*trnL intron*	*rpl16-rps3*	*rps16-trnQ*	3221	151.54	71.26
344	*trnT-trnL*	*trnL intron*	*trnE-trnT*	*trnR-atpA*	2979	152.69	62.58
345	*trnT-trnL*	*trnL intron*	*trnE-trnT*	*rps16-trnQ*	3952	157.40	69.00
346	*trnT-trnL*	*trnL intron*	*trnR-atpA*	*rps16-trnQ*	3247	152.10	68.90
347	*trnT-trnL*	*rpl16-rps3*	*trnE-trnT*	*trnR-atpA*	2211	157.37	53.81
348	*trnT-trnL*	*rpl16-rps3*	*trnE-trnT*	*rps16-trnQ*	3184	162.08	63.55
349	*trnT-trnL*	*rpl16-rps3*	*trnR-atpA*	*rps16-trnQ*	2479	156.79	62.26

350	*trnT-trnL*	*trnE-trnT*	*trnR-atpA*	*rps16-trnQ*	3210	162.64	62.39
351	*rpl32-trnL*	*clpP-psbB*	*trnL intron*	*rpl16-rps3*	2901	144.80	75.23
352	*rpl32-trnL*	*clpP-psbB*	*trnL intron*	*trnE-trnT*	3632	150.66	75.42
353	*rpl32-trnL*	*clpP-psbB*	*trnL intron*	*trnR-atpA*	2927	145.36	74.68
354	*rpl32-trnL*	*clpP-psbB*	*trnL intron*	*rps16-trnQ*	3900	150.07	79.06
355	*rpl32-trnL*	*clpP-psbB*	*rpl16-rps3*	*trnE-trnT*	2864	155.34	64.65
356	*rpl32-trnL*	*clpP-psbB*	*rpl16-rps3*	*trnR-atpA*	2159	150.05	72.48
357	*rpl32-trnL*	*clpP-psbB*	*rpl16-rps3*	*rps16-trnQ*	3132	154.76	80.06
358	*rpl32-trnL*	*clpP-psbB*	*trnE-trnT*	*trnR-atpA*	2890	155.90	66.00
359	*rpl32-trnL*	*clpP-psbB*	*trnE-trnT*	*rps16-trnQ*	3863	160.61	74.16
360	*rpl32-trnL*	*clpP-psbB*	*trnR-atpA*	*rps16-trnQ*	3158	155.32	79.77
361	*rpl32-trnL*	*trnL intron*	*rpl16-rps3*	*trnE-trnT*	2984	153.63	68.03
362	*rpl32-trnL*	*trnL intron*	*rpl16-rps3*	*trnR-atpA*	2279	148.34	77.90
363	*rpl32-trnL*	*trnL intron*	*rpl16-rps3*	*rps16-trnQ*	3252	153.05	79.32
364	*rpl32-trnL*	*trnL intron*	*trnE-trnT*	*trnR-atpA*	3010	154.19	70.90
365	*rpl32-trnL*	*trnL intron*	*trnE-trnT*	*rps16-trnQ*	3983	158.91	73.26
366	*rpl32-trnL*	*trnL intron*	*trnR-atpA*	*rps16-trnQ*	3278	153.61	76.71
367	*rpl32-trnL*	*rpl16-rps3*	*trnE-trnT*	*trnR-atpA*	2242	158.88	63.90
368	*rpl32-trnL*	*rpl16-rps3*	*trnE-trnT*	*rps16-trnQ*	3215	163.59	74.58
369	*rpl32-trnL*	*rpl16-rps3*	*trnR-atpA*	*rps16-trnQ*	2510	158.30	77.16
370	*rpl32-trnL*	*trnE-trnT*	*trnR-atpA*	*rps16-trnQ*	3241	164.15	72.19
371	*clpP-psbB*	*trnL intron*	*rpl16-rps3*	*trnE-trnT*	2967	143.43	65.74
372	*clpP-psbB*	*trnL intron*	*rpl16-rps3*	*trnR-atpA*	2262	138.14	64.32
373	*clpP-psbB*	*trnL intron*	*rpl16-rps3*	*rps16-trnQ*	3235	142.85	73.13
374	*clpP-psbB*	*trnL intron*	*trnE-trnT*	*trnR-atpA*	2993	144.00	62.55
375	*clpP-psbB*	*trnL intron*	*trnE-trnT*	*rps16-trnQ*	3966	148.71	73.74
376	*clpP-psbB*	*trnL intron*	*trnR-atpA*	*rps16-trnQ*	3261	143.41	74.42

377	*clpP-psbB*	*rpl16-rps3*	*trnE-trnT*	*trnR-atpA*		2225	148.68	52.45
378	*clpP-psbB*	*rpl16-rps3*	*trnE-trnT*	*rps16-trnQ*		3198	153.39	67.94
379	*clpP-psbB*	*rpl16-rps3*	*trnR-atpA*	*rps16-trnQ*		2493	148.10	71.26
380	*clpP-psbB*	*trnE-trnT*	*trnR-atpA*	*rps16-trnQ*		3224	153.95	61.58
381	*trnL intron*	*rpl16-rps3*	*trnE-trnT*	*trnR-atpA*		2345	146.97	61.61
382	*trnL intron*	*rpl16-rps3*	*trnE-trnT*	*rps16-trnQ*		3318	151.68	70.03
383	*trnL intron*	*rpl16-rps3*	*trnR-atpA*	*rps16-trnQ*		2613	146.39	71.52
384	*trnL intron*	*trnE-trnT*	*trnR-atpA*	*rps16-trnQ*		3344	152.25	70.87
385	*rpl16-rps3*	*trnE-trnT*	*trnR-atpA*	*rps16-trnQ*		2576	156.93	58.39
386	*psbB-psbT*	*ndhF-rpl32*	*trnT-trnL*	*rpl32-trnL*	*clpP-psbB*	3637	231.50	68.58
387	*psbB-psbT*	*ndhF-rpl32*	*trnT-trnL*	*rpl32-trnL*	*trnL intron*	3757	229.80	75.65
388	*psbB-psbT*	*ndhF-rpl32*	*trnT-trnL*	*rpl32-trnL*	*rpl16-rps3*	2989	234.48	67.42
389	*psbB-psbT*	*ndhF-rpl32*	*trnT-trnL*	*rpl32-trnL*	*trnE-trnT*	3720	240.34	66.52
390	*psbB-psbT*	*ndhF-rpl32*	*trnT-trnL*	*rpl32-trnL*	*trnR-atpA*	3015	235.04	70.61
391	*psbB-psbT*	*ndhF-rpl32*	*trnT-trnL*	*rpl32-trnL*	*rps16-trnQ*	3988	239.75	72.13
392	*psbB-psbT*	*ndhF-rpl32*	*trnT-trnL*	*clpP-psbB*	*trnL intron*	3740	219.60	72.94
393	*psbB-psbT*	*ndhF-rpl32*	*trnT-trnL*	*clpP-psbB*	*rpl16-rps3*	2972	224.28	61.00
394	*psbB-psbT*	*ndhF-rpl32*	*trnT-trnL*	*clpP-psbB*	*trnE-trnT*	3703	230.14	69.90
395	*psbB-psbT*	*ndhF-rpl32*	*trnT-trnL*	*clpP-psbB*	*trnR-atpA*	2998	224.84	65.32
396	*psbB-psbT*	*ndhF-rpl32*	*trnT-trnL*	*clpP-psbB*	*rps16-trnQ*	3971	229.56	67.94
397	*psbB-psbT*	*ndhF-rpl32*	*trnT-trnL*	*trnL intron*	*rpl16-rps3*	3092	222.57	70.52
398	*psbB-psbT*	*ndhF-rpl32*	*trnT-trnL*	*trnL intron*	*trnE-trnT*	3823	228.43	75.87
399	*psbB-psbT*	*ndhF-rpl32*	*trnT-trnL*	*trnL intron*	*trnR-atpA*	3118	223.14	78.42
400	*psbB-psbT*	*ndhF-rpl32*	*trnT-trnL*	*trnL intron*	*rps16-trnQ*	4091	227.85	75.29
401	*psbB-psbT*	*ndhF-rpl32*	*trnT-trnL*	*rpl16-rps3*	*trnE-trnT*	3055	233.11	65.84
402	*psbB-psbT*	*ndhF-rpl32*	*trnT-trnL*	*rpl16-rps3*	*trnR-atpA*	2350	227.82	64.97
403	*psbB-psbT*	*ndhF-rpl32*	*trnT-trnL*	*rpl16-rps3*	*rps16-trnQ*	3323	232.53	64.84

404	*psbB-psbT*	*ndhF-rpl32*	*trnT-trnL*	*trnE-trnT*	*trnR-atpA*	3081	233.68	67.61
405	*psbB-psbT*	*ndhF-rpl32*	*trnT-trnL*	*trnE-trnT*	*rps16-trnQ*	4054	238.39	71.97
406	*psbB-psbT*	*ndhF-rpl32*	*trnT-trnL*	*trnR-atpA*	*rps16-trnQ*	3349	233.09	65.06
407	*psbB-psbT*	*ndhF-rpl32*	*rpl32-trnL*	*clpP-psbB*	*trnL intron*	3771	221.10	69.23
408	*psbB-psbT*	*ndhF-rpl32*	*rpl32-trnL*	*clpP-psbB*	*rpl16-rps3*	3003	225.79	64.48
409	*psbB-psbT*	*ndhF-rpl32*	*rpl32-trnL*	*clpP-psbB*	*trnE-trnT*	3734	231.64	66.45
410	*psbB-psbT*	*ndhF-rpl32*	*rpl32-trnL*	*clpP-psbB*	*trnR-atpA*	3029	226.35	57.13
411	*psbB-psbT*	*ndhF-rpl32*	*rpl32-trnL*	*clpP-psbB*	*rps16-trnQ*	4002	231.06	72.06
412	*psbB-psbT*	*ndhF-rpl32*	*rpl32-trnL*	*trnL intron*	*rpl16-rps3*	3123	224.08	73.32
413	*psbB-psbT*	*ndhF-rpl32*	*rpl32-trnL*	*trnL intron*	*trnE-trnT*	3854	229.94	70.84
414	*psbB-psbT*	*ndhF-rpl32*	*rpl32-trnL*	*trnL intron*	*trnR-atpA*	3149	224.64	69.94
415	*psbB-psbT*	*ndhF-rpl32*	*rpl32-trnL*	*trnL intron*	*rps16-trnQ*	4122	229.36	68.06
416	*psbB-psbT*	*ndhF-rpl32*	*rpl32-trnL*	*rpl16-rps3*	*trnE-trnT*	3086	234.62	63.26
417	*psbB-psbT*	*ndhF-rpl32*	*rpl32-trnL*	*rpl16-rps3*	*trnR-atpA*	2381	229.33	60.68
418	*psbB-psbT*	*ndhF-rpl32*	*rpl32-trnL*	*rpl16-rps3*	*rps16-trnQ*	3354	234.04	69.29
419	*psbB-psbT*	*ndhF-rpl32*	*rpl32-trnL*	*trnE-trnT*	*trnR-atpA*	3112	235.18	68.19
420	*psbB-psbT*	*ndhF-rpl32*	*rpl32-trnL*	*trnE-trnT*	*rps16-trnQ*	4085	239.90	67.29
421	*psbB-psbT*	*ndhF-rpl32*	*rpl32-trnL*	*trnR-atpA*	*rps16-trnQ*	3380	234.60	70.61
422	*psbB-psbT*	*ndhF-rpl32*	*clpP-psbB*	*trnL intron*	*rpl16-rps3*	3106	213.88	76.35
423	*psbB-psbT*	*ndhF-rpl32*	*clpP-psbB*	*trnL intron*	*trnE-trnT*	3837	219.74	68.26
424	*psbB-psbT*	*ndhF-rpl32*	*clpP-psbB*	*trnL intron*	*trnR-atpA*	3132	214.44	75.58
425	*psbB-psbT*	*ndhF-rpl32*	*clpP-psbB*	*trnL intron*	*rps16-trnQ*	4105	219.16	69.71
426	*psbB-psbT*	*ndhF-rpl32*	*clpP-psbB*	*rpl16-rps3*	*trnE-trnT*	3069	224.42	63.06
427	*psbB-psbT*	*ndhF-rpl32*	*clpP-psbB*	*rpl16-rps3*	*trnR-atpA*	2364	219.13	55.55
428	*psbB-psbT*	*ndhF-rpl32*	*clpP-psbB*	*rpl16-rps3*	*rps16-trnQ*	3337	223.84	63.74
429	*psbB-psbT*	*ndhF-rpl32*	*clpP-psbB*	*trnE-trnT*	*trnR-atpA*	3095	224.98	66.55
430	*psbB-psbT*	*ndhF-rpl32*	*clpP-psbB*	*trnE-trnT*	*rps16-trnQ*	4068	229.70	66.23

431	*psbB-psbT*	*ndhF-rpl32*	*clpP-psbB*	*trnR-atpA*	*rps16-trnQ*	3363	224.40	64.74
432	*psbB-psbT*	*ndhF-rpl32*	*trnL intron*	*rpl16-rps3*	*trnE-trnT*	3189	222.71	71.81
433	*psbB-psbT*	*ndhF-rpl32*	*trnL intron*	*rpl16-rps3*	*trnR-atpA*	2484	217.42	78.52
434	*psbB-psbT*	*ndhF-rpl32*	*trnL intron*	*rpl16-rps3*	*rps16-trnQ*	3457	222.13	73.61
435	*psbB-psbT*	*ndhF-rpl32*	*trnL intron*	*trnE-trnT*	*trnR-atpA*	3215	223.28	70.52
436	*psbB-psbT*	*ndhF-rpl32*	*trnL intron*	*trnE-trnT*	*rps16-trnQ*	4188	227.99	76.81
437	*psbB-psbT*	*ndhF-rpl32*	*trnL intron*	*trnR-atpA*	*rps16-trnQ*	3483	222.70	76.32
438	*psbB-psbT*	*ndhF-rpl32*	*rpl16-rps3*	*trnE-trnT*	*trnR-atpA*	2447	227.96	57.77
439	*psbB-psbT*	*ndhF-rpl32*	*rpl16-rps3*	*trnE-trnT*	*rps16-trnQ*	3420	232.67	67.48
440	*psbB-psbT*	*ndhF-rpl32*	*rpl16-rps3*	*trnR-atpA*	*rps16-trnQ*	2715	227.38	60.68
441	*psbB-psbT*	*ndhF-rpl32*	*trnE-trnT*	*trnR-atpA*	*rps16-trnQ*	3446	233.24	66.26
442	*psbB-psbT*	*trnT-trnL*	*rpl32-trnL*	*clpP-psbB*	*trnL intron*	4214	212.87	77.00
443	*psbB-psbT*	*trnT-trnL*	*rpl32-trnL*	*clpP-psbB*	*rpl16-rps3*	3446	217.55	65.19
444	*psbB-psbT*	*trnT-trnL*	*rpl32-trnL*	*clpP-psbB*	*trnE-trnT*	4177	223.41	67.16
445	*psbB-psbT*	*trnT-trnL*	*rpl32-trnL*	*clpP-psbB*	*trnR-atpA*	3472	218.11	69.48
446	*psbB-psbT*	*trnT-trnL*	*rpl32-trnL*	*clpP-psbB*	*rps16-trnQ*	4445	222.83	72.29
447	*psbB-psbT*	*trnT-trnL*	*rpl32-trnL*	*trnL intron*	*rpl16-rps3*	3566	215.84	74.87
448	*psbB-psbT*	*trnT-trnL*	*rpl32-trnL*	*trnL intron*	*trnE-trnT*	4297	221.70	79.48
449	*psbB-psbT*	*trnT-trnL*	*rpl32-trnL*	*trnL intron*	*trnR-atpA*	3592	216.41	75.13
450	*psbB-psbT*	*trnT-trnL*	*rpl32-trnL*	*trnL intron*	*rps16-trnQ*	4565	221.12	74.29
451	*psbB-psbT*	*trnT-trnL*	*rpl32-trnL*	*rpl16-rps3*	*trnE-trnT*	3529	226.38	70.42
452	*psbB-psbT*	*trnT-trnL*	*rpl32-trnL*	*rpl16-rps3*	*trnR-atpA*	2824	221.09	71.97
453	*psbB-psbT*	*trnT-trnL*	*rpl32-trnL*	*rpl16-rps3*	*rps16-trnQ*	3797	225.80	67.10
454	*psbB-psbT*	*trnT-trnL*	*rpl32-trnL*	*trnE-trnT*	*trnR-atpA*	3555	226.95	76.71
455	*psbB-psbT*	*trnT-trnL*	*rpl32-trnL*	*trnE-trnT*	*rps16-trnQ*	4528	231.66	73.97
456	*psbB-psbT*	*trnT-trnL*	*rpl32-trnL*	*trnR-atpA*	*rps16-trnQ*	3823	226.36	70.42
457	*psbB-psbT*	*trnT-trnL*	*clpP-psbB*	*trnL intron*	*rpl16-rps3*	3549	205.64	73.48

458	*psbB-psbT*	*trnT-trnL*	*clpP-psbB*	*trnL intron*	*trnE-trnT*	4280	211.50	71.81
459	*psbB-psbT*	*trnT-trnL*	*clpP-psbB*	*trnL intron*	*trnR-atpA*	3575	206.21	73.10
460	*psbB-psbT*	*trnT-trnL*	*clpP-psbB*	*trnL intron*	*rps16-trnQ*	4548	210.92	70.74
461	*psbB-psbT*	*trnT-trnL*	*clpP-psbB*	*rpl16-rps3*	*trnE-trnT*	3512	216.19	65.29
462	*psbB-psbT*	*trnT-trnL*	*clpP-psbB*	*rpl16-rps3*	*trnR-atpA*	2807	210.89	62.52
463	*psbB-psbT*	*trnT-trnL*	*clpP-psbB*	*rpl16-rps3*	*rps16-trnQ*	3780	215.60	68.97
464	*psbB-psbT*	*trnT-trnL*	*clpP-psbB*	*trnE-trnT*	*trnR-atpA*	3538	216.75	64.68
465	*psbB-psbT*	*trnT-trnL*	*clpP-psbB*	*trnE-trnT*	*rps16-trnQ*	4511	221.46	68.03
466	*psbB-psbT*	*trnT-trnL*	*clpP-psbB*	*trnR-atpA*	*rps16-trnQ*	3806	216.17	66.06
467	*psbB-psbT*	*trnT-trnL*	*trnL intron*	*rpl16-rps3*	*trnE-trnT*	3632	214.48	75.65
468	*psbB-psbT*	*trnT-trnL*	*trnL intron*	*rpl16-rps3*	*trnR-atpA*	2927	209.18	71.68
469	*psbB-psbT*	*trnT-trnL*	*trnL intron*	*rpl16-rps3*	*rps16-trnQ*	3900	213.90	68.65
470	*psbB-psbT*	*trnT-trnL*	*trnL intron*	*trnE-trnT*	*trnR-atpA*	3658	215.04	73.87
471	*psbB-psbT*	*trnT-trnL*	*trnL intron*	*trnE-trnT*	*rps16-trnQ*	4631	219.75	75.32
472	*psbB-psbT*	*trnT-trnL*	*trnL intron*	*trnR-atpA*	*rps16-trnQ*	3926	214.46	75.06
473	*psbB-psbT*	*trnT-trnL*	*rpl16-rps3*	*trnE-trnT*	*trnR-atpA*	2890	219.72	66.10
474	*psbB-psbT*	*trnT-trnL*	*rpl16-rps3*	*trnE-trnT*	*rps16-trnQ*	3863	224.44	70.71
475	*psbB-psbT*	*trnT-trnL*	*rpl16-rps3*	*trnR-atpA*	*rps16-trnQ*	3158	219.14	63.77
476	*psbB-psbT*	*trnT-trnL*	*trnE-trnT*	*trnR-atpA*	*rps16-trnQ*	3889	225.00	69.61
477	*psbB-psbT*	*rpl32-trnL*	*clpP-psbB*	*trnL intron*	*rpl16-rps3*	3580	207.15	71.29
478	*psbB-psbT*	*rpl32-trnL*	*clpP-psbB*	*trnL intron*	*trnE-trnT*	4311	213.01	68.32
479	*psbB-psbT*	*rpl32-trnL*	*clpP-psbB*	*trnL intron*	*trnR-atpA*	3606	207.71	78.97
480	*psbB-psbT*	*rpl32-trnL*	*clpP-psbB*	*trnL intron*	*rps16-trnQ*	4579	212.43	69.23
481	*psbB-psbT*	*rpl32-trnL*	*clpP-psbB*	*rpl16-rps3*	*trnE-trnT*	3543	217.69	60.55
482	*psbB-psbT*	*rpl32-trnL*	*clpP-psbB*	*rpl16-rps3*	*trnR-atpA*	2838	212.40	59.26
483	*psbB-psbT*	*rpl32-trnL*	*clpP-psbB*	*rpl16-rps3*	*rps16-trnQ*	3811	217.11	65.23
484	*psbB-psbT*	*rpl32-trnL*	*clpP-psbB*	*trnE-trnT*	*trnR-atpA*	3569	218.25	61.23

485	*psbB-psbT*	*rpl32-trnL*	*clpP-psbB*	*trnE-trnT*	*rps16-trnQ*	4542	222.97	66.06
486	*psbB-psbT*	*rpl32-trnL*	*clpP-psbB*	*trnR-atpA*	*rps16-trnQ*	3837	217.67	69.06
487	*psbB-psbT*	*rpl32-trnL*	*trnL intron*	*rpl16-rps3*	*trnE-trnT*	3663	215.98	73.03
488	*psbB-psbT*	*rpl32-trnL*	*trnL intron*	*rpl16-rps3*	*trnR-atpA*	2958	210.69	74.94
489	*psbB-psbT*	*rpl32-trnL*	*trnL intron*	*rpl16-rps3*	*rps16-trnQ*	3931	215.40	72.32
490	*psbB-psbT*	*rpl32-trnL*	*trnL intron*	*trnE-trnT*	*trnR-atpA*	3689	216.55	68.03
491	*psbB-psbT*	*rpl32-trnL*	*trnL intron*	*trnE-trnT*	*rps16-trnQ*	4662	221.26	73.52
492	*psbB-psbT*	*rpl32-trnL*	*trnL intron*	*trnR-atpA*	*rps16-trnQ*	3957	215.97	77.10
493	*psbB-psbT*	*rpl32-trnL*	*rpl16-rps3*	*trnE-trnT*	*trnR-atpA*	2921	221.23	63.65
494	*psbB-psbT*	*rpl32-trnL*	*rpl16-rps3*	*trnE-trnT*	*rps16-trnQ*	3894	225.94	65.23
495	*psbB-psbT*	*rpl32-trnL*	*rpl16-rps3*	*trnR-atpA*	*rps16-trnQ*	3189	220.65	61.61
496	*psbB-psbT*	*rpl32-trnL*	*trnE-trnT*	*trnR-atpA*	*rps16-trnQ*	3920	226.51	67.35
497	*psbB-psbT*	*clpP-psbB*	*trnL intron*	*rpl16-rps3*	*trnE-trnT*	3646	205.79	65.45
498	*psbB-psbT*	*clpP-psbB*	*trnL intron*	*rpl16-rps3*	*trnR-atpA*	2941	200.49	76.32
499	*psbB-psbT*	*clpP-psbB*	*trnL intron*	*rpl16-rps3*	*rps16-trnQ*	3914	205.21	72.48
500	*psbB-psbT*	*clpP-psbB*	*trnL intron*	*trnE-trnT*	*trnR-atpA*	3672	206.35	65.39
501	*psbB-psbT*	*clpP-psbB*	*trnL intron*	*trnE-trnT*	*rps16-trnQ*	4645	211.06	71.65
502	*psbB-psbT*	*clpP-psbB*	*trnL intron*	*trnR-atpA*	*rps16-trnQ*	3940	205.77	77.90
503	*psbB-psbT*	*clpP-psbB*	*rpl16-rps3*	*trnE-trnT*	*trnR-atpA*	2904	211.03	65.10
504	*psbB-psbT*	*clpP-psbB*	*rpl16-rps3*	*trnE-trnT*	*rps16-trnQ*	3877	215.75	65.42
505	*psbB-psbT*	*clpP-psbB*	*rpl16-rps3*	*trnR-atpA*	*rps16-trnQ*	3172	210.45	53.84
506	*psbB-psbT*	*clpP-psbB*	*trnE-trnT*	*trnR-atpA*	*rps16-trnQ*	3903	216.31	66.45
507	*psbB-psbT*	*trnL intron*	*rpl16-rps3*	*trnE-trnT*	*trnR-atpA*	3024	209.32	68.68
508	*psbB-psbT*	*trnL intron*	*rpl16-rps3*	*trnE-trnT*	*rps16-trnQ*	3997	214.04	73.06
509	*psbB-psbT*	*trnL intron*	*rpl16-rps3*	*trnR-atpA*	*rps16-trnQ*	3292	208.74	76.87
510	*psbB-psbT*	*trnL intron*	*trnE-trnT*	*trnR-atpA*	*rps16-trnQ*	4023	214.60	71.45
511	*psbB-psbT*	*rpl16-rps3*	*trnE-trnT*	*trnR-atpA*	*rps16-trnQ*	3255	219.28	60.84

512	*ndhF-rpl32*	*trnT-trnL*	*rpl32-trnL*	*clpP-psbB*	*trnL intron*	3931	200.78	81.94
513	*ndhF-rpl32*	*trnT-trnL*	*rpl32-trnL*	*clpP-psbB*	*rpl16-rps3*	3163	205.46	80.94
514	*ndhF-rpl32*	*trnT-trnL*	*rpl32-trnL*	*clpP-psbB*	*trnE-trnT*	3894	211.32	67.84
515	*ndhF-rpl32*	*trnT-trnL*	*rpl32-trnL*	*clpP-psbB*	*trnR-atpA*	3189	206.02	80.19
516	*ndhF-rpl32*	*trnT-trnL*	*rpl32-trnL*	*clpP-psbB*	*rps16-trnQ*	4162	210.73	82.23
517	*ndhF-rpl32*	*trnT-trnL*	*rpl32-trnL*	*trnL intron*	*rpl16-rps3*	3283	203.75	76.84
518	*ndhF-rpl32*	*trnT-trnL*	*rpl32-trnL*	*trnL intron*	*trnE-trnT*	4014	209.61	72.55
519	*ndhF-rpl32*	*trnT-trnL*	*rpl32-trnL*	*trnL intron*	*trnR-atpA*	3309	204.31	77.26
520	*ndhF-rpl32*	*trnT-trnL*	*rpl32-trnL*	*trnL intron*	*rps16-trnQ*	4282	209.03	80.97
521	*ndhF-rpl32*	*trnT-trnL*	*rpl32-trnL*	*rpl16-rps3*	*trnE-trnT*	3246	214.29	69.23
522	*ndhF-rpl32*	*trnT-trnL*	*rpl32-trnL*	*rpl16-rps3*	*trnR-atpA*	2541	209.00	76.48
523	*ndhF-rpl32*	*trnT-trnL*	*rpl32-trnL*	*rpl16-rps3*	*rps16-trnQ*	3514	213.71	79.74
524	*ndhF-rpl32*	*trnT-trnL*	*rpl32-trnL*	*trnE-trnT*	*trnR-atpA*	3272	214.85	68.94
525	*ndhF-rpl32*	*trnT-trnL*	*rpl32-trnL*	*trnE-trnT*	*rps16-trnQ*	4245	219.57	76.81
526	*ndhF-rpl32*	*trnT-trnL*	*rpl32-trnL*	*trnR-atpA*	*rps16-trnQ*	3540	214.27	80.23
527	*ndhF-rpl32*	*trnT-trnL*	*clpP-psbB*	*trnL intron*	*rpl16-rps3*	3266	193.55	73.23
528	*ndhF-rpl32*	*trnT-trnL*	*clpP-psbB*	*trnL intron*	*trnE-trnT*	3997	199.41	68.45
529	*ndhF-rpl32*	*trnT-trnL*	*clpP-psbB*	*trnL intron*	*trnR-atpA*	3292	194.12	72.97
530	*ndhF-rpl32*	*trnT-trnL*	*clpP-psbB*	*trnL intron*	*rps16-trnQ*	4265	198.83	72.81
531	*ndhF-rpl32*	*trnT-trnL*	*clpP-psbB*	*rpl16-rps3*	*trnE-trnT*	3229	204.09	58.68
532	*ndhF-rpl32*	*trnT-trnL*	*clpP-psbB*	*rpl16-rps3*	*trnR-atpA*	2524	198.80	68.68
533	*ndhF-rpl32*	*trnT-trnL*	*clpP-psbB*	*rpl16-rps3*	*rps16-trnQ*	3497	203.51	70.45
534	*ndhF-rpl32*	*trnT-trnL*	*clpP-psbB*	*trnE-trnT*	*trnR-atpA*	3255	204.66	56.55
535	*ndhF-rpl32*	*trnT-trnL*	*clpP-psbB*	*trnE-trnT*	*rps16-trnQ*	4228	209.37	71.35
536	*ndhF-rpl32*	*trnT-trnL*	*clpP-psbB*	*trnR-atpA*	*rps16-trnQ*	3523	204.07	72.45
537	*ndhF-rpl32*	*trnT-trnL*	*trnL intron*	*rpl16-rps3*	*trnE-trnT*	3349	202.39	70.16
538	*ndhF-rpl32*	*trnT-trnL*	*trnL intron*	*rpl16-rps3*	*trnR-atpA*	2644	197.09	67.19

539	*ndhF-rpl32*	*trnT-trnL*	*trnL intron*	*rpl16-rps3*	*rps16-trnQ*	3617	201.80	70.97
540	*ndhF-rpl32*	*trnT-trnL*	*trnL intron*	*trnE-trnT*	*trnR-atpA*	3375	202.95	66.06
541	*ndhF-rpl32*	*trnT-trnL*	*trnL intron*	*trnE-trnT*	*rps16-trnQ*	4348	207.66	73.03
542	*ndhF-rpl32*	*trnT-trnL*	*trnL intron*	*trnR-atpA*	*rps16-trnQ*	3643	202.37	72.97
543	*ndhF-rpl32*	*trnT-trnL*	*rpl16-rps3*	*trnE-trnT*	*trnR-atpA*	2607	207.63	60.58
544	*ndhF-rpl32*	*trnT-trnL*	*rpl16-rps3*	*trnE-trnT*	*rps16-trnQ*	3580	212.34	64.90
545	*ndhF-rpl32*	*trnT-trnL*	*rpl16-rps3*	*trnR-atpA*	*rps16-trnQ*	2875	207.05	66.45
546	*ndhF-rpl32*	*trnT-trnL*	*trnE-trnT*	*trnR-atpA*	*rps16-trnQ*	3606	212.91	64.61
547	*ndhF-rpl32*	*rpl32-trnL*	*clpP-psbB*	*trnL intron*	*rpl16-rps3*	3297	195.06	80.90
548	*ndhF-rpl32*	*rpl32-trnL*	*clpP-psbB*	*trnL intron*	*trnE-trnT*	4028	200.92	78.58
549	*ndhF-rpl32*	*rpl32-trnL*	*clpP-psbB*	*trnL intron*	*trnR-atpA*	3323	195.62	78.97
550	*ndhF-rpl32*	*rpl32-trnL*	*clpP-psbB*	*trnL intron*	*rps16-trnQ*	4296	200.34	80.16
551	*ndhF-rpl32*	*rpl32-trnL*	*clpP-psbB*	*rpl16-rps3*	*trnE-trnT*	3260	205.60	71.19
552	*ndhF-rpl32*	*rpl32-trnL*	*clpP-psbB*	*rpl16-rps3*	*trnR-atpA*	2555	200.31	78.19
553	*ndhF-rpl32*	*rpl32-trnL*	*clpP-psbB*	*rpl16-rps3*	*rps16-trnQ*	3528	205.02	80.23
554	*ndhF-rpl32*	*rpl32-trnL*	*clpP-psbB*	*trnE-trnT*	*trnR-atpA*	3286	206.16	70.71
555	*ndhF-rpl32*	*rpl32-trnL*	*clpP-psbB*	*trnE-trnT*	*rps16-trnQ*	4259	210.88	76.48
556	*ndhF-rpl32*	*rpl32-trnL*	*clpP-psbB*	*trnR-atpA*	*rps16-trnQ*	3554	205.58	80.97
557	*ndhF-rpl32*	*rpl32-trnL*	*trnL intron*	*rpl16-rps3*	*trnE-trnT*	3380	203.89	75.61
558	*ndhF-rpl32*	*rpl32-trnL*	*trnL intron*	*rpl16-rps3*	*trnR-atpA*	2675	198.60	77.58
559	*ndhF-rpl32*	*rpl32-trnL*	*trnL intron*	*rpl16-rps3*	*rps16-trnQ*	3648	203.31	81.84
560	*ndhF-rpl32*	*rpl32-trnL*	*trnL intron*	*trnE-trnT*	*trnR-atpA*	3406	204.46	76.42
561	*ndhF-rpl32*	*rpl32-trnL*	*trnL intron*	*trnE-trnT*	*rps16-trnQ*	4379	209.17	78.65
562	*ndhF-rpl32*	*rpl32-trnL*	*trnL intron*	*trnR-atpA*	*rps16-trnQ*	3674	203.87	79.97
563	*ndhF-rpl32*	*rpl32-trnL*	*rpl16-rps3*	*trnE-trnT*	*trnR-atpA*	2638	209.14	69.71
564	*ndhF-rpl32*	*rpl32-trnL*	*rpl16-rps3*	*trnE-trnT*	*rps16-trnQ*	3611	213.85	77.13
565	*ndhF-rpl32*	*rpl32-trnL*	*rpl16-rps3*	*trnR-atpA*	*rps16-trnQ*	2906	208.56	79.68

566	*ndhF-rpl32*	*rpl32-trnL*	*trnE-trnT*	*trnR-atpA*	*rps16-trnQ*	3637	214.41	77.35
567	*ndhF-rpl32*	*clpP-psbB*	*trnL intron*	*rpl16-rps3*	*trnE-trnT*	3363	193.70	72.84
568	*ndhF-rpl32*	*clpP-psbB*	*trnL intron*	*rpl16-rps3*	*trnR-atpA*	2658	188.40	73.00
569	*ndhF-rpl32*	*clpP-psbB*	*trnL intron*	*rpl16-rps3*	*rps16-trnQ*	3631	193.11	77.13
570	*ndhF-rpl32*	*clpP-psbB*	*trnL intron*	*trnE-trnT*	*trnR-atpA*	3389	194.26	70.74
571	*ndhF-rpl32*	*clpP-psbB*	*trnL intron*	*trnE-trnT*	*rps16-trnQ*	4362	198.97	73.35
572	*ndhF-rpl32*	*clpP-psbB*	*trnL intron*	*trnR-atpA*	*rps16-trnQ*	3657	193.68	79.55
573	*ndhF-rpl32*	*clpP-psbB*	*rpl16-rps3*	*trnE-trnT*	*trnR-atpA*	2621	198.94	57.71
574	*ndhF-rpl32*	*clpP-psbB*	*rpl16-rps3*	*trnE-trnT*	*rps16-trnQ*	3594	203.65	66.35
575	*ndhF-rpl32*	*clpP-psbB*	*rpl16-rps3*	*trnR-atpA*	*rps16-trnQ*	2889	198.36	73.90
576	*ndhF-rpl32*	*clpP-psbB*	*trnE-trnT*	*trnR-atpA*	*rps16-trnQ*	3620	204.22	65.74
577	*ndhF-rpl32*	*trnL intron*	*rpl16-rps3*	*trnE-trnT*	*trnR-atpA*	2741	197.23	69.84
578	*ndhF-rpl32*	*trnL intron*	*rpl16-rps3*	*trnE-trnT*	*rps16-trnQ*	3714	201.95	74.32
579	*ndhF-rpl32*	*trnL intron*	*rpl16-rps3*	*trnR-atpA*	*rps16-trnQ*	3009	196.65	78.06
580	*ndhF-rpl32*	*trnL intron*	*trnE-trnT*	*trnR-atpA*	*rps16-trnQ*	3740	202.51	74.39
581	*ndhF-rpl32*	*rpl16-rps3*	*trnE-trnT*	*trnR-atpA*	*rps16-trnQ*	2972	207.19	68.65
582	*trnT-trnL*	*rpl32-trnL*	*clpP-psbB*	*trnL intron*	*rpl16-rps3*	3740	186.82	79.16
583	*trnT-trnL*	*rpl32-trnL*	*clpP-psbB*	*trnL intron*	*trnE-trnT*	4471	192.68	72.06
584	*trnT-trnL*	*rpl32-trnL*	*clpP-psbB*	*trnL intron*	*trnR-atpA*	3766	187.39	79.23
585	*trnT-trnL*	*rpl32-trnL*	*clpP-psbB*	*trnL intron*	*rps16-trnQ*	4739	192.10	83.10
586	*trnT-trnL*	*rpl32-trnL*	*clpP-psbB*	*rpl16-rps3*	*trnE-trnT*	3703	197.36	67.71
587	*trnT-trnL*	*rpl32-trnL*	*clpP-psbB*	*rpl16-rps3*	*trnR-atpA*	2998	192.07	78.16
588	*trnT-trnL*	*rpl32-trnL*	*clpP-psbB*	*rpl16-rps3*	*rps16-trnQ*	3971	196.78	80.16
589	*trnT-trnL*	*rpl32-trnL*	*clpP-psbB*	*trnE-trnT*	*trnR-atpA*	3729	197.93	66.16
590	*trnT-trnL*	*rpl32-trnL*	*clpP-psbB*	*trnE-trnT*	*rps16-trnQ*	4702	202.64	71.06
591	*trnT-trnL*	*rpl32-trnL*	*clpP-psbB*	*trnR-atpA*	*rps16-trnQ*	3997	197.34	79.97
592	*trnT-trnL*	*rpl32-trnL*	*trnL intron*	*rpl16-rps3*	*trnE-trnT*	3823	195.66	71.42

593	*trnT-trnL*	*rpl32-trnL*	*trnL intron*	*rpl16-rps3*	*trnR-atpA*	3118	190.36	77.29
594	*trnT-trnL*	*rpl32-trnL*	*trnL intron*	*rpl16-rps3*	*rps16-trnQ*	4091	195.07	77.97
595	*trnT-trnL*	*rpl32-trnL*	*trnL intron*	*trnE-trnT*	*trnR-atpA*	3849	196.22	69.90
596	*trnT-trnL*	*rpl32-trnL*	*trnL intron*	*trnE-trnT*	*rps16-trnQ*	4822	200.93	74.68
597	*trnT-trnL*	*rpl32-trnL*	*trnL intron*	*trnR-atpA*	*rps16-trnQ*	4117	195.64	78.29
598	*trnT-trnL*	*rpl32-trnL*	*rpl16-rps3*	*trnE-trnT*	*trnR-atpA*	3081	200.90	65.81
599	*trnT-trnL*	*rpl32-trnL*	*rpl16-rps3*	*trnE-trnT*	*rps16-trnQ*	4054	205.61	71.23
600	*trnT-trnL*	*rpl32-trnL*	*rpl16-rps3*	*trnR-atpA*	*rps16-trnQ*	3349	200.32	75.39
601	*trnT-trnL*	*rpl32-trnL*	*trnE-trnT*	*trnR-atpA*	*rps16-trnQ*	4080	206.18	72.74
602	*trnT-trnL*	*clpP-psbB*	*trnL intron*	*rpl16-rps3*	*trnE-trnT*	3806	185.46	68.55
603	*trnT-trnL*	*clpP-psbB*	*trnL intron*	*rpl16-rps3*	*trnR-atpA*	3101	180.16	67.65
604	*trnT-trnL*	*clpP-psbB*	*trnL intron*	*rpl16-rps3*	*rps16-trnQ*	4074	184.88	72.39
605	*trnT-trnL*	*clpP-psbB*	*trnL intron*	*trnE-trnT*	*trnR-atpA*	3832	186.02	64.81
606	*trnT-trnL*	*clpP-psbB*	*trnL intron*	*trnE-trnT*	*rps16-trnQ*	4805	190.73	72.39
607	*trnT-trnL*	*clpP-psbB*	*trnL intron*	*trnR-atpA*	*rps16-trnQ*	4100	185.44	71.94
608	*trnT-trnL*	*clpP-psbB*	*rpl16-rps3*	*trnE-trnT*	*trnR-atpA*	3064	190.70	58.84
609	*trnT-trnL*	*clpP-psbB*	*rpl16-rps3*	*trnE-trnT*	*rps16-trnQ*	4037	195.42	67.84
610	*trnT-trnL*	*clpP-psbB*	*rpl16-rps3*	*trnR-atpA*	*rps16-trnQ*	3332	190.12	66.00
611	*trnT-trnL*	*clpP-psbB*	*trnE-trnT*	*trnR-atpA*	*rps16-trnQ*	4063	195.98	65.06
612	*trnT-trnL*	*trnL intron*	*rpl16-rps3*	*trnE-trnT*	*trnR-atpA*	3184	189.00	63.94
613	*trnT-trnL*	*trnL intron*	*rpl16-rps3*	*trnE-trnT*	*rps16-trnQ*	4157	193.71	69.61
614	*trnT-trnL*	*trnL intron*	*rpl16-rps3*	*trnR-atpA*	*rps16-trnQ*	3452	188.41	70.58
615	*trnT-trnL*	*trnL intron*	*trnE-trnT*	*trnR-atpA*	*rps16-trnQ*	4183	194.27	70.39
616	*trnT-trnL*	*rpl16-rps3*	*trnE-trnT*	*trnR-atpA*	*rps16-trnQ*	3415	198.95	62.65
617	*rpl32-trnL*	*clpP-psbB*	*trnL intron*	*rpl16-rps3*	*trnE-trnT*	3837	186.96	74.77
618	*rpl32-trnL*	*clpP-psbB*	*trnL intron*	*rpl16-rps3*	*trnR-atpA*	3132	181.67	76.90
619	*rpl32-trnL*	*clpP-psbB*	*trnL intron*	*rpl16-rps3*	*rps16-trnQ*	4105	186.38	79.39

620	*rpl32-trnL*	*clpP-psbB*	*trnL intron*	*trnE-trnT*	*trnR-atpA*		3863	187.53	76.13
621	*rpl32-trnL*	*clpP-psbB*	*trnL intron*	*trnE-trnT*	*rps16-trnQ*		4836	192.24	76.35
622	*rpl32-trnL*	*clpP-psbB*	*trnL intron*	*trnR-atpA*	*rps16-trnQ*		4131	186.95	78.52
623	*rpl32-trnL*	*clpP-psbB*	*rpl16-rps3*	*trnE-trnT*	*trnR-atpA*		3095	192.21	68.16
624	*rpl32-trnL*	*clpP-psbB*	*rpl16-rps3*	*trnE-trnT*	*rps16-trnQ*		4068	196.92	74.39
625	*rpl32-trnL*	*clpP-psbB*	*rpl16-rps3*	*trnR-atpA*	*rps16-trnQ*		3363	191.63	79.39
626	*rpl32-trnL*	*clpP-psbB*	*trnE-trnT*	*trnR-atpA*	*rps16-trnQ*		4094	197.49	75.61
627	*rpl32-trnL*	*trnL intron*	*rpl16-rps3*	*trnE-trnT*	*trnR-atpA*		3215	190.50	71.84
628	*rpl32-trnL*	*trnL intron*	*rpl16-rps3*	*trnE-trnT*	*rps16-trnQ*		4188	195.22	75.52
629	*rpl32-trnL*	*trnL intron*	*rpl16-rps3*	*trnR-atpA*	*rps16-trnQ*		3483	189.92	75.94
630	*rpl32-trnL*	*trnL intron*	*trnE-trnT*	*trnR-atpA*	*rps16-trnQ*		4214	195.78	73.65
631	*rpl32-trnL*	*rpl16-rps3*	*trnE-trnT*	*trnR-atpA*	*rps16-trnQ*		3446	200.46	74.39
632	*clpP-psbB*	*trnL intron*	*rpl16-rps3*	*trnE-trnT*	*trnR-atpA*		3198	180.30	66.94
633	*clpP-psbB*	*trnL intron*	*rpl16-rps3*	*trnE-trnT*	*rps16-trnQ*		4171	185.02	70.77
634	*clpP-psbB*	*trnL intron*	*rpl16-rps3*	*trnR-atpA*	*rps16-trnQ*		3466	179.72	73.71
635	*clpP-psbB*	*trnL intron*	*trnE-trnT*	*trnR-atpA*	*rps16-trnQ*		4197	185.58	75.61
636	*clpP-psbB*	*rpl16-rps3*	*trnE-trnT*	*trnR-atpA*	*rps16-trnQ*		3429	190.26	63.94
637	*trnL intron*	*rpl16-rps3*	*trnE-trnT*	*trnR-atpA*	*rps16-trnQ*		3549	188.56	71.61
638	*psbB-psbT*	*ndhF-rpl32*	*trnT-trnL*	*rpl32-trnL*	*clpP-psbB*	*trnL intron*	4610	263.13	73.42
639	*psbB-psbT*	*ndhF-rpl32*	*trnT-trnL*	*rpl32-trnL*	*clpP-psbB*	*rpl16-rps3*	3842	267.81	68.97
640	*psbB-psbT*	*ndhF-rpl32*	*trnT-trnL*	*rpl32-trnL*	*clpP-psbB*	*trnE-trnT*	4573	273.67	73.03
641	*psbB-psbT*	*ndhF-rpl32*	*trnT-trnL*	*rpl32-trnL*	*clpP-psbB*	*trnR-atpA*	3868	268.38	71.81
642	*psbB-psbT*	*ndhF-rpl32*	*trnT-trnL*	*rpl32-trnL*	*clpP-psbB*	*rps16-trnQ*	4841	273.09	74.29
643	*psbB-psbT*	*ndhF-rpl32*	*trnT-trnL*	*rpl32-trnL*	*trnL intron*	*rpl16-rps3*	3962	266.10	77.65
644	*psbB-psbT*	*ndhF-rpl32*	*trnT-trnL*	*rpl32-trnL*	*trnL intron*	*trnE-trnT*	4693	271.96	76.03
645	*psbB-psbT*	*ndhF-rpl32*	*trnT-trnL*	*rpl32-trnL*	*trnL intron*	*trnR-atpA*	3988	266.67	76.13
646	*psbB-psbT*	*ndhF-rpl32*	*trnT-trnL*	*rpl32-trnL*	*trnL intron*	*rps16-trnQ*	4961	271.38	74.81

647	*psbB-psbT*	*ndhF-rpl32*	*trnT-trnL*	*rpl32-trnL*	*rpl16-rps3*	*trnE-trnT*	3925	276.64	73.48
648	*psbB-psbT*	*ndhF-rpl32*	*trnT-trnL*	*rpl32-trnL*	*rpl16-rps3*	*trnR-atpA*	3220	271.35	65.39
649	*psbB-psbT*	*ndhF-rpl32*	*trnT-trnL*	*rpl32-trnL*	*rpl16-rps3*	*rps16-trnQ*	4193	276.06	72.45
650	*psbB-psbT*	*ndhF-rpl32*	*trnT-trnL*	*rpl32-trnL*	*trnE-trnT*	*trnR-atpA*	3951	277.21	72.32
651	*psbB-psbT*	*ndhF-rpl32*	*trnT-trnL*	*rpl32-trnL*	*trnE-trnT*	*rps16-trnQ*	4924	281.92	76.77
652	*psbB-psbT*	*ndhF-rpl32*	*trnT-trnL*	*rpl32-trnL*	*trnR-atpA*	*rps16-trnQ*	4219	276.63	72.03
653	*psbB-psbT*	*ndhF-rpl32*	*trnT-trnL*	*clpP-psbB*	*trnL intron*	*rpl16-rps3*	3945	255.91	69.19
654	*psbB-psbT*	*ndhF-rpl32*	*trnT-trnL*	*clpP-psbB*	*trnL intron*	*trnE-trnT*	4676	261.76	77.35
655	*psbB-psbT*	*ndhF-rpl32*	*trnT-trnL*	*clpP-psbB*	*trnL intron*	*trnR-atpA*	3971	256.47	72.58
656	*psbB-psbT*	*ndhF-rpl32*	*trnT-trnL*	*clpP-psbB*	*trnL intron*	*rps16-trnQ*	4944	261.18	73.94
657	*psbB-psbT*	*ndhF-rpl32*	*trnT-trnL*	*clpP-psbB*	*rpl16-rps3*	*trnE-trnT*	3908	266.45	69.45
658	*psbB-psbT*	*ndhF-rpl32*	*trnT-trnL*	*clpP-psbB*	*rpl16-rps3*	*trnR-atpA*	3203	261.15	65.48
659	*psbB-psbT*	*ndhF-rpl32*	*trnT-trnL*	*clpP-psbB*	*rpl16-rps3*	*rps16-trnQ*	4176	265.87	68.48
660	*psbB-psbT*	*ndhF-rpl32*	*trnT-trnL*	*clpP-psbB*	*trnE-trnT*	*trnR-atpA*	3934	267.01	69.39
661	*psbB-psbT*	*ndhF-rpl32*	*trnT-trnL*	*clpP-psbB*	*trnE-trnT*	*rps16-trnQ*	4907	271.72	72.19
662	*psbB-psbT*	*ndhF-rpl32*	*trnT-trnL*	*clpP-psbB*	*trnR-atpA*	*rps16-trnQ*	4202	266.43	68.52
663	*psbB-psbT*	*ndhF-rpl32*	*trnT-trnL*	*trnL intron*	*rpl16-rps3*	*trnE-trnT*	4028	264.74	78.90
664	*psbB-psbT*	*ndhF-rpl32*	*trnT-trnL*	*trnL intron*	*rpl16-rps3*	*trnR-atpA*	3323	259.44	72.77
665	*psbB-psbT*	*ndhF-rpl32*	*trnT-trnL*	*trnL intron*	*rpl16-rps3*	*rps16-trnQ*	4296	264.16	70.81
666	*psbB-psbT*	*ndhF-rpl32*	*trnT-trnL*	*trnL intron*	*trnE-trnT*	*trnR-atpA*	4054	265.30	78.68
667	*psbB-psbT*	*ndhF-rpl32*	*trnT-trnL*	*trnL intron*	*trnE-trnT*	*rps16-trnQ*	5027	270.01	80.55
668	*psbB-psbT*	*ndhF-rpl32*	*trnT-trnL*	*trnL intron*	*trnR-atpA*	*rps16-trnQ*	4322	264.72	67.84
669	*psbB-psbT*	*ndhF-rpl32*	*trnT-trnL*	*rpl16-rps3*	*trnE-trnT*	*trnR-atpA*	3286	269.99	69.52
670	*psbB-psbT*	*ndhF-rpl32*	*trnT-trnL*	*rpl16-rps3*	*trnE-trnT*	*rps16-trnQ*	4259	274.70	71.48
671	*psbB-psbT*	*ndhF-rpl32*	*trnT-trnL*	*rpl16-rps3*	*trnR-atpA*	*rps16-trnQ*	3554	269.40	66.84
672	*psbB-psbT*	*ndhF-rpl32*	*trnT-trnL*	*trnE-trnT*	*trnR-atpA*	*rps16-trnQ*	4285	275.26	71.97
673	*psbB-psbT*	*ndhF-rpl32*	*rpl32-trnL*	*clpP-psbB*	*trnL intron*	*rpl16-rps3*	3976	257.41	67.19

674	*psbB-psbT*	*ndhF-rpl32*	*rpl32-trnL*	*clpP-psbB*	*trnL intron*	*trnE-trnT*	4707	263.27	71.90
675	*psbB-psbT*	*ndhF-rpl32*	*rpl32-trnL*	*clpP-psbB*	*trnL intron*	*trnR-atpA*	4002	257.98	69.45
676	*psbB-psbT*	*ndhF-rpl32*	*rpl32-trnL*	*clpP-psbB*	*trnL intron*	*rps16-trnQ*	4975	262.69	68.58
677	*psbB-psbT*	*ndhF-rpl32*	*rpl32-trnL*	*clpP-psbB*	*rpl16-rps3*	*trnE-trnT*	3939	267.95	69.26
678	*psbB-psbT*	*ndhF-rpl32*	*rpl32-trnL*	*clpP-psbB*	*rpl16-rps3*	*trnR-atpA*	3234	262.66	63.90
679	*psbB-psbT*	*ndhF-rpl32*	*rpl32-trnL*	*clpP-psbB*	*rpl16-rps3*	*rps16-trnQ*	4207	267.37	69.19
680	*psbB-psbT*	*ndhF-rpl32*	*rpl32-trnL*	*clpP-psbB*	*trnE-trnT*	*trnR-atpA*	3965	268.52	72.23
681	*psbB-psbT*	*ndhF-rpl32*	*rpl32-trnL*	*clpP-psbB*	*trnE-trnT*	*rps16-trnQ*	4938	273.23	72.16
682	*psbB-psbT*	*ndhF-rpl32*	*rpl32-trnL*	*clpP-psbB*	*trnR-atpA*	*rps16-trnQ*	4233	267.94	71.06
683	*psbB-psbT*	*ndhF-rpl32*	*rpl32-trnL*	*trnL intron*	*rpl16-rps3*	*trnE-trnT*	4059	266.25	69.00
684	*psbB-psbT*	*ndhF-rpl32*	*rpl32-trnL*	*trnL intron*	*rpl16-rps3*	*trnR-atpA*	3354	260.95	73.23
685	*psbB-psbT*	*ndhF-rpl32*	*rpl32-trnL*	*trnL intron*	*rpl16-rps3*	*rps16-trnQ*	4327	265.67	69.84
686	*psbB-psbT*	*ndhF-rpl32*	*rpl32-trnL*	*trnL intron*	*trnE-trnT*	*trnR-atpA*	4085	266.81	66.32
687	*psbB-psbT*	*ndhF-rpl32*	*rpl32-trnL*	*trnL intron*	*trnE-trnT*	*rps16-trnQ*	5058	271.52	74.32
688	*psbB-psbT*	*ndhF-rpl32*	*rpl32-trnL*	*trnL intron*	*trnR-atpA*	*rps16-trnQ*	4353	266.23	66.52
689	*psbB-psbT*	*ndhF-rpl32*	*rpl32-trnL*	*rpl16-rps3*	*trnE-trnT*	*trnR-atpA*	3317	271.49	69.42
690	*psbB-psbT*	*ndhF-rpl32*	*rpl32-trnL*	*rpl16-rps3*	*trnE-trnT*	*rps16-trnQ*	4290	276.21	69.19
691	*psbB-psbT*	*ndhF-rpl32*	*rpl32-trnL*	*rpl16-rps3*	*trnR-atpA*	*rps16-trnQ*	3585	270.91	69.06
692	*psbB-psbT*	*ndhF-rpl32*	*rpl32-trnL*	*trnE-trnT*	*trnR-atpA*	*rps16-trnQ*	4316	276.77	70.94
693	*psbB-psbT*	*ndhF-rpl32*	*clpP-psbB*	*trnL intron*	*rpl16-rps3*	*trnE-trnT*	4042	256.05	71.16
694	*psbB-psbT*	*ndhF-rpl32*	*clpP-psbB*	*trnL intron*	*rpl16-rps3*	*trnR-atpA*	3337	250.75	74.48
695	*psbB-psbT*	*ndhF-rpl32*	*clpP-psbB*	*trnL intron*	*rpl16-rps3*	*rps16-trnQ*	4310	255.47	66.84
696	*psbB-psbT*	*ndhF-rpl32*	*clpP-psbB*	*trnL intron*	*trnE-trnT*	*trnR-atpA*	4068	256.61	71.00
697	*psbB-psbT*	*ndhF-rpl32*	*clpP-psbB*	*trnL intron*	*trnE-trnT*	*rps16-trnQ*	5041	261.32	76.68
698	*psbB-psbT*	*ndhF-rpl32*	*clpP-psbB*	*trnL intron*	*trnR-atpA*	*rps16-trnQ*	4336	256.03	63.29
699	*psbB-psbT*	*ndhF-rpl32*	*clpP-psbB*	*rpl16-rps3*	*trnE-trnT*	*trnR-atpA*	3300	261.29	69.13
700	*psbB-psbT*	*ndhF-rpl32*	*clpP-psbB*	*rpl16-rps3*	*trnE-trnT*	*rps16-trnQ*	4273	266.01	71.77

701	*psbB-psbT*	*ndhF-rpl32*	*clpP-psbB*	*rpl16-rps3*	*trnR-atpA*	*rps16-trnQ*	3568	260.71	63.19
702	*psbB-psbT*	*ndhF-rpl32*	*clpP-psbB*	*trnE-trnT*	*trnR-atpA*	*rps16-trnQ*	4299	266.57	70.55
703	*psbB-psbT*	*ndhF-rpl32*	*trnL intron*	*rpl16-rps3*	*trnE-trnT*	*trnR-atpA*	3420	259.59	71.71
704	*psbB-psbT*	*ndhF-rpl32*	*trnL intron*	*rpl16-rps3*	*trnE-trnT*	*rps16-trnQ*	4393	264.30	76.45
705	*psbB-psbT*	*ndhF-rpl32*	*trnL intron*	*rpl16-rps3*	*trnR-atpA*	*rps16-trnQ*	3688	259.01	77.10
706	*psbB-psbT*	*ndhF-rpl32*	*trnL intron*	*trnE-trnT*	*trnR-atpA*	*rps16-trnQ*	4419	264.86	74.55
707	*psbB-psbT*	*ndhF-rpl32*	*rpl16-rps3*	*trnE-trnT*	*trnR-atpA*	*rps16-trnQ*	3651	269.55	68.77
708	*psbB-psbT*	*trnT-trnL*	*rpl32-trnL*	*clpP-psbB*	*trnL intron*	*rpl16-rps3*	4419	249.18	77.29
709	*psbB-psbT*	*trnT-trnL*	*rpl32-trnL*	*clpP-psbB*	*trnL intron*	*trnE-trnT*	5150	255.03	77.65
710	*psbB-psbT*	*trnT-trnL*	*rpl32-trnL*	*clpP-psbB*	*trnL intron*	*trnR-atpA*	4445	249.74	75.97
711	*psbB-psbT*	*trnT-trnL*	*rpl32-trnL*	*clpP-psbB*	*trnL intron*	*rps16-trnQ*	5418	254.45	75.74
712	*psbB-psbT*	*trnT-trnL*	*rpl32-trnL*	*clpP-psbB*	*rpl16-rps3*	*trnE-trnT*	4382	259.72	71.52
713	*psbB-psbT*	*trnT-trnL*	*rpl32-trnL*	*clpP-psbB*	*rpl16-rps3*	*trnR-atpA*	3677	254.42	63.94
714	*psbB-psbT*	*trnT-trnL*	*rpl32-trnL*	*clpP-psbB*	*rpl16-rps3*	*rps16-trnQ*	4650	259.14	73.19
715	*psbB-psbT*	*trnT-trnL*	*rpl32-trnL*	*clpP-psbB*	*trnE-trnT*	*trnR-atpA*	4408	260.28	73.00
716	*psbB-psbT*	*trnT-trnL*	*rpl32-trnL*	*clpP-psbB*	*trnE-trnT*	*rps16-trnQ*	5381	264.99	74.45
717	*psbB-psbT*	*trnT-trnL*	*rpl32-trnL*	*clpP-psbB*	*trnR-atpA*	*rps16-trnQ*	4676	259.70	71.68
718	*psbB-psbT*	*trnT-trnL*	*rpl32-trnL*	*trnL intron*	*rpl16-rps3*	*trnE-trnT*	4502	258.01	78.97
719	*psbB-psbT*	*trnT-trnL*	*rpl32-trnL*	*trnL intron*	*rpl16-rps3*	*trnR-atpA*	3797	252.71	78.48
720	*psbB-psbT*	*trnT-trnL*	*rpl32-trnL*	*trnL intron*	*rpl16-rps3*	*rps16-trnQ*	4770	257.43	77.48
721	*psbB-psbT*	*trnT-trnL*	*rpl32-trnL*	*trnL intron*	*trnE-trnT*	*trnR-atpA*	4528	258.57	78.23
722	*psbB-psbT*	*trnT-trnL*	*rpl32-trnL*	*trnL intron*	*trnE-trnT*	*rps16-trnQ*	5501	263.28	77.71
723	*psbB-psbT*	*trnT-trnL*	*rpl32-trnL*	*trnL intron*	*trnR-atpA*	*rps16-trnQ*	4796	257.99	76.26
724	*psbB-psbT*	*trnT-trnL*	*rpl32-trnL*	*rpl16-rps3*	*trnE-trnT*	*trnR-atpA*	3760	263.25	75.55
725	*psbB-psbT*	*trnT-trnL*	*rpl32-trnL*	*rpl16-rps3*	*trnE-trnT*	*rps16-trnQ*	4733	267.97	74.03
726	*psbB-psbT*	*trnT-trnL*	*rpl32-trnL*	*rpl16-rps3*	*trnR-atpA*	*rps16-trnQ*	4028	262.67	70.77
727	*psbB-psbT*	*trnT-trnL*	*rpl32-trnL*	*trnE-trnT*	*trnR-atpA*	*rps16-trnQ*	4759	268.53	75.58

728	*psbB-psbT*	*trnT-trnL*	*clpP-psbB*	*trnL intron*	*rpl16-rps3*	*trnE-trnT*	4485	247.81	73.81
729	*psbB-psbT*	*trnT-trnL*	*clpP-psbB*	*trnL intron*	*rpl16-rps3*	*trnR-atpA*	3780	242.52	68.42
730	*psbB-psbT*	*trnT-trnL*	*clpP-psbB*	*trnL intron*	*rpl16-rps3*	*rps16-trnQ*	4753	247.23	69.68
731	*psbB-psbT*	*trnT-trnL*	*clpP-psbB*	*trnL intron*	*trnE-trnT*	*trnR-atpA*	4511	248.37	76.00
732	*psbB-psbT*	*trnT-trnL*	*clpP-psbB*	*trnL intron*	*trnE-trnT*	*rps16-trnQ*	5484	253.08	76.97
733	*psbB-psbT*	*trnT-trnL*	*clpP-psbB*	*trnL intron*	*trnR-atpA*	*rps16-trnQ*	4779	247.79	70.52
734	*psbB-psbT*	*trnT-trnL*	*clpP-psbB*	*rpl16-rps3*	*trnE-trnT*	*trnR-atpA*	3743	253.06	69.16
735	*psbB-psbT*	*trnT-trnL*	*clpP-psbB*	*rpl16-rps3*	*trnE-trnT*	*rps16-trnQ*	4716	257.77	71.90
736	*psbB-psbT*	*trnT-trnL*	*clpP-psbB*	*rpl16-rps3*	*trnR-atpA*	*rps16-trnQ*	4011	252.48	67.26
737	*psbB-psbT*	*trnT-trnL*	*clpP-psbB*	*trnE-trnT*	*trnR-atpA*	*rps16-trnQ*	4742	258.33	71.16
738	*psbB-psbT*	*trnT-trnL*	*trnL intron*	*rpl16-rps3*	*trnE-trnT*	*trnR-atpA*	3863	251.35	76.77
739	*psbB-psbT*	*trnT-trnL*	*trnL intron*	*rpl16-rps3*	*trnE-trnT*	*rps16-trnQ*	4836	256.06	78.26
740	*psbB-psbT*	*trnT-trnL*	*trnL intron*	*rpl16-rps3*	*trnR-atpA*	*rps16-trnQ*	4131	250.77	70.16
741	*psbB-psbT*	*trnT-trnL*	*trnL intron*	*trnE-trnT*	*trnR-atpA*	*rps16-trnQ*	4862	256.62	77.06
742	*psbB-psbT*	*trnT-trnL*	*rpl16-rps3*	*trnE-trnT*	*trnR-atpA*	*rps16-trnQ*	4094	261.31	69.58
743	*psbB-psbT*	*rpl32-trnL*	*clpP-psbB*	*trnL intron*	*rpl16-rps3*	*trnE-trnT*	4516	249.32	68.55
744	*psbB-psbT*	*rpl32-trnL*	*clpP-psbB*	*trnL intron*	*rpl16-rps3*	*trnR-atpA*	3811	244.02	67.61
745	*psbB-psbT*	*rpl32-trnL*	*clpP-psbB*	*trnL intron*	*rpl16-rps3*	*rps16-trnQ*	4784	248.74	66.55
746	*psbB-psbT*	*rpl32-trnL*	*clpP-psbB*	*trnL intron*	*trnE-trnT*	*trnR-atpA*	4542	249.88	71.35
747	*psbB-psbT*	*rpl32-trnL*	*clpP-psbB*	*trnL intron*	*trnE-trnT*	*rps16-trnQ*	5515	254.59	75.77
748	*psbB-psbT*	*rpl32-trnL*	*clpP-psbB*	*trnL intron*	*trnR-atpA*	*rps16-trnQ*	4810	249.30	75.10
749	*psbB-psbT*	*rpl32-trnL*	*clpP-psbB*	*rpl16-rps3*	*trnE-trnT*	*trnR-atpA*	3774	254.56	62.16
750	*psbB-psbT*	*rpl32-trnL*	*clpP-psbB*	*rpl16-rps3*	*trnE-trnT*	*rps16-trnQ*	4747	259.28	69.55
751	*psbB-psbT*	*rpl32-trnL*	*clpP-psbB*	*rpl16-rps3*	*trnR-atpA*	*rps16-trnQ*	4042	253.98	68.26
752	*psbB-psbT*	*rpl32-trnL*	*clpP-psbB*	*trnE-trnT*	*trnR-atpA*	*rps16-trnQ*	4773	259.84	67.90
753	*psbB-psbT*	*rpl32-trnL*	*trnL intron*	*rpl16-rps3*	*trnE-trnT*	*trnR-atpA*	3894	252.86	73.48
754	*psbB-psbT*	*rpl32-trnL*	*trnL intron*	*rpl16-rps3*	*trnE-trnT*	*rps16-trnQ*	4867	257.57	76.35

755	*psbB-psbT*	*rpl32-trnL*	*trnL intron*	*rpl16-rps3*	*trnR-atpA*	*rps16-trnQ*	4162	252.27	70.10
756	*psbB-psbT*	*rpl32-trnL*	*trnL intron*	*trnE-trnT*	*trnR-atpA*	*rps16-trnQ*	4893	258.13	72.52
757	*psbB-psbT*	*rpl32-trnL*	*rpl16-rps3*	*trnE-trnT*	*trnR-atpA*	*rps16-trnQ*	4125	262.81	68.00
758	*psbB-psbT*	*clpP-psbB*	*trnL intron*	*rpl16-rps3*	*trnE-trnT*	*trnR-atpA*	3877	242.66	74.32
759	*psbB-psbT*	*clpP-psbB*	*trnL intron*	*rpl16-rps3*	*trnE-trnT*	*rps16-trnQ*	4850	247.37	73.97
760	*psbB-psbT*	*clpP-psbB*	*trnL intron*	*rpl16-rps3*	*trnR-atpA*	*rps16-trnQ*	4145	242.08	68.74
761	*psbB-psbT*	*clpP-psbB*	*trnL intron*	*trnE-trnT*	*trnR-atpA*	*rps16-trnQ*	4876	247.93	73.52
762	*psbB-psbT*	*clpP-psbB*	*rpl16-rps3*	*trnE-trnT*	*trnR-atpA*	*rps16-trnQ*	4108	252.62	67.26
763	*psbB-psbT*	*trnL intron*	*rpl16-rps3*	*trnE-trnT*	*trnR-atpA*	*rps16-trnQ*	4228	250.91	71.87
764	*ndhF-rpl32*	*trnT-trnL*	*rpl32-trnL*	*clpP-psbB*	*trnL intron*	*rpl16-rps3*	4136	237.09	84.52
765	*ndhF-rpl32*	*trnT-trnL*	*rpl32-trnL*	*clpP-psbB*	*trnL intron*	*trnE-trnT*	4867	242.94	74.81
766	*ndhF-rpl32*	*trnT-trnL*	*rpl32-trnL*	*clpP-psbB*	*trnL intron*	*trnR-atpA*	4162	237.65	82.00
767	*ndhF-rpl32*	*trnT-trnL*	*rpl32-trnL*	*clpP-psbB*	*trnL intron*	*rps16-trnQ*	5135	242.36	82.10
768	*ndhF-rpl32*	*trnT-trnL*	*rpl32-trnL*	*clpP-psbB*	*rpl16-rps3*	*trnE-trnT*	4099	247.63	73.13
769	*ndhF-rpl32*	*trnT-trnL*	*rpl32-trnL*	*clpP-psbB*	*rpl16-rps3*	*trnR-atpA*	3394	242.33	81.23
770	*ndhF-rpl32*	*trnT-trnL*	*rpl32-trnL*	*clpP-psbB*	*rpl16-rps3*	*rps16-trnQ*	4367	247.04	82.39
771	*ndhF-rpl32*	*trnT-trnL*	*rpl32-trnL*	*clpP-psbB*	*trnE-trnT*	*trnR-atpA*	4125	248.19	71.94
772	*ndhF-rpl32*	*trnT-trnL*	*rpl32-trnL*	*clpP-psbB*	*trnE-trnT*	*rps16-trnQ*	5098	252.90	76.74
773	*ndhF-rpl32*	*trnT-trnL*	*rpl32-trnL*	*clpP-psbB*	*trnR-atpA*	*rps16-trnQ*	4393	247.61	81.48
774	*ndhF-rpl32*	*trnT-trnL*	*rpl32-trnL*	*trnL intron*	*rpl16-rps3*	*trnE-trnT*	4219	245.92	75.45
775	*ndhF-rpl32*	*trnT-trnL*	*rpl32-trnL*	*trnL intron*	*rpl16-rps3*	*trnR-atpA*	3514	240.62	77.23
776	*ndhF-rpl32*	*trnT-trnL*	*rpl32-trnL*	*trnL intron*	*rpl16-rps3*	*rps16-trnQ*	4487	245.34	80.97
777	*ndhF-rpl32*	*trnT-trnL*	*rpl32-trnL*	*trnL intron*	*trnE-trnT*	*trnR-atpA*	4245	246.48	75.77
778	*ndhF-rpl32*	*trnT-trnL*	*rpl32-trnL*	*trnL intron*	*trnE-trnT*	*rps16-trnQ*	5218	251.19	76.90
779	*ndhF-rpl32*	*trnT-trnL*	*rpl32-trnL*	*trnL intron*	*trnR-atpA*	*rps16-trnQ*	4513	245.90	81.10
780	*ndhF-rpl32*	*trnT-trnL*	*rpl32-trnL*	*rpl16-rps3*	*trnE-trnT*	*trnR-atpA*	3477	251.16	68.32
781	*ndhF-rpl32*	*trnT-trnL*	*rpl32-trnL*	*rpl16-rps3*	*trnE-trnT*	*rps16-trnQ*	4450	255.88	76.23

782	*ndhF-rpl32*	*trnT-trnL*	*rpl32-trnL*	*rpl16-rps3*	*trnR-atpA*	*rps16-trnQ*	3745	250.58	77.77
783	*ndhF-rpl32*	*trnT-trnL*	*rpl32-trnL*	*trnE-trnT*	*trnR-atpA*	*rps16-trnQ*	4476	256.44	78.65
784	*ndhF-rpl32*	*trnT-trnL*	*clpP-psbB*	*trnL intron*	*rpl16-rps3*	*trnE-trnT*	4202	235.72	67.84
785	*ndhF-rpl32*	*trnT-trnL*	*clpP-psbB*	*trnL intron*	*rpl16-rps3*	*trnR-atpA*	3497	230.43	70.94
786	*ndhF-rpl32*	*trnT-trnL*	*clpP-psbB*	*trnL intron*	*rpl16-rps3*	*rps16-trnQ*	4470	235.14	71.94
787	*ndhF-rpl32*	*trnT-trnL*	*clpP-psbB*	*trnL intron*	*trnE-trnT*	*trnR-atpA*	4228	236.28	67.35
788	*ndhF-rpl32*	*trnT-trnL*	*clpP-psbB*	*trnL intron*	*trnE-trnT*	*rps16-trnQ*	5201	240.99	73.39
789	*ndhF-rpl32*	*trnT-trnL*	*clpP-psbB*	*trnL intron*	*trnR-atpA*	*rps16-trnQ*	4496	235.70	74.42
790	*ndhF-rpl32*	*trnT-trnL*	*clpP-psbB*	*rpl16-rps3*	*trnE-trnT*	*trnR-atpA*	3460	240.97	65.84
791	*ndhF-rpl32*	*trnT-trnL*	*clpP-psbB*	*rpl16-rps3*	*trnE-trnT*	*rps16-trnQ*	4433	245.68	67.81
792	*ndhF-rpl32*	*trnT-trnL*	*clpP-psbB*	*rpl16-rps3*	*trnR-atpA*	*rps16-trnQ*	3728	240.38	71.06
793	*ndhF-rpl32*	*trnT-trnL*	*clpP-psbB*	*trnE-trnT*	*trnR-atpA*	*rps16-trnQ*	4459	246.24	63.97
794	*ndhF-rpl32*	*trnT-trnL*	*trnL intron*	*rpl16-rps3*	*trnE-trnT*	*trnR-atpA*	3580	239.26	69.97
795	*ndhF-rpl32*	*trnT-trnL*	*trnL intron*	*rpl16-rps3*	*trnE-trnT*	*rps16-trnQ*	4553	243.97	71.55
796	*ndhF-rpl32*	*trnT-trnL*	*trnL intron*	*rpl16-rps3*	*trnR-atpA*	*rps16-trnQ*	3848	238.68	71.94
797	*ndhF-rpl32*	*trnT-trnL*	*trnL intron*	*trnE-trnT*	*trnR-atpA*	*rps16-trnQ*	4579	244.53	72.35
798	*ndhF-rpl32*	*trnT-trnL*	*rpl16-rps3*	*trnE-trnT*	*trnR-atpA*	*rps16-trnQ*	3811	249.22	66.13
799	*ndhF-rpl32*	*rpl32-trnL*	*clpP-psbB*	*trnL intron*	*rpl16-rps3*	*trnE-trnT*	4233	237.23	79.32
800	*ndhF-rpl32*	*rpl32-trnL*	*clpP-psbB*	*trnL intron*	*rpl16-rps3*	*trnR-atpA*	3528	231.93	79.00
801	*ndhF-rpl32*	*rpl32-trnL*	*clpP-psbB*	*trnL intron*	*rpl16-rps3*	*rps16-trnQ*	4501	236.65	81.06
802	*ndhF-rpl32*	*rpl32-trnL*	*clpP-psbB*	*trnL intron*	*trnE-trnT*	*trnR-atpA*	4259	237.79	79.84
803	*ndhF-rpl32*	*rpl32-trnL*	*clpP-psbB*	*trnL intron*	*trnE-trnT*	*rps16-trnQ*	5232	242.50	78.29
804	*ndhF-rpl32*	*rpl32-trnL*	*clpP-psbB*	*trnL intron*	*trnR-atpA*	*rps16-trnQ*	4527	237.21	83.26
805	*ndhF-rpl32*	*rpl32-trnL*	*clpP-psbB*	*rpl16-rps3*	*trnE-trnT*	*trnR-atpA*	3491	242.47	72.71
806	*ndhF-rpl32*	*rpl32-trnL*	*clpP-psbB*	*rpl16-rps3*	*trnE-trnT*	*rps16-trnQ*	4464	247.19	78.84
807	*ndhF-rpl32*	*rpl32-trnL*	*clpP-psbB*	*rpl16-rps3*	*trnR-atpA*	*rps16-trnQ*	3759	241.89	79.61
808	*ndhF-rpl32*	*rpl32-trnL*	*clpP-psbB*	*trnE-trnT*	*trnR-atpA*	*rps16-trnQ*	4490	247.75	78.45

809	*ndhF-rpl32*	*rpl32-trnL*	*trnL intron*	*rpl16-rps3*	*trnE-trnT*	*trnR-atpA*	3611	240.76	75.87
810	*ndhF-rpl32*	*rpl32-trnL*	*trnL intron*	*rpl16-rps3*	*trnE-trnT*	*rps16-trnQ*	4584	245.48	77.48
811	*ndhF-rpl32*	*rpl32-trnL*	*trnL intron*	*rpl16-rps3*	*trnR-atpA*	*rps16-trnQ*	3879	240.18	80.58
812	*ndhF-rpl32*	*rpl32-trnL*	*trnL intron*	*trnE-trnT*	*trnR-atpA*	*rps16-trnQ*	4610	246.04	80.23
813	*ndhF-rpl32*	*rpl32-trnL*	*rpl16-rps3*	*trnE-trnT*	*trnR-atpA*	*rps16-trnQ*	3842	250.72	77.61
814	*ndhF-rpl32*	*clpP-psbB*	*trnL intron*	*rpl16-rps3*	*trnE-trnT*	*trnR-atpA*	3594	230.57	73.32
815	*ndhF-rpl32*	*clpP-psbB*	*trnL intron*	*rpl16-rps3*	*trnE-trnT*	*rps16-trnQ*	4567	235.28	73.42
816	*ndhF-rpl32*	*clpP-psbB*	*trnL intron*	*rpl16-rps3*	*trnR-atpA*	*rps16-trnQ*	3862	229.99	75.65
817	*ndhF-rpl32*	*clpP-psbB*	*trnL intron*	*trnE-trnT*	*trnR-atpA*	*rps16-trnQ*	4593	235.84	73.39
818	*ndhF-rpl32*	*clpP-psbB*	*rpl16-rps3*	*trnE-trnT*	*trnR-atpA*	*rps16-trnQ*	3825	240.53	69.19
819	*ndhF-rpl32*	*trnL intron*	*rpl16-rps3*	*trnE-trnT*	*trnR-atpA*	*rps16-trnQ*	3945	238.82	76.81
820	*trnT-trnL*	*rpl32-trnL*	*clpP-psbB*	*trnL intron*	*rpl16-rps3*	*trnE-trnT*	4676	228.99	71.74
821	*trnT-trnL*	*rpl32-trnL*	*clpP-psbB*	*trnL intron*	*rpl16-rps3*	*trnR-atpA*	3971	223.70	79.19
822	*trnT-trnL*	*rpl32-trnL*	*clpP-psbB*	*trnL intron*	*rpl16-rps3*	*rps16-trnQ*	4944	228.41	81.97
823	*trnT-trnL*	*rpl32-trnL*	*clpP-psbB*	*trnL intron*	*trnE-trnT*	*trnR-atpA*	4702	229.55	73.45
824	*trnT-trnL*	*rpl32-trnL*	*clpP-psbB*	*trnL intron*	*trnE-trnT*	*rps16-trnQ*	5675	234.26	75.65
825	*trnT-trnL*	*rpl32-trnL*	*clpP-psbB*	*trnL intron*	*trnR-atpA*	*rps16-trnQ*	4970	228.97	80.94
826	*trnT-trnL*	*rpl32-trnL*	*clpP-psbB*	*rpl16-rps3*	*trnE-trnT*	*trnR-atpA*	3934	234.24	68.94
827	*trnT-trnL*	*rpl32-trnL*	*clpP-psbB*	*rpl16-rps3*	*trnE-trnT*	*rps16-trnQ*	4907	238.95	74.35
828	*trnT-trnL*	*rpl32-trnL*	*clpP-psbB*	*rpl16-rps3*	*trnR-atpA*	*rps16-trnQ*	4202	233.65	81.35
829	*trnT-trnL*	*rpl32-trnL*	*clpP-psbB*	*trnE-trnT*	*trnR-atpA*	*rps16-trnQ*	4933	239.51	73.97
830	*trnT-trnL*	*rpl32-trnL*	*trnL intron*	*rpl16-rps3*	*trnE-trnT*	*trnR-atpA*	4054	232.53	73.13
831	*trnT-trnL*	*rpl32-trnL*	*trnL intron*	*rpl16-rps3*	*trnE-trnT*	*rps16-trnQ*	5027	237.24	75.03
832	*trnT-trnL*	*rpl32-trnL*	*trnL intron*	*rpl16-rps3*	*trnR-atpA*	*rps16-trnQ*	4322	231.95	78.65
833	*trnT-trnL*	*rpl32-trnL*	*trnL intron*	*trnE-trnT*	*trnR-atpA*	*rps16-trnQ*	5053	237.80	76.71
834	*trnT-trnL*	*rpl32-trnL*	*rpl16-rps3*	*trnE-trnT*	*trnR-atpA*	*rps16-trnQ*	4285	242.49	73.55
835	*trnT-trnL*	*clpP-psbB*	*trnL intron*	*rpl16-rps3*	*trnE-trnT*	*trnR-atpA*	4037	222.33	67.58

836	*trnT-trnL*	*clpP-psbB*	*trnL intron*	*rpl16-rps3*	*trnE-trnT*	*rps16-trnQ*		5010	227.04	70.10
837	*trnT-trnL*	*clpP-psbB*	*trnL intron*	*rpl16-rps3*	*trnR-atpA*	*rps16-trnQ*		4305	221.75	72.32
838	*trnT-trnL*	*clpP-psbB*	*trnL intron*	*trnE-trnT*	*trnR-atpA*	*rps16-trnQ*		5036	227.60	69.10
839	*trnT-trnL*	*clpP-psbB*	*rpl16-rps3*	*trnE-trnT*	*trnR-atpA*	*rps16-trnQ*		4268	232.29	65.00
840	*trnT-trnL*	*trnL intron*	*rpl16-rps3*	*trnE-trnT*	*trnR-atpA*	*rps16-trnQ*		4388	230.58	70.03
841	*rpl32-trnL*	*clpP-psbB*	*trnL intron*	*rpl16-rps3*	*trnE-trnT*	*trnR-atpA*		4068	223.84	75.42
842	*rpl32-trnL*	*clpP-psbB*	*trnL intron*	*rpl16-rps3*	*trnE-trnT*	*rps16-trnQ*		5041	228.55	76.06
843	*rpl32-trnL*	*clpP-psbB*	*trnL intron*	*rpl16-rps3*	*trnR-atpA*	*rps16-trnQ*		4336	223.26	79.90
844	*rpl32-trnL*	*clpP-psbB*	*trnL intron*	*trnE-trnT*	*trnR-atpA*	*rps16-trnQ*		5067	229.11	78.26
845	*rpl32-trnL*	*clpP-psbB*	*rpl16-rps3*	*trnE-trnT*	*trnR-atpA*	*rps16-trnQ*		4299	233.80	75.84
846	*rpl32-trnL*	*trnL intron*	*rpl16-rps3*	*trnE-trnT*	*trnR-atpA*	*rps16-trnQ*		4419	232.09	75.19
847	*clpP-psbB*	*trnL intron*	*rpl16-rps3*	*trnE-trnT*	*trnR-atpA*	*rps16-trnQ*		4402	221.89	73.23
848	*psbB-psbT*	*ndhF-rpl32*	*trnT-trnL*	*rpl32-trnL*	*clpP-psbB*	*trnL intron*		4815	299.44	77.77
849	*psbB-psbT*	*ndhF-rpl32*	*trnT-trnL*	*rpl32-trnL*	*clpP-psbB*	*trnL intron*	*rpl16-rps3*	5546	305.29	79.39
850	*psbB-psbT*	*ndhF-rpl32*	*trnT-trnL*	*rpl32-trnL*	*clpP-psbB*	*trnL intron*	*trnE-trnT*	4841	300.00	76.32
851	*psbB-psbT*	*ndhF-rpl32*	*trnT-trnL*	*rpl32-trnL*	*clpP-psbB*	*trnL intron*	*trnR-atpA*	5814	304.71	78.74
852	*psbB-psbT*	*ndhF-rpl32*	*trnT-trnL*	*rpl32-trnL*	*clpP-psbB*	*rpl16-rps3*	*rps16-trnQ*	4778	309.98	73.48
853	*psbB-psbT*	*ndhF-rpl32*	*trnT-trnL*	*rpl32-trnL*	*clpP-psbB*	*rpl16-rps3*	*trnE-trnT*	4073	304.68	70.26
854	*psbB-psbT*	*ndhF-rpl32*	*trnT-trnL*	*rpl32-trnL*	*clpP-psbB*	*rpl16-rps3*	*trnR-atpA*	5046	309.40	75.06
855	*psbB-psbT*	*ndhF-rpl32*	*trnT-trnL*	*rpl32-trnL*	*clpP-psbB*	*trnE-trnT*	*rps16-trnQ*	4804	310.54	72.13
856	*psbB-psbT*	*ndhF-rpl32*	*trnT-trnL*	*rpl32-trnL*	*clpP-psbB*	*trnE-trnT*	*trnR-atpA*	5777	315.25	76.65
857	*psbB-psbT*	*ndhF-rpl32*	*trnT-trnL*	*rpl32-trnL*	*clpP-psbB*	*trnR-atpA*	*rps16-trnQ*	5072	309.96	75.45
858	*psbB-psbT*	*ndhF-rpl32*	*trnT-trnL*	*rpl32-trnL*	*trnL intron*	*rpl16-rps3*	*rps16-trnQ*	4898	308.27	80.58
859	*psbB-psbT*	*ndhF-rpl32*	*trnT-trnL*	*rpl32-trnL*	*trnL intron*	*rpl16-rps3*	*trnE-trnT*	4193	302.98	77.61
860	*psbB-psbT*	*ndhF-rpl32*	*trnT-trnL*	*rpl32-trnL*	*trnL intron*	*rpl16-rps3*	*trnR-atpA*	5166	307.69	78.00
861	*psbB-psbT*	*ndhF-rpl32*	*trnT-trnL*	*rpl32-trnL*	*trnL intron*	*trnE-trnT*	*rps16-trnQ*	4924	308.83	77.00
862	*psbB-psbT*	*ndhF-rpl32*	*trnT-trnL*	*rpl32-trnL*	*trnL intron*	*trnE-trnT*	*trnR-atpA*	5897	313.54	79.48

863	*psbB-psbT*	*ndhF-rpl32*	*trnT-trnL*	*rpl32-trnL*	*trnL intron*	*trnR-atpA*	*rps16-trnQ*	5192	308.25	77.77
864	*psbB-psbT*	*ndhF-rpl32*	*trnT-trnL*	*rpl32-trnL*	*rpl16-rps3*	*trnE-trnT*	*rps16-trnQ*	4156	313.52	75.26
865	*psbB-psbT*	*ndhF-rpl32*	*trnT-trnL*	*rpl32-trnL*	*rpl16-rps3*	*trnE-trnT*	*trnR-atpA*	5129	318.23	77.45
866	*psbB-psbT*	*ndhF-rpl32*	*trnT-trnL*	*rpl32-trnL*	*rpl16-rps3*	*trnR-atpA*	*rps16-trnQ*	4424	312.94	74.55
867	*psbB-psbT*	*ndhF-rpl32*	*trnT-trnL*	*rpl32-trnL*	*trnE-trnT*	*trnR-atpA*	*rps16-trnQ*	5155	318.79	75.87
868	*psbB-psbT*	*ndhF-rpl32*	*trnT-trnL*	*clpP-psbB*	*trnL intron*	*rpl16-rps3*	*rps16-trnQ*	4881	298.07	78.48
869	*psbB-psbT*	*ndhF-rpl32*	*trnT-trnL*	*clpP-psbB*	*trnL intron*	*rpl16-rps3*	*trnE-trnT*	4176	292.78	68.29
870	*psbB-psbT*	*ndhF-rpl32*	*trnT-trnL*	*clpP-psbB*	*trnL intron*	*rpl16-rps3*	*trnR-atpA*	5149	297.49	75.74
871	*psbB-psbT*	*ndhF-rpl32*	*trnT-trnL*	*clpP-psbB*	*trnL intron*	*trnE-trnT*	*rps16-trnQ*	4907	298.63	79.74
872	*psbB-psbT*	*ndhF-rpl32*	*trnT-trnL*	*clpP-psbB*	*trnL intron*	*trnE-trnT*	*trnR-atpA*	5880	303.35	80.52
873	*psbB-psbT*	*ndhF-rpl32*	*trnT-trnL*	*clpP-psbB*	*trnL intron*	*trnR-atpA*	*rps16-trnQ*	5175	298.05	74.84
874	*psbB-psbT*	*ndhF-rpl32*	*trnT-trnL*	*clpP-psbB*	*rpl16-rps3*	*trnE-trnT*	*rps16-trnQ*	4139	303.32	72.06
875	*psbB-psbT*	*ndhF-rpl32*	*trnT-trnL*	*clpP-psbB*	*rpl16-rps3*	*trnE-trnT*	*trnR-atpA*	5112	308.03	76.65
876	*psbB-psbT*	*ndhF-rpl32*	*trnT-trnL*	*clpP-psbB*	*rpl16-rps3*	*trnR-atpA*	*rps16-trnQ*	4407	302.74	72.58
877	*psbB-psbT*	*ndhF-rpl32*	*trnT-trnL*	*clpP-psbB*	*trnE-trnT*	*trnR-atpA*	*rps16-trnQ*	5138	308.59	73.94
878	*psbB-psbT*	*ndhF-rpl32*	*trnT-trnL*	*trnL intron*	*rpl16-rps3*	*trnE-trnT*	*rps16-trnQ*	4259	301.61	77.26
879	*psbB-psbT*	*ndhF-rpl32*	*trnT-trnL*	*trnL intron*	*rpl16-rps3*	*trnE-trnT*	*trnR-atpA*	5232	306.32	80.84
880	*psbB-psbT*	*ndhF-rpl32*	*trnT-trnL*	*trnL intron*	*rpl16-rps3*	*trnR-atpA*	*rps16-trnQ*	4527	301.03	74.26
881	*psbB-psbT*	*ndhF-rpl32*	*trnT-trnL*	*trnL intron*	*trnE-trnT*	*trnR-atpA*	*rps16-trnQ*	5258	306.88	81.55
882	*psbB-psbT*	*ndhF-rpl32*	*trnT-trnL*	*rpl16-rps3*	*trnE-trnT*	*trnR-atpA*	*rps16-trnQ*	4490	311.57	76.10
883	*psbB-psbT*	*ndhF-rpl32*	*rpl32-trnL*	*clpP-psbB*	*trnL intron*	*rpl16-rps3*	*rps16-trnQ*	4912	299.58	74.42
884	*psbB-psbT*	*ndhF-rpl32*	*rpl32-trnL*	*clpP-psbB*	*trnL intron*	*rpl16-rps3*	*trnE-trnT*	4207	294.29	66.77
885	*psbB-psbT*	*ndhF-rpl32*	*rpl32-trnL*	*clpP-psbB*	*trnL intron*	*rpl16-rps3*	*trnR-atpA*	5180	299.00	73.65
886	*psbB-psbT*	*ndhF-rpl32*	*rpl32-trnL*	*clpP-psbB*	*trnL intron*	*trnE-trnT*	*rps16-trnQ*	4938	300.14	70.94
887	*psbB-psbT*	*ndhF-rpl32*	*rpl32-trnL*	*clpP-psbB*	*trnL intron*	*trnE-trnT*	*trnR-atpA*	5911	304.85	78.45
888	*psbB-psbT*	*ndhF-rpl32*	*rpl32-trnL*	*clpP-psbB*	*trnL intron*	*trnR-atpA*	*rps16-trnQ*	5206	299.56	71.26
889	*psbB-psbT*	*ndhF-rpl32*	*rpl32-trnL*	*clpP-psbB*	*rpl16-rps3*	*trnE-trnT*	*rps16-trnQ*	4170	304.83	72.16

890	*psbB-psbT*	*ndhF-rpl32*	*rpl32-trnL*	*clpP-psbB*	*rpl16-rps3*	*trnE-trnT*	*trnR-atpA*	5143	309.54	72.35
891	*psbB-psbT*	*ndhF-rpl32*	*rpl32-trnL*	*clpP-psbB*	*rpl16-rps3*	*trnR-atpA*	*rps16-trnQ*	4438	304.24	70.16
892	*psbB-psbT*	*ndhF-rpl32*	*rpl32-trnL*	*clpP-psbB*	*trnE-trnT*	*trnR-atpA*	*rps16-trnQ*	5169	310.10	69.42
893	*psbB-psbT*	*ndhF-rpl32*	*rpl32-trnL*	*trnL intron*	*rpl16-rps3*	*trnE-trnT*	*rps16-trnQ*	4290	303.12	70.42
894	*psbB-psbT*	*ndhF-rpl32*	*rpl32-trnL*	*trnL intron*	*rpl16-rps3*	*trnE-trnT*	*trnR-atpA*	5263	307.83	75.55
895	*psbB-psbT*	*ndhF-rpl32*	*rpl32-trnL*	*trnL intron*	*rpl16-rps3*	*trnR-atpA*	*rps16-trnQ*	4558	302.54	68.68
896	*psbB-psbT*	*ndhF-rpl32*	*rpl32-trnL*	*trnL intron*	*trnE-trnT*	*trnR-atpA*	*rps16-trnQ*	5289	308.39	74.48
897	*psbB-psbT*	*ndhF-rpl32*	*rpl32-trnL*	*rpl16-rps3*	*trnE-trnT*	*trnR-atpA*	*rps16-trnQ*	4521	313.08	67.16
898	*psbB-psbT*	*ndhF-rpl32*	*clpP-psbB*	*trnL intron*	*rpl16-rps3*	*trnE-trnT*	*rps16-trnQ*	4273	292.92	72.32
899	*psbB-psbT*	*ndhF-rpl32*	*clpP-psbB*	*trnL intron*	*rpl16-rps3*	*trnE-trnT*	*trnR-atpA*	5246	297.63	79.77
900	*psbB-psbT*	*ndhF-rpl32*	*clpP-psbB*	*trnL intron*	*rpl16-rps3*	*trnR-atpA*	*rps16-trnQ*	4541	292.34	65.84
901	*psbB-psbT*	*ndhF-rpl32*	*clpP-psbB*	*trnL intron*	*trnE-trnT*	*trnR-atpA*	*rps16-trnQ*	5272	298.19	75.71
902	*psbB-psbT*	*ndhF-rpl32*	*clpP-psbB*	*rpl16-rps3*	*trnE-trnT*	*trnR-atpA*	*rps16-trnQ*	4504	302.88	70.26
903	*psbB-psbT*	*ndhF-rpl32*	*trnL intron*	*rpl16-rps3*	*trnE-trnT*	*trnR-atpA*	*rps16-trnQ*	4624	301.17	77.00
904	*psbB-psbT*	*trnT-trnL*	*rpl32-trnL*	*clpP-psbB*	*trnL intron*	*rpl16-rps3*	*rps16-trnQ*	5355	291.34	76.68
905	*psbB-psbT*	*trnT-trnL*	*rpl32-trnL*	*clpP-psbB*	*trnL intron*	*rpl16-rps3*	*trnE-trnT*	4650	286.05	77.19
906	*psbB-psbT*	*trnT-trnL*	*rpl32-trnL*	*clpP-psbB*	*trnL intron*	*rpl16-rps3*	*trnR-atpA*	5623	290.76	78.29
907	*psbB-psbT*	*trnT-trnL*	*rpl32-trnL*	*clpP-psbB*	*trnL intron*	*trnE-trnT*	*rps16-trnQ*	5381	291.90	79.71
908	*psbB-psbT*	*trnT-trnL*	*rpl32-trnL*	*clpP-psbB*	*trnL intron*	*trnE-trnT*	*trnR-atpA*	6354	296.62	81.19
909	*psbB-psbT*	*trnT-trnL*	*rpl32-trnL*	*clpP-psbB*	*trnL intron*	*trnR-atpA*	*rps16-trnQ*	5649	291.32	75.10
910	*psbB-psbT*	*trnT-trnL*	*rpl32-trnL*	*clpP-psbB*	*rpl16-rps3*	*trnE-trnT*	*rps16-trnQ*	4613	296.59	71.58
911	*psbB-psbT*	*trnT-trnL*	*rpl32-trnL*	*clpP-psbB*	*rpl16-rps3*	*trnE-trnT*	*trnR-atpA*	5586	301.30	75.94
912	*psbB-psbT*	*trnT-trnL*	*rpl32-trnL*	*clpP-psbB*	*rpl16-rps3*	*trnR-atpA*	*rps16-trnQ*	4881	296.01	72.13
913	*psbB-psbT*	*trnT-trnL*	*rpl32-trnL*	*clpP-psbB*	*trnE-trnT*	*trnR-atpA*	*rps16-trnQ*	5612	301.86	76.13
914	*psbB-psbT*	*trnT-trnL*	*rpl32-trnL*	*trnL intron*	*rpl16-rps3*	*trnE-trnT*	*rps16-trnQ*	4733	294.88	80.45
915	*psbB-psbT*	*trnT-trnL*	*rpl32-trnL*	*trnL intron*	*rpl16-rps3*	*trnE-trnT*	*trnR-atpA*	5706	299.59	79.71
916	*psbB-psbT*	*trnT-trnL*	*rpl32-trnL*	*trnL intron*	*rpl16-rps3*	*trnR-atpA*	*rps16-trnQ*	5001	294.30	70.71

917	*psbB-psbT*	*trnT-trnL*	*rpl32-trnL*	*trnL intron*	*trnE-trnT*	*trnR-atpA*	*rps16-trnQ*	5732	300.15	78.65
918	*psbB-psbT*	*trnT-trnL*	*rpl32-trnL*	*rpl16-rps3*	*trnE-trnT*	*trnR-atpA*	*rps16-trnQ*	4964	304.84	75.39
919	*psbB-psbT*	*trnT-trnL*	*clpP-psbB*	*trnL intron*	*rpl16-rps3*	*trnE-trnT*	*rps16-trnQ*	4716	284.68	78.52
920	*psbB-psbT*	*trnT-trnL*	*clpP-psbB*	*trnL intron*	*rpl16-rps3*	*trnE-trnT*	*trnR-atpA*	5689	289.39	77.81
921	*psbB-psbT*	*trnT-trnL*	*clpP-psbB*	*trnL intron*	*rpl16-rps3*	*trnR-atpA*	*rps16-trnQ*	4984	284.10	72.29
922	*psbB-psbT*	*trnT-trnL*	*clpP-psbB*	*trnL intron*	*trnE-trnT*	*trnR-atpA*	*rps16-trnQ*	5715	289.96	79.68
923	*psbB-psbT*	*trnT-trnL*	*clpP-psbB*	*rpl16-rps3*	*trnE-trnT*	*trnR-atpA*	*rps16-trnQ*	4947	294.64	74.32
924	*psbB-psbT*	*trnT-trnL*	*trnL intron*	*rpl16-rps3*	*trnE-trnT*	*trnR-atpA*	*rps16-trnQ*	5067	292.93	78.35
925	*psbB-psbT*	*rpl32-trnL*	*clpP-psbB*	*trnL intron*	*rpl16-rps3*	*trnE-trnT*	*rps16-trnQ*	4747	286.19	68.42
926	*psbB-psbT*	*rpl32-trnL*	*clpP-psbB*	*trnL intron*	*rpl16-rps3*	*trnE-trnT*	*trnR-atpA*	5720	290.90	76.13
927	*psbB-psbT*	*rpl32-trnL*	*clpP-psbB*	*trnL intron*	*rpl16-rps3*	*trnR-atpA*	*rps16-trnQ*	5015	285.61	67.52
928	*psbB-psbT*	*rpl32-trnL*	*clpP-psbB*	*trnL intron*	*trnE-trnT*	*trnR-atpA*	*rps16-trnQ*	5746	291.46	74.58
929	*psbB-psbT*	*rpl32-trnL*	*clpP-psbB*	*rpl16-rps3*	*trnE-trnT*	*trnR-atpA*	*rps16-trnQ*	4978	296.15	69.23
930	*psbB-psbT*	*rpl32-trnL*	*trnL intron*	*rpl16-rps3*	*trnE-trnT*	*trnR-atpA*	*rps16-trnQ*	5098	294.44	73.71
931	*psbB-psbT*	*clpP-psbB*	*trnL intron*	*rpl16-rps3*	*trnE-trnT*	*trnR-atpA*	*rps16-trnQ*	5081	284.24	73.06
932	*ndhF-rpl32*	*trnT-trnL*	*rpl32-trnL*	*clpP-psbB*	*trnL intron*	*rpl16-rps3*	*rps16-trnQ*	5072	279.25	76.39
933	*ndhF-rpl32*	*trnT-trnL*	*rpl32-trnL*	*clpP-psbB*	*trnL intron*	*rpl16-rps3*	*trnE-trnT*	4367	273.96	83.55
934	*ndhF-rpl32*	*trnT-trnL*	*rpl32-trnL*	*clpP-psbB*	*trnL intron*	*rpl16-rps3*	*trnR-atpA*	5340	278.67	82.52
935	*ndhF-rpl32*	*trnT-trnL*	*rpl32-trnL*	*clpP-psbB*	*trnL intron*	*trnE-trnT*	*rps16-trnQ*	5098	279.81	76.42
936	*ndhF-rpl32*	*trnT-trnL*	*rpl32-trnL*	*clpP-psbB*	*trnL intron*	*trnE-trnT*	*trnR-atpA*	6071	284.53	76.39
937	*ndhF-rpl32*	*trnT-trnL*	*rpl32-trnL*	*clpP-psbB*	*trnL intron*	*trnR-atpA*	*rps16-trnQ*	5366	279.23	81.23
938	*ndhF-rpl32*	*trnT-trnL*	*rpl32-trnL*	*clpP-psbB*	*rpl16-rps3*	*trnE-trnT*	*rps16-trnQ*	4330	284.50	73.39
939	*ndhF-rpl32*	*trnT-trnL*	*rpl32-trnL*	*clpP-psbB*	*rpl16-rps3*	*trnE-trnT*	*trnR-atpA*	5303	289.21	76.55
940	*ndhF-rpl32*	*trnT-trnL*	*rpl32-trnL*	*clpP-psbB*	*rpl16-rps3*	*trnR-atpA*	*rps16-trnQ*	4598	283.92	80.39
941	*ndhF-rpl32*	*trnT-trnL*	*rpl32-trnL*	*clpP-psbB*	*trnE-trnT*	*trnR-atpA*	*rps16-trnQ*	5329	289.77	77.74
942	*ndhF-rpl32*	*trnT-trnL*	*rpl32-trnL*	*trnL intron*	*rpl16-rps3*	*trnE-trnT*	*rps16-trnQ*	4450	282.79	74.32
943	*ndhF-rpl32*	*trnT-trnL*	*rpl32-trnL*	*trnL intron*	*rpl16-rps3*	*trnE-trnT*	*trnR-atpA*	5423	287.50	78.06

944	*ndhF-rpl32*	*trnT-trnL*	*rpl32-trnL*	*trnL intron*	*rpl16-rps3*	*trnR-atpA*	*rps16-trnQ*		4718	282.21	81.29
945	*ndhF-rpl32*	*trnT-trnL*	*rpl32-trnL*	*trnL intron*	*trnE-trnT*	*trnR-atpA*	*rps16-trnQ*		5449	288.06	76.03
946	*ndhF-rpl32*	*trnT-trnL*	*rpl32-trnL*	*rpl16-rps3*	*trnE-trnT*	*trnR-atpA*	*rps16-trnQ*		4681	292.75	77.94
947	*ndhF-rpl32*	*trnT-trnL*	*clpP-psbB*	*trnL intron*	*rpl16-rps3*	*trnE-trnT*	*rps16-trnQ*		4433	272.59	70.74
948	*ndhF-rpl32*	*trnT-trnL*	*clpP-psbB*	*trnL intron*	*rpl16-rps3*	*trnE-trnT*	*trnR-atpA*		5406	277.30	73.06
949	*ndhF-rpl32*	*trnT-trnL*	*clpP-psbB*	*trnL intron*	*rpl16-rps3*	*trnR-atpA*	*rps16-trnQ*		4701	272.01	75.13
950	*ndhF-rpl32*	*trnT-trnL*	*clpP-psbB*	*trnL intron*	*trnE-trnT*	*trnR-atpA*	*rps16-trnQ*		5432	277.87	75.16
951	*ndhF-rpl32*	*trnT-trnL*	*clpP-psbB*	*rpl16-rps3*	*trnE-trnT*	*trnR-atpA*	*rps16-trnQ*		4664	282.55	67.06
952	*ndhF-rpl32*	*trnT-trnL*	*trnL intron*	*rpl16-rps3*	*trnE-trnT*	*trnR-atpA*	*rps16-trnQ*		4784	280.84	71.90
953	*ndhF-rpl32*	*rpl32-trnL*	*clpP-psbB*	*trnL intron*	*rpl16-rps3*	*trnE-trnT*	*rps16-trnQ*		4464	274.10	78.94
954	*ndhF-rpl32*	*rpl32-trnL*	*clpP-psbB*	*trnL intron*	*rpl16-rps3*	*trnE-trnT*	*trnR-atpA*		5437	278.81	78.81
955	*ndhF-rpl32*	*rpl32-trnL*	*clpP-psbB*	*trnL intron*	*rpl16-rps3*	*trnR-atpA*	*rps16-trnQ*		4732	273.52	79.87
956	*ndhF-rpl32*	*rpl32-trnL*	*clpP-psbB*	*trnL intron*	*trnE-trnT*	*trnR-atpA*	*rps16-trnQ*		5463	279.37	79.52
957	*ndhF-rpl32*	*rpl32-trnL*	*clpP-psbB*	*rpl16-rps3*	*trnE-trnT*	*trnR-atpA*	*rps16-trnQ*		4695	284.06	77.68
958	*ndhF-rpl32*	*rpl32-trnL*	*trnL intron*	*rpl16-rps3*	*trnE-trnT*	*trnR-atpA*	*rps16-trnQ*		4815	282.35	78.45
959	*ndhF-rpl32*	*clpP-psbB*	*trnL intron*	*rpl16-rps3*	*trnE-trnT*	*trnR-atpA*	*rps16-trnQ*		4798	272.15	76.13
960	*trnT-trnL*	*rpl32-trnL*	*clpP-psbB*	*trnL intron*	*rpl16-rps3*	*trnE-trnT*	*rps16-trnQ*		4907	265.86	75.58
961	*trnT-trnL*	*rpl32-trnL*	*clpP-psbB*	*trnL intron*	*rpl16-rps3*	*trnE-trnT*	*trnR-atpA*		5880	270.57	75.19
962	*trnT-trnL*	*rpl32-trnL*	*clpP-psbB*	*trnL intron*	*rpl16-rps3*	*trnR-atpA*	*rps16-trnQ*		5175	265.28	83.00
963	*trnT-trnL*	*rpl32-trnL*	*clpP-psbB*	*trnL intron*	*trnE-trnT*	*trnR-atpA*	*rps16-trnQ*		5906	271.13	76.58
964	*trnT-trnL*	*rpl32-trnL*	*clpP-psbB*	*rpl16-rps3*	*trnE-trnT*	*trnR-atpA*	*rps16-trnQ*		5138	275.82	73.45
965	*trnT-trnL*	*rpl32-trnL*	*trnL intron*	*rpl16-rps3*	*trnE-trnT*	*trnR-atpA*	*rps16-trnQ*		5258	274.11	76.32
966	*trnT-trnL*	*clpP-psbB*	*trnL intron*	*rpl16-rps3*	*trnE-trnT*	*trnR-atpA*	*rps16-trnQ*		5241	263.91	71.58
967	*rpl32-trnL*	*clpP-psbB*	*trnL intron*	*rpl16-rps3*	*trnE-trnT*	*trnR-atpA*	*rps16-trnQ*		5272	265.42	74.16
968	*psbB-psbT*	*ndhF-rpl32*	*trnT-trnL*	*rpl32-trnL*	*clpP-psbB*	*trnL intron*	*rps16-trnQ*	*trnE-trnT*	5751	341.60	78.94
969	*psbB-psbT*	*ndhF-rpl32*	*trnT-trnL*	*rpl32-trnL*	*clpP-psbB*	*trnL intron*	*rpl16-rps3*	*trnR-atpA*	5046	336.31	76.71
970	*psbB-psbT*	*ndhF-rpl32*	*trnT-trnL*	*rpl32-trnL*	*clpP-psbB*	*trnL intron*	*rpl16-rps3*	*rps16-trnQ*	6019	341.02	80.00

971	*psbB-psbT*	*ndhF-rpl32*	*trnT-trnL*	*rpl32-trnL*	*clpP-psbB*	*trnL intron*	*rpl16-rps3*	*trnR-atpA*	5777	342.17	77.74
972	*psbB-psbT*	*ndhF-rpl32*	*trnT-trnL*	*rpl32-trnL*	*clpP-psbB*	*trnL intron*	*trnE-trnT*	*rps16-trnQ*	6750	346.88	80.97
973	*psbB-psbT*	*ndhF-rpl32*	*trnT-trnL*	*rpl32-trnL*	*clpP-psbB*	*trnL intron*	*trnE-trnT*	*rps16-trnQ*	6045	341.58	80.48
974	*psbB-psbT*	*ndhF-rpl32*	*trnT-trnL*	*rpl32-trnL*	*clpP-psbB*	*rpl16-rps3*	*trnR-atpA*	*trnR-atpA*	5009	346.85	76.97
975	*psbB-psbT*	*ndhF-rpl32*	*trnT-trnL*	*rpl32-trnL*	*clpP-psbB*	*rpl16-rps3*	*trnE-trnT*	*rps16-trnQ*	5982	351.56	78.19
976	*psbB-psbT*	*ndhF-rpl32*	*trnT-trnL*	*rpl32-trnL*	*clpP-psbB*	*rpl16-rps3*	*trnE-trnT*	*rps16-trnQ*	5277	346.27	77.84
977	*psbB-psbT*	*ndhF-rpl32*	*trnT-trnL*	*rpl32-trnL*	*clpP-psbB*	*trnE-trnT*	*trnR-atpA*	*rps16-trnQ*	6008	352.12	76.77
978	*psbB-psbT*	*ndhF-rpl32*	*trnT-trnL*	*rpl32-trnL*	*trnL intron*	*rpl16-rps3*	*trnR-atpA*	*trnR-atpA*	5129	345.14	79.26
979	*psbB-psbT*	*ndhF-rpl32*	*trnT-trnL*	*rpl32-trnL*	*trnL intron*	*rpl16-rps3*	*trnE-trnT*	*rps16-trnQ*	6102	349.85	80.23
980	*psbB-psbT*	*ndhF-rpl32*	*trnT-trnL*	*rpl32-trnL*	*trnL intron*	*rpl16-rps3*	*trnE-trnT*	*rps16-trnQ*	5397	344.56	77.35
981	*psbB-psbT*	*ndhF-rpl32*	*trnT-trnL*	*rpl32-trnL*	*trnL intron*	*trnE-trnT*	*trnR-atpA*	*rps16-trnQ*	6128	350.42	79.81
982	*psbB-psbT*	*ndhF-rpl32*	*trnT-trnL*	*rpl32-trnL*	*rpl16-rps3*	*trnE-trnT*	*trnR-atpA*	*rps16-trnQ*	5360	355.10	80.61
983	*psbB-psbT*	*ndhF-rpl32*	*trnT-trnL*	*clpP-psbB*	*trnL intron*	*rpl16-rps3*	*trnR-atpA*	*trnR-atpA*	5112	334.94	80.23
984	*psbB-psbT*	*ndhF-rpl32*	*trnT-trnL*	*clpP-psbB*	*trnL intron*	*rpl16-rps3*	*trnE-trnT*	*rps16-trnQ*	6085	339.66	82.32
985	*psbB-psbT*	*ndhF-rpl32*	*trnT-trnL*	*clpP-psbB*	*trnL intron*	*rpl16-rps3*	*trnE-trnT*	*rps16-trnQ*	5380	334.36	75.97
986	*psbB-psbT*	*ndhF-rpl32*	*trnT-trnL*	*clpP-psbB*	*trnL intron*	*trnE-trnT*	*trnR-atpA*	*rps16-trnQ*	6111	340.22	82.58
987	*psbB-psbT*	*ndhF-rpl32*	*trnT-trnL*	*clpP-psbB*	*rpl16-rps3*	*trnE-trnT*	*trnR-atpA*	*rps16-trnQ*	5343	344.90	74.90
988	*psbB-psbT*	*ndhF-rpl32*	*trnT-trnL*	*trnL intron*	*rpl16-rps3*	*trnE-trnT*	*trnR-atpA*	*rps16-trnQ*	5463	343.19	82.16
989	*psbB-psbT*	*ndhF-rpl32*	*rpl32-trnL*	*clpP-psbB*	*trnL intron*	*rpl16-rps3*	*trnR-atpA*	*trnR-atpA*	5143	336.45	73.58
990	*psbB-psbT*	*ndhF-rpl32*	*rpl32-trnL*	*clpP-psbB*	*trnL intron*	*rpl16-rps3*	*trnE-trnT*	*rps16-trnQ*	6116	341.16	78.74
991	*psbB-psbT*	*ndhF-rpl32*	*rpl32-trnL*	*clpP-psbB*	*trnL intron*	*rpl16-rps3*	*trnE-trnT*	*rps16-trnQ*	5411	335.87	73.58
992	*psbB-psbT*	*ndhF-rpl32*	*rpl32-trnL*	*clpP-psbB*	*trnL intron*	*trnE-trnT*	*trnR-atpA*	*rps16-trnQ*	6142	341.73	76.48
993	*psbB-psbT*	*ndhF-rpl32*	*rpl32-trnL*	*clpP-psbB*	*rpl16-rps3*	*trnE-trnT*	*trnR-atpA*	*rps16-trnQ*	5374	346.41	73.81
994	*psbB-psbT*	*ndhF-rpl32*	*rpl32-trnL*	*trnL intron*	*rpl16-rps3*	*trnE-trnT*	*trnR-atpA*	*rps16-trnQ*	5494	344.70	77.45
995	*psbB-psbT*	*ndhF-rpl32*	*clpP-psbB*	*trnL intron*	*rpl16-rps3*	*trnE-trnT*	*trnR-atpA*	*rps16-trnQ*	5477	334.50	77.90
996	*psbB-psbT*	*trnT-trnL*	*rpl32-trnL*	*clpP-psbB*	*trnL intron*	*rpl16-rps3*	*trnR-atpA*	*trnR-atpA*	5586	328.21	81.61
997	*psbB-psbT*	*trnT-trnL*	*rpl32-trnL*	*clpP-psbB*	*trnL intron*	*rpl16-rps3*	*trnE-trnT*	*rps16-trnQ*	6559	332.93	80.39

998	*psbB-psbT*	*trnT-trnL*	*rpl32-trnL*	*clpP-psbB*	*trnL intron*	*rpl16-rps3*	*trnE-trnT*	*rps16-trnQ*			5854	327.63	78.32
999	*psbB-psbT*	*trnT-trnL*	*rpl32-trnL*	*clpP-psbB*	*trnL intron*	*trnE-trnT*	*trnR-atpA*	*rps16-trnQ*			6585	333.49	80.77
1000	*psbB-psbT*	*trnT-trnL*	*rpl32-trnL*	*clpP-psbB*	*rpl16-rps3*	*trnE-trnT*	*trnR-atpA*	*rps16-trnQ*			5817	338.17	76.87
1001	*psbB-psbT*	*trnT-trnL*	*rpl32-trnL*	*trnL intron*	*rpl16-rps3*	*trnE-trnT*	*trnR-atpA*	*rps16-trnQ*			5937	336.46	81.13
1002	*psbB-psbT*	*trnT-trnL*	*clpP-psbB*	*trnL intron*	*rpl16-rps3*	*trnE-trnT*	*trnR-atpA*	*rps16-trnQ*			5920	326.27	78.94
1003	*psbB-psbT*	*rpl32-trnL*	*clpP-psbB*	*trnL intron*	*rpl16-rps3*	*trnE-trnT*	*trnR-atpA*	*rps16-trnQ*			5951	327.77	76.65
1004	*ndhF-rpl32*	*trnT-trnL*	*rpl32-trnL*	*clpP-psbB*	*trnL intron*	*rpl16-rps3*	*trnR-atpA*	*trnR-atpA*			5303	316.12	74.90
1005	*ndhF-rpl32*	*trnT-trnL*	*rpl32-trnL*	*clpP-psbB*	*trnL intron*	*rpl16-rps3*	*trnE-trnT*	*rps16-trnQ*			6276	320.83	76.81
1006	*ndhF-rpl32*	*trnT-trnL*	*rpl32-trnL*	*clpP-psbB*	*trnL intron*	*rpl16-rps3*	*trnE-trnT*	*rps16-trnQ*			5571	315.54	81.39
1007	*ndhF-rpl32*	*trnT-trnL*	*rpl32-trnL*	*clpP-psbB*	*trnL intron*	*trnE-trnT*	*trnR-atpA*	*rps16-trnQ*			6302	321.40	78.48
1008	*ndhF-rpl32*	*trnT-trnL*	*rpl32-trnL*	*clpP-psbB*	*rpl16-rps3*	*trnE-trnT*	*trnR-atpA*	*rps16-trnQ*			5534	326.08	76.65
1009	*ndhF-rpl32*	*trnT-trnL*	*rpl32-trnL*	*trnL intron*	*rpl16-rps3*	*trnE-trnT*	*trnR-atpA*	*rps16-trnQ*			5654	324.37	77.23
1010	*ndhF-rpl32*	*trnT-trnL*	*clpP-psbB*	*trnL intron*	*rpl16-rps3*	*trnE-trnT*	*trnR-atpA*	*rps16-trnQ*			5637	314.17	72.84
1011	*ndhF-rpl32*	*rpl32-trnL*	*clpP-psbB*	*trnL intron*	*rpl16-rps3*	*trnE-trnT*	*trnR-atpA*	*rps16-trnQ*			5668	315.68	77.77
1012	*trnT-trnL*	*rpl32-trnL*	*clpP-psbB*	*trnL intron*	*rpl16-rps3*	*trnE-trnT*	*trnR-atpA*	*rps16-trnQ*			6111	307.44	76.42
1013	*psbB-psbT*	*ndhF-rpl32*	*trnT-trnL*	*rpl32-trnL*	*clpP-psbB*	*trnL intron*	*trnR-atpA*	*trnE-trnT*	*trnR-atpA*		5982	378.47	77.94
1014	*psbB-psbT*	*ndhF-rpl32*	*trnT-trnL*	*rpl32-trnL*	*clpP-psbB*	*trnL intron*	*rpl16-rps3*	*trnE-trnT*	*rps16-trnQ*		6955	383.19	81.03
1015	*psbB-psbT*	*ndhF-rpl32*	*trnT-trnL*	*rpl32-trnL*	*clpP-psbB*	*trnL intron*	*rpl16-rps3*	*trnR-atpA*	*rps16-trnQ*		6250	377.89	78.03
1016	*psbB-psbT*	*ndhF-rpl32*	*trnT-trnL*	*rpl32-trnL*	*clpP-psbB*	*trnL intron*	*rpl16-rps3*	*trnR-atpA*	*rps16-trnQ*		6981	383.75	81.55
1017	*psbB-psbT*	*ndhF-rpl32*	*trnT-trnL*	*rpl32-trnL*	*clpP-psbB*	*rpl16-rps3*	*trnE-trnT*	*trnR-atpA*	*rps16-trnQ*		6213	388.43	82.19
1018	*psbB-psbT*	*ndhF-rpl32*	*trnT-trnL*	*rpl32-trnL*	*trnL intron*	*rpl16-rps3*	*trnE-trnT*	*trnR-atpA*	*rps16-trnQ*		6333	386.73	81.23
1019	*psbB-psbT*	*ndhF-rpl32*	*trnT-trnL*	*clpP-psbB*	*trnL intron*	*rpl16-rps3*	*trnE-trnT*	*trnR-atpA*	*rps16-trnQ*		6316	376.53	81.00
1020	*psbB-psbT*	*ndhF-rpl32*	*rpl32-trnL*	*clpP-psbB*	*trnL intron*	*rpl16-rps3*	*trnE-trnT*	*trnR-atpA*	*rps16-trnQ*		6347	378.04	77.84
1021	*psbB-psbT*	*trnT-trnL*	*rpl32-trnL*	*clpP-psbB*	*trnL intron*	*rpl16-rps3*	*trnE-trnT*	*trnR-atpA*	*rps16-trnQ*		6790	369.80	79.48
1022	*ndhF-rpl32*	*trnT-trnL*	*rpl32-trnL*	*clpP-psbB*	*trnL intron*	*rpl16-rps3*	*trnE-trnT*	*trnR-atpA*	*rps16-trnQ*		6507	357.71	82.65
1023	*psbB-psbT*	*ndhF-rpl32*	*trnT-trnL*	*rpl32-trnL*	*clpP-psbB*	*trnL intron*	*trnE-trnT*	*trnE-trnT*	*trnR-atpA*	*rps16-trnQ*	7186	420.06	82.48

Table S5 Top-ten hotspot combinations with the highest bootstrap (BS) values

No.	Combination										BS value
1	*ndhF-rpl32*	*trnT-trnL*	*rpl32-trnL*	*clpP-psbB*	*trnL intron*	*rpl16-rps3*					84.51613
2	*ndhF-rpl32*	*trnT-trnL*	*rpl32-trnL*	*clpP-psbB*	*trnL intron*	*rpl16-rps3*	*trnE-trnT*				83.54839
3	*ndhF-rpl32*	*rpl32-trnL*	*clpP-psbB*	*trnL intron*	*trnR-atpA*	*rps16-trnQ*					83.25806
4	*trnT-trnL*	*rpl32-trnL*	*clpP-psbB*	*trnL intron*	*rps16-trnQ*						83.09677
5	*trnT-trnL*	*rpl32-trnL*	*clpP-psbB*	*trnL intron*	*rpl16-rps3*	*trnR-atpA*	*rps16-trnQ*				83.00000
6	*ndhF-rpl32*	*trnT-trnL*	*rpl32-trnL*	*clpP-psbB*	*trnL intron*	*rpl16-rps3*	*trnE-trnT*	*trnR-atpA*	*rps16-trnQ*		82.64516
7	*psbB-psbT*	*ndhF-rpl32*	*trnT-trnL*	*clpP-psbB*	*trnL intron*	*trnE-trnT*	*trnR-atpA*	*rps16-trnQ*			82.58065
8	*ndhF-rpl32*	*trnT-trnL*	*rpl32-trnL*	*clpP-psbB*	*trnL intron*	*rpl16-rps3*	*trnR-atpA*				82.51613
9	*psbB-psbT*	*ndhF-rpl32*	*trnT-trnL*	*rpl32-trnL*	*clpP-psbB*	*trnL intron*	*trnE-trnT*	*trnE-trnT*	*trnR-atpA*	*rps16-trnQ*	82.48387
10	*ndhF-rpl32*	*trnT-trnL*	*rpl32-trnL*	*clpP-psbB*	*rpl16-rps3*	*rps16-trnQ*					82.38710
